# Burden of non-communicable diseases among adolescents aged 10–24 years in the EU, 1990–2019: a systematic analysis of the Global Burden of Diseases Study 2019

**DOI:** 10.1016/S2352-4642(22)00073-6

**Published:** 2022-06

**Authors:** Benedetta Armocida, Lorenzo Monasta, Susan Sawyer, Flavia Bustreo, Giulia Segafredo, Giulio Castelpietra, Luca Ronfani, Maja Pasovic, Simon Hay, GBD 2019 Europe NCDs in Adolescents Collaborators, Benedetta Armocida, Lorenzo Monasta, Susan M Sawyer, Flavia Bustreo, Giulia Segafredo, Giulio Castelpietra, Luca Ronfani, Maja Pasovic, Simon I Hay, Derrick Bary Abila, Hassan Abolhassani, Manfred Mario Kokou Accrombessi, Victor Adekanmbi, Keivan Ahmadi, Hanadi Al Hamad, Mamoon A Aldeyab, Adel Al-Jumaily, Robert Ancuceanu, Catalina Liliana Andrei, Tudorel Andrei, Ashokan Arumugam, Sameh Attia, Avinash Aujayeb, Marcel Ausloos, Jennifer L Baker, Francesco Barone-Adesi, Fabio Barra, Sandra Barteit, Sanjay Basu, Bernhard T Baune, Yannick Béjot, Luis Belo, Derrick A Bennett, Boris Bikbov, Andras Bikov, Oleg Blyuss, Susanne Breitner, Hermann Brenner, Giulia Carreras, Márcia Carvalho, Alberico L Catapano, Joht Singh Chandan, Periklis Charalampous, Simiao Chen, Joao Conde, Natália Cruz-Martins, Giovanni Damiani, Anna Dastiridou, Alejandro de la Torre-Luque, Mostafa Dianatinasab, Diana Dias da Silva, Abdel Douiri, Elena Dragioti, Luchuo Engelbert Bain, Adeniyi Francis Fagbamigbe, Seyed-Mohammad Fereshtehnejad, Pietro Ferrara, José Miguel P Ferreira de Oliveira, Simone Ferrero, Lorenzo Ferro Desideri, Florian Fischer, Diogo A Fonseca, Piyada Gaewkhiew, Santosh Gaihre, Silvano Gallus, Mariana Gaspar Fonseca, Paramjit Singh Gill, James C Glasbey, Giuseppe Gorini, Vijai Kumar Gupta, Mekdes Kondale Gurara, Josep Maria Haro, M Tasdik Hasan, Rasmus J Havmoeller, Behzad Heibati, Merel E Hellemons, Claudiu Herteliu, Salman Hussain, Gaetano Isola, Olatunji Johnson, Jost B Jonas, Jacek Jerzy Jozwiak, Mikk Jürisson, Zubair Kabir, André Karch, Joonas H Kauppila, Gbenga A Kayode, Moien AB Khan, Khaled Khatab, Mika Kivimäki, Miloslav Klugar, Jitka Klugarová, Kamrun Nahar Koly, Ai Koyanagi, Om P Kurmi, Dian Kusuma, Carlo La Vecchia, Ben Lacey, Tea Lallukka, Demetris Lamnisos, Berthold Langguth, Anders O Larsson, Paolo Lauriola, Paul H Lee, Matilde Leonardi, An Li, Christine Linehan, Rubén López-Bueno, Stefan Lorkowski, Joana A Loureiro, Raimundas Lunevicius, Laura A Magee, Francesca Giulia Magnani, Azeem Majeed, Konstantinos Christos Makris, Alexander G Mathioudakis, Manu Raj Mathur, John J McGrath, Ritesh G Menezes, Alexios-Fotios A Mentis, Atte Meretoja, Tomislav Mestrovic, Junmei Miao Jonasson, Tomasz Miazgowski, Andreea Mirica, Marcello Moccia, Shafiu Mohammed, Mariam Molokhia, Stefania Mondello, Ulrich Otto Mueller, Francesk Mulita, Daniel Munblit, Ionut Negoi, Ruxandra Irina Negoi, Evangelia Nena, Nurulamin M Noor, Christoph Nowak, George Ntaios, Vincent Ebuka Nwatah, Bogdan Oancea, Ayodipupo Sikiru Oguntade, Alberto Ortiz, Adrian Otoiu, Alicia Padron-Monedero, Raffaele Palladino, Adrian Pana, Demosthenes Panagiotakos, Songhomitra Panda-Jonas, Shahina Pardhan, Jay Patel, Paolo Pedersini, José L Peñalvo, Umberto Pensato, Renato B Pereira, Norberto Perico, Ionela-Roxana Petcu, Suzanne Polinder, Maarten J Postma, Mohammad Rabiee, Navid Rabiee, Alberto Raggi, Shadi Rahimzadeh, David Laith Rawaf, Salman Rawaf, Faizan Ur Rehman, Giuseppe Remuzzi, Abanoub Riad, Alina Rodriguez, Simona Sacco, Mohammad Reza Saeb, Mahdi Safdarian, Brijesh Sathian, Davide Sattin, Sonia Saxena, Nikolaos Scarmeas, Winfried Schlee, Falk Schwendicke, Morteza Shamsizadeh, Nigussie Tadesse Sharew, Rahman Shiri, Siddharudha Shivalli, Velizar Shivarov, João Pedro Silva, Colin R Simpson, Søren T Skou, Bogdan Socea, Ireneous N Soyiri, Paschalis Steiropoulos, Kurt Straif, Xiaohui Sun, Rafael Tabarés-Seisdedos, Arulmani Thiyagarajan, Fotis Topouzis, Marcos Roberto Tovani-Palone, Thomas Clement Truelsen, Brigid Unim, Jef Van den Eynde, Tommi Juhani Vasankari, Massimiliano Veroux, Santos Villafaina, Matej Vinko, Francesco S Violante, Victor Volovici, Yanzhong Wang, Ronny Westerman, Mohammad Esmaeil Yadegarfar, Sanni Yaya, Vesna Zadnik, Alimuddin Zumla, Pablo Perel, David Beran, Pablo Perel, David Beran

**Affiliations:** a Division of Tropical and Humanitarian Medicine, University of Geneva, Geneva, Switzerland; b Institute for Maternal and Child Health IRCCS Burlo Garofolo, Trieste, Italy; c Department of Paediatrics, University of Melbourne, Melbourne, VIC, Australia; d Murdoch Children's Research Institute, Melbourne, VIC, Australia; e Centre for Adolescent Health, Royal Children's Hospital Melbourne, Melbourne, VIC, Australia; f Fondation Botnar, Geneva, Switzerland; g Medicines Patent Pool, Geneva, Switzerland; h Outpatient and Inpatient Care Service, Central Health Directorate, Friuli Venezia Giulia Region, Trieste, Italy; i Institute for Health Metrics and Evaluation, University of Washington, Seattle, WA, USA; j Department of Non-Communicable Disease Epidemiology, London School of Hygiene and Tropical Medicine, London, UK; k Division of Tropical and Humanitarian Medicine, University of Geneva and Geneva University Hospitals, Switzerland

## Abstract

**Background:**

Disability and mortality burden of non-communicable diseases (NCDs) have risen worldwide; however, the NCD burden among adolescents remains poorly described in the EU.

**Methods:**

Estimates were retrieved from the Global Burden of Diseases, Injuries, and Risk Factors Study (GBD) 2019. Causes of NCDs were analysed at three different levels of the GBD 2019 hierarchy, for which mortality, years of life lost (YLLs), years lived with disability (YLDs), and disability-adjusted life-years (DALYs) were extracted. Estimates, with the 95% uncertainty intervals (UI), were retrieved for EU Member States from 1990 to 2019, three age subgroups (10–14 years, 15–19 years, and 20–24 years), and by sex. Spearman's correlation was conducted between DALY rates for NCDs and the Socio-demographic Index (SDI) of each EU Member State.

**Findings:**

In 2019, NCDs accounted for 86·4% (95% uncertainty interval 83·5–88·8) of all YLDs and 38·8% (37·4–39·8) of total deaths in adolescents aged 10–24 years. For NCDs in this age group, neoplasms were the leading causes of both mortality (4·01 [95% uncertainty interval 3·62–4·25] per 100 000 population) and YLLs (281·78 [254·25–298·92] per 100 000 population), whereas mental disorders were the leading cause for YLDs (2039·36 [1432·56–2773·47] per 100 000 population) and DALYs (2040·59 [1433·96–2774·62] per 100 000 population) in all EU Member States, and in all studied age groups. In 2019, among adolescents aged 10–24 years, males had a higher mortality rate per 100 000 population due to NCDs than females (11·66 [11·04–12·28] *vs* 7·89 [7·53–8·23]), whereas females presented a higher DALY rate per 100 000 population due to NCDs (8003·25 [5812·78–10 701·59] *vs* 6083·91 [4576·63–7857·92]). From 1990 to 2019, mortality rate due to NCDs in adolescents aged 10–24 years substantially decreased (–40·41% [–43·00 to –37·61), and also the YLL rate considerably decreased (–40·56% [–43·16 to –37·74]), except for mental disorders (which increased by 32·18% [1·67 to 66·49]), whereas the YLD rate increased slightly (1·44% [0·09 to 2·79]). Positive correlations were observed between DALY rates and SDIs for substance use disorders (*r*_s_=0·58, p=0·0012) and skin and subcutaneous diseases (*r*_s_=0·45, p=0·017), whereas negative correlations were found between DALY rates and SDIs for cardiovascular diseases (*r*_s_=–0·46, p=0·015), neoplasms (*r*_s_=–0·57, p=0·0015), and sense organ diseases (*r*_s_=–0·61, p=0·0005).

**Interpretation:**

NCD-related mortality has substantially declined among adolescents in the EU between 1990 and 2019, but the rising trend of YLL attributed to mental disorders and their YLD burden are concerning. Differences by sex, age group, and across EU Member States highlight the importance of preventive interventions and scaling up adolescent-responsive health-care systems, which should prioritise specific needs by sex, age, and location.

**Funding:**

Bill & Melinda Gates Foundation.

## Introduction

Adolescence is a period of major physical growth, psychological development, and shifting social relationships, with major repercussions for health.^1^ The inclusion of adolescents within the Global Strategy for Women's, Children's, and Adolescents' Health^2^ and the Countdown to 2030,^3^ has reinforced the importance of tracking adolescent health. However, so far, global progress has been slow,^4^ and adolescents remain a neglected age group in the quest for universal health coverage.^5^ In this context, the scarcity of adolescent-specific country data, disaggregated by sex and age, is a major barrier.^5^ The non-communicable disease (NCD) agenda has so far predominantly focused on adults,^6^, ^7^ reflecting historical assumptions of adolescents as largely healthy. However, globally among adolescents, the burden of disability and mortality from NCDs has risen substantially,^8^ with the leading causes being mental disorders, substance use disorders, and chronic physical illness.^9^

EU Member States, although committed to addressing certain issues in adolescent health, particularly mental health and wellbeing,^10^ are yet to conduct a broad assessment of the disability and mortality burden of NCDs among adolescents. Although most EU Member States have high economic development and relatively high-quality health services, and fall into the category of NCD-predominant countries,^9^ differences in culture, governance, and prioritisation of public health policies and investments mean it is a region where changing NCD profiles among adolescents can be explored. Moreover, despite prevention policies implemented in the EU leading to progress in the reduction of premature mortality from NCDs, such as control measures targeting tobacco products, alcoholic beverages, and unhealthy food for young people, about a third of the EU population aged 15 years or older still lives with an NCD, and €700 billion is spent on treating NCDs annually in the region.^11^ Given considerable heterogeneity and inconsistency in data collection systems and major data gaps, we used estimates from the Global Burden of Diseases, Injuries, and Risk Factors Study (GBD) 2019 to: provide a comprehensive assessment of the burden of mortality and disability due to NCDs in adolescents aged 10–24 years in EU Member States by cause, sex, age, location, and trend over time for the 30-year period; and evaluate the association between the disability-adjusted life-year (DALY) rates, which comprise both years lived with disability (YLDs) and years of life lost (YLLs), of the NCDs with the developmental stage of each EU Member State, using a composite measure of income per capita, average educational attainment, and fertility rate. Growing concerns about the effects of the COVID-19 pandemic and its containment measures on NCDs and their risk factors among adolescents^12^, ^13^ suggest that this assessment can be considered a pre-pandemic baseline from which subsequent data can be compared at the regional and country level.

**Research in context**

**Evidence before this study**

We searched Embase and PubMed for research articles published in English on Nov 22, 2021, using the following terms in titles or abstracts: (“adolescent” OR “young people”) AND (“disability” OR “mortality”) AND (“Europe” OR “European Union”) AND (“non communicable disease” OR “NCD”). Although we identified several studies, these primarily examined adolescent mortality, mainly at the global level, and did not specifically focus on non-communicable diseases (NCDs). Moreover, studies either included smaller age groups (10–14 years, 10–19 years) or were country specific, disease specific, or part of a wider study on mortality or disability burden in other age groups. We only found one study, reporting analyses from 2015 on NCDs in adolescents that confirmed that NCDs are a major public health problem among adolescents globally, and that mental disorders were a large proportion of disability-adjusted life-years (DALYs) in people aged 10–19 years. To our knowledge, a comprehensive and detailed assessment of the burden of both mortality and disability of NCDs and their trends across EU Member States in adolescents aged 10–24 years old has not previously been published.

**Added value of this study**

This study provides a comprehensive description of the mortality and disability burden of NCDs among adolescents aged 10–24 years in EU Member States from 1990 to 2019. We retrieved estimates from Global Burden of Diseases, Injuries, and Risk Factors Study (GBD) 2019, the largest systematic, data-driven, and most recent peer-reviewed assessment of mortality and disability burden by age group, sex, cause, and location. GBD 2019 estimates replace those from previous GBD cycles, as in each iteration the GBD generates revised estimates for the whole time series based on the most updated data and modelling methodology. This study highlights that for the adolescent population mortality has substantially decreased in the past 30 years, and adds to previous studies the important aspect of the rising trend of years of life lost (YLL) rate attributed to mental disorders in this population. It also describes the heavy disability burden attributed to NCDs at the regional and country level in the EU and the concerning increase of years lived with disability (YLDs) due to mental disorders. We also report wide variation in both the mortality and disability burden of NCDs by age group, sex, and location, suggesting opportunities for improvements. We were also able to identify association between the EU Member State level of socioeconomic development and the DALY burden of specific NCDs.

**Implications of all the available evidence**

These findings provide data for evidence-based decision making and highlight priority areas for interventions and investments, such as the importance of promotion of mental wellbeing and prevention of mental disorders, improvements in access to quality mental health services, and investments in dedicated primary and specialist health-care services. The extent of current disability burden of NCDs in adolescents suggests there is a need to scale up high-quality health-care services; establish, develop, and strengthen public health prevention policies, school programmes, specialised training pathways; and ensure that investments address the specific needs of adolescent health. Leadership around these elements could be enhanced by greater access to primary data sources to increase the accuracy of future findings and facilitate timely response to rapid changes in adolescents' health and wellbeing, such as those caused by the COVID-19 pandemic.

## Methods

### Overview

This study adopted the broad age definition for adolescence from 10 to 24 years because it accurately captures the biological, social, and neurocognitive development of this population.^1^ We included the UK in these analyses, as it was still an EU Member State in 2019.

Estimates were retrieved from GBD 2019, which provides a complete set of comparable health estimates for 204 countries, including the 28 EU Member States, for 286 causes of death, 369 causes of disease and injury, and 87 risk factors. GBD 2019 generated estimates using 86 249 sources, and produced estimates of incidence, prevalence, mortality, YLDs, YLLs, DALYs, life expectancy, and health-adjusted life expectancy. To estimate deaths due to different causes, GBD 2019 used vital registration and verbal autopsy data as sources, modelled using the Cause of Death Ensemble model, which used geospatial information from covariates to produce estimates of death for all locations across time (1990–2019). Deaths from vital registration systems coded as unspecified were reassigned using statistical methods.^14^ For most diseases and injuries, data were also modelled using a spatiotemporal Gaussian process regression to allow for smoothing over age, time, and location, and a Bayesian meta-regression modelling tool (DisMod-MR 2.1) that ensured internally consistent estimates among all epidemiological metrics for most causes, by age, sex, location, and year.^14^ Methods for GBD 2019 estimates are described in detail in the capstone papers and appendices.^14^ The GBD 2019 cause list is composed of a four-level hierarchy, with each level comprising mutually exclusive and collectively exhaustive causes. There are 22 level 2 causes, 174 level 3 causes, and 301 level 4 causes (including 131 level 3 causes that are not further disaggregated at level 4). GBD 2019 estimates generated and reported here are in accordance with the Guidelines for Accurate and Transparent Health Estimates Reporting.^15^

### Data analysis

Causes are reported following the GBD hierarchy. To give a general insight of the predominant causes of burden, we analysed all three level 1 causes: communicable, maternal, neonatal, and nutritional conditions; NCDs; and injuries. At level 2 and 3, we exclusively focused on NCD causes (appendix pp 31–33). We excluded self-harm and interpersonal violence from the analysis because the GBD hierarchy includes these in the injuries group (level 1).

Estimates were retrieved for the 28 EU Member States. The analyses covered the period 1990 to 2019, and were stratified by sex and age groups as follows: 10–14 years (younger adolescents), 15–19 years (older adolescents), and 20–24 years (young adults).^6^ Mortality, YLLs, YLDs, and DALYs were all reported as rates per 100 000 population. DALYs are the sum of YLLs and YLDs. YLLs are calculated by subtracting the age at death from the longest possible life expectancy for a person at that age. YLDs are estimated by multiplying the prevalence counts with the disability weight for a given disease or injury. As described in detail in the GBD 2019 capstone paper,^14^ disability weights represent the magnitude of health loss associated with specific health outcomes, and are used to estimate YLDs through a series of severity splits. These metrics were subsequently subdivided by level of causes, specifically total all-cause, level 1, NCD level 2, and cause-specific NCD level 3 (appendix pp 31–33); sex (both sexes, female, and male), and age (10–24 years, 10–14 years, 15–19 years, and 20–24 years); country (28 EU Member States); and trend over time (1990–2019), for which we calculated the percentage change (rate) between 1990 and 2019 in 10–24-year-olds. All estimates generated in GBD 2019 are accompanied by 95% uncertainty intervals (UIs), which represent the 25th and 975th ordered estimates of 1000 draw estimates of the posterior distribution.^14^ We considered estimates to be significantly different by determining whether the 95% UIs overlapped.

Spearman's correlation was used to analyse the social, economic, and demographic diversity of NCD burden between EU Member States by correlating the DALY rates of level 2 NCDs (which comprise both YLDs and YLLs) with each country's score of the Socio-demographic Index (SDI). The SDI is a composite measure of a country's lag-distributed income per capita, average years of schooling, and the fertility rate in females younger than 25 years.^14^, ^16^ The metric is scaled from 0 to 1, where 0 represents the lowest combination of the three indicators and 1 represents the highest. p values of less than 0·05 were set as the threshold of significance. The analysis was done with IBM SPSS Statistics (version 27.0).

### Role of the funding source

The funder of the study had no role in study design, data collection, analysis, and interpretation, or writing of the report.

## Results

### Mortality

In 2019, total all-cause mortality for adolescents aged 10–24 years in the EU was 25·35 (95% UI 24·44–26·27) per 100 000 population (appendix pp 3–4). NCDs accounted for 38·8% (37·4–39·8) of total deaths in this age group (appendix pp 34–35). The leading level 2 cause of death for NCDs in adolescents aged 10–24 years was neoplasms (4·01 [3·62–4·25] per 100 000 population), which accounted for 40·8% (36·8–43·2) of all NCD mortality. The leading level 3 cause of death for NCDs was other malignant neoplasms (1·05 [0·88–1·14) per 100 000 population).

In 2019, NCDs were the leading level 1 cause of death in females of all age categories (52·1% [95% UI 50·3–53·3] for 10–24 years, 63·9% [61·1–65·6] for 10–14 years, 48·0% [45·8–49·5] for 15–19 years, and 50·6% [49·0–51·9] for 20–24 years) and in males aged 10–14 years (54·1% [52·0–56·0]; appendix pp 36–37). Additionally, for both sexes, mortality due to NCDs increased across the three age groups from 5·57 (5·31–5·84) per 100 000 population for 10–14 years to 9·47 (8·96–9·99) per 100 000 population for 15–19 years, and 14·30 (13·67–14·95) per 100 000 population for 20–24 years (appendix pp 34–35). Differences by age and sex in NCD level 2 mortality rates are reported in the appendix (pp 5–6).

**Figure 1:**
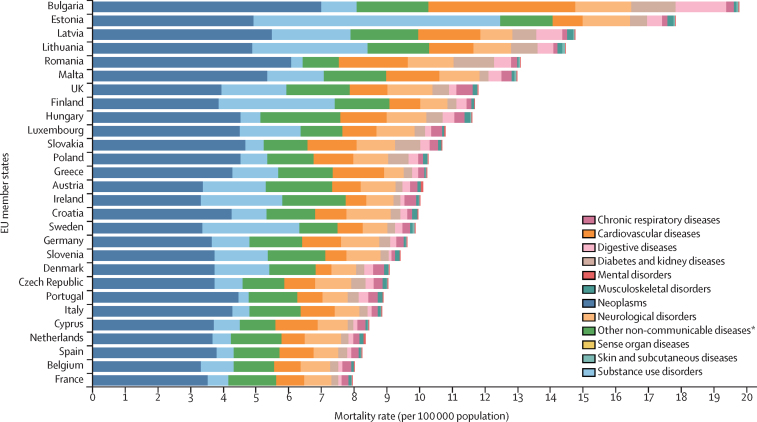
Mortality rate per 100 000 population due to level 2 non-communicable diseases in adolescents aged 10–24 years in both sexes, by country, 2019 *This aggregate cause contains the following level 3 causes: congenital birth defects; urinary diseases; gynaecological diseases; haemoglobinopathies and haemolytic anaemias; endocrine, metabolic, blood, and immune disorders; and oral disorders.

In 2019, the highest mortality rate due to level 2 NCD causes (Bulgaria and Estonia) was more than double the lowest rate (France, Belgium, and Spain; figure 1). Significant differences in the excess mortality rate due to NCDs were observed between eight Member States and the EU overall (compostie estimate): Bulgaria, Estonia, Latvia, Lithuania, Romania, Malta, the UK, and Finland. In all EU Member States, the leading level 2 cause of death was neoplasms, except in Estonia, where it was substance use disorders.

From 1990 to 2019, the mortality rate due to NCDs among adolescents aged 10–24 years significantly declined by 40·4% (95% UI –43·0 to –37·6; table; appendix p 7). For level 2 causes, the highest reduction in mortality rate from NCDs was observed in cardiovascular diseases (–62·00% [–64·37 to –59·63]) and chronic respiratory diseases (–58·81% [–62·27 to –50·24]), whereas the highest increase was in mental disorders (32·36% [2·25 to 66·96]), completely attributed to eating disorders (table; appendix p 7).

**Table:** Mortality and DALY rate per 100 000 population in adolescents aged 10–24 years in EU Member States in 1990 and 2019, and percentage change from 1990 to 2019

		**Mortality rate per 100 000 population**			**DALY rate per 100 000 population**		
		1990	2019	Percentage change, 1990 to 2019	1990	2019	Percentage change, 1990 to 2019
All non-communicable diseases		16·50 (16·20 to 16·72)	9·83 (9·39 to 10·28)	−40·41% (−43·00 to −37·61)	7394·59 (5591·11 to 9580·25)	7015·44 (5181·20 to 9224·35)	−5·13% (−7·55 to −3·21)
Neoplasms		6·32 (6·12 to 6·47)	4·01 (3·62 to 4·25)	−36·61% (−42·27 to −32·78)	461·02 (445·96 to 472·95)	300·48 (271·26 to 321·31)	−34·82% (−40·62 to −30·47)
	Lip and oral cavity cancer	0·04 (0·04 to 0·04)	0·02 (0·02 to 0·03)	−33·96% (−39·54 to −28·14)	2·66 (2·53 to 2·81)	1·78 (1·66 to 1·91)	−33·06% (−38·90 to −27·12)
	Nasopharynx cancer	0·04 (0·04 to 0·04)	0·02 (0·02 to 0·03)	−41·28% (−48·05 to −32·99)	3·07 (2·89 to 3·26)	1·85 (1·65 to 2·06)	−39·93% (−46·80 to −31·53)
	Other pharynx cancer	0·01 (0·01 to 0·01)	0·01 (0·01 to 0·01)	−13·21% (−25·89 to 3·02)	0·46 (0·42 to 0·51)	0·41 (0·36 to 0·47)	−11·41% (−24·25 to 5·13)
	Oesophageal cancer	0·01 (0·01 to 0·01)	0·01 (0·01 to 0·01)	−26·32% (−34·25 to −14·02)	0·68 (0·63 to 0·73)	0·5 (0·46 to 0·56)	−26·12% (−34·05 to −13·75)
	Stomach cancer	0·10 (0·09 to 0·10)	0·03 (0·03 to 0·04)	−65·39% (−68·63 to −62·07)	6·67 (6·35 to 6·99)	2·32 (2·14 to 2·51)	−65·27% (−68·54 to −61·93)
	Colon and rectum cancer	0·13 (0·13 to 0·14)	0·09 (0·08 to 0·10)	−32·71% (−38·30 to −26·95)	9·61 (9·24 to 9·98)	6·57 (6·06 to 7·08)	−31·58% (−37·40 to −25·59)
	Liver cancer	0·08 (0·08 to 0·08)	0·07 (0·06 to 0·07)	−13·45% (−20·87 to −4·91)	5·72 (5·46 to 6·00)	4·96 (4·58 to 5·37)	−13·18% (−20·43 to −4·74)
	Gallbladder and biliary tract cancer	0·01 (0·01 to 0·01)	0·00 (0·00 to 0·00)	−32·91% (−39·98 to −23·07)	0·41 (0·34 to 0·44)	0·27 (0·25 to 0·3)	−32·66% (−39·71 to −22·71)
	Pancreatic cancer	0·03 (0·03 to 0·03)	0·02 (0·02 to 0·03)	−19·65% (−28·73 to −9·72)	2·03 (1·91 to 2·15)	1·63 (1·47 to 1·81)	−19·67% (−28·65 to −9·85)
	Larynx cancer	0·01 (0·01 to 0·01)	0·00 (0·00 to 0·00)	−43·85% (−48·99 to −38·68)	0·59 (0·54 to 0·65)	0·37 (0·32 to 0·41)	−38·03% (−43·52 to −32·58)
	Tracheal, bronchus, and lung cancer	0·13 (0·12 to 0·13)	0·08 (0·07 to 0·09)	−35·97% (−42·35 to −28·10)	8·76 (8·37 to 9·15)	5·62 (5·12 to 6·17)	−35·85% (−42·23 to −27·99)
	Malignant skin melanoma	0·10 (0·07 to 0·12)	0·08 (0·05 to 0·10)	−11·37% (−37·22 to 7·00)	7·07 (5·14 to 8·85)	6·88 (4·44 to 8·14)	−2·71% (−31·93 to 18·35)
	Non-melanoma skin cancer	0·01 (0·01 to 0·01)	0·01 (0·01 to 0·01)	−35·15% (−42·61 to −24·91)	0·66 (0·60 to 0·71)	0·43 (0·39 to 0·47)	−35·05% (−42·48 to −24·87)
	Breast cancer	0·05 (0·05 to 0·06)	0·03 (0·03 to 0·04)	−34·91% (−41·12 to −28·20)	3·90 (3·67 to 4·15)	2·68 (2·43 to 2·95)	−31·32% (−38·15 to −23·90)
	Cervical cancer	0·04 (0·03 to 0·05)	0·02 (0·02 to 0·02)	−53·01% (−59·70 to −44·39)	3·09 (2·38 to 3·4)	1·49 (1·24 to 1·72)	−51·75% (−58·62 to −42·71)
	Uterine cancer	0·00 (0·00 to 0·00)	0·00 (0·00 to 0·00)	−28·44% (−36·45 to −18·94)	0·32 (0·29 to 0·35)	0·24 (0·21 to 0·27)	−24·07% (−33·32 to −13·51)
	Ovarian cancer	0·11 (0·09 to 0·11)	0·07 (0·06 to 0·08)	−34·00% (−45·95 to −8·11)	7·72 (6·57 to 8·28)	5·18 (4·48 to 6·18)	−32·99% (−45·09 to −6·30)
	Prostate cancer	0·01 (0·00 to 0·01)	0·01 (0·00 to 0·01)	−24·99% (−43·26 to 8·07)	0·51 (0·35 to 0·6)	0·43 (0·33 to 0·67)	−16·33% (−37·24 to 21·13)
	Testicular cancer	0·18 (0·17 to 0·20)	0·10 (0·09 to 0·11)	−46·67% (−53·97 to −37·89)	14·00 (13·03 to 15·2)	8·62 (7·48 to 10·13)	−38·43% (−46·69 to −26·98)
	Kidney cancer	0·06 (0·05 to 0·06)	0·05 (0·05 to 0·06)	−3·94% (−13·76 to 7·43)	4·12 (3·89 to 4·35)	3·99 (3·65 to 4·37)	−3·18% (−13·14 to 8·91)
	Bladder cancer	0·01 (0·01 to 0·01)	0·01 (0·01 to 0·01)	−29·00% (−35·00 to −22·42)	0·88 (0·83 to 0·95)	0·66 (0·6 to 0·71)	−25·72% (−31·91 to −18·13)
	Brain and CNS cancer	1·01 (0·86 to 1·23)	0·80 (0·53 to 0·90)	−20·92% (−55·57 to −9·71)	73·56 (62·02 to 89·41)	58·46 (38·55 to 65·75)	−20·53% (−55·31 to −9·09)
	Thyroid cancer	0·02 (0·02 to 0·02)	0·01 (0·01 to 0·01)	−42·24% (−47·52 to −32·32)	1·78 (1·63 to 1·93)	1·13 (1·02 to 1·29)	−36·27% (−42·70 to −25·51)
	Mesothelioma	0·01 (0·00 to 0·01)	0·01 (0·00 to 0·01)	−14·41% (−41·60 to 6·91)	0·39 (0·31 to 0·57)	0·34 (0·28 to 0·38)	−14·42% (−41·55 to 6·88)
	Hodgkin lymphoma	0·30 (0·24 to 0·33)	0·11 (0·10 to 0·15)	−61·87% (−67·17 to −50·97)	22·10 (17·75 to 24·29)	9·43 (8·07 to 12·45)	−57·32% (−63·71 to −44·81)
	Non-Hodgkin lymphoma	0·53 (0·51 to 0·55)	0·33 (0·30 to 0·36)	−38·46% (−43·45 to −31·88)	38·55 (37·07 to 40·05)	23·93 (22·11 to 26·39)	−37·93% (−43·13 to −30·95)
	Multiple myeloma	0·01 (0·00 to 0·01)	0·00 (0·00 to 0·01)	−5·49% (−22·86 to 15·11)	0·35 (0·28 to 0·39)	0·33 (0·27 to 0·38)	−4·81% (−22·21 to 16·04)
	Leukaemia	1·75 (1·69 to 1·81)	0·91 (0·86 to 0·97)	−47·81% (−51·21 to −43·69)	127·46 (123·25 to 131·6)	68·64 (63·95 to 73·69)	−46·14% (−49·95 to −41·92)
	Other neoplasms	0·03 (0·02 to 0·04)	0·03 (0·02 to 0·03)	−3·37% (−32·86 to 19·83)	2·37 (1·80 to 3·21)	2·17 (1·65 to 2·60)	−8·33% (−32·97 to 10·63)
	Other malignant neoplasms	1·52 (1·38 to 1·59)	1·05 (0·88 to 1·14)	−30·96% (−40·75 to −25·24)	111·51 (101·05 to 116·76)	79·17 (66·46 to 86·48)	−29·01% (−38·83 to −22·78)
Cardiovascular diseases		2·96 (2·88 to 3·04)	1·13 (1·06 to 1·19)	−62·00% (−64·37 to −59·63)	249·35 (234·21 to 266·61)	119·45 (105·24 to 135·71)	−52·10% (−55·50 to −48·70)
	Rheumatic heart disease	0·17 (0·16 to 0·17)	0·04 (0·03 to 0·04)	−77·67% (−80–38 to −74·85)	11·90 (11·17 to 12·58)	2·90 (2·57 to 3·24)	−75·63% (−78·69 to −72·57)
	Ischaemic heart disease	0·64 (0·61 to 0·67)	0·20 (0·18 to 0·22)	−69·05% (−71·99 to −65·00)	43·78 (41·62 to 45·99)	13·68 (12·56 to 15·15)	−68·75% (−71·65 to −64·72)
	Stroke	1·15 (1·09 to 1·21)	0·32 (0·29 to 0·35)	−72·45% (−75·16 to −69·34)	106·97 (97·41 to 117·94)	47·55 (39·23 to 56·93)	−55·55% (−60·74 to −50·71)
	Hypertensive heart disease	0·03 (0·02 to 0·03)	0·02 (0·01 to 0·02)	−30·41% (−46·03 to −6·92)	1·86 (1·43 to 2·11)	1·30 (0·97 to 1·55)	−30·14% (−45·26 to −7·21)
	Non-rheumatic valvular heart disease	0·08 (0·07 to 0·08)	0·05 (0·05 to 0·06)	−33·99% (−42·33 to −23·86)	5·4 (4·97 to 5·81)	3·57 (3·24 to 3·97)	−33·95% (−42·22 to −23·87)
	Cardiomyopathy and myocarditis	0·49 (0·43 to 0·57)	0·26 (0·22 to 0·31)	−48·12% (−55·30 to −36·24)	36·54 (31·98 to 41·32)	19·5 (17·13 to 22·87)	−46·62% (−53·59 to −35·22)
	Endocarditis	0·04 (0·04 to 0·07)	0·05 (0·02 to 0·06)	13·52% (−55·14 to 62·70)	3·03 (2·51 to 4·62)	3·42 (1·75 to 4·26)	12·76% (−54·50 to 60·20)
	Aortic aneurysm	0·06 (0·06 to 0·07)	0·04 (0·04 to 0·05)	−34·78% (−42·20 to −25·29)	4·32 (3·96 to 4·66)	2·81 (2·54 to 3·10)	−34·92% (−42·30 to −25·43)
	Other cardiovascular and circulatory diseases	0·30 (0·28 to 0·34)	0·16 (0·14 to 0·20)	−47·64% (−53·26 to −37·53)	35·56 (28·29 to 45·62)	24·72 (18·03 to 34·22)	−30·48% (−38·01 to −23·39)
Chronic respiratory diseases		0·62 (0·56 to 0·65)	0·26 (0·24 to 0·29)	−58·81% (−62·27 to −50·24)	315·34 (216·22 to 447·66)	286·65 (186·47 to 427·65)	−9·10% (−17·36 to 0·36)
	Chronic obstructive pulmonary disease	0·13 (0·12 to 0·14)	0·07 (0·07 to 0·09)	−43·65% (−50·45 to −32·58)	30·64 (25·37 to 35·74)	25·70 (20·54 to 30·95)	−16·13% (−22·76 to −9·10)
	Pneumoconiosis	0·00 (0·00 to 0·00)	0·00 (0·00 to 0·00)	−51·97% (−61·14 to −36·90)	0·20 (0·18 to 0·23)	0·10 (0·08 to 0·12)	−51·16% (−59·49 to −37·99)
	Asthma	0·38 (0·33 to 0·41)	0·1 (0·09 to 0·12)	−73·21% (−75·86 to −66·90)	270·58 (174·63 to 400·11)	242·61 (147·7 to 383·76)	−10·34% (−20·31 to 0·46)
	Interstitial lung disease and pulmonary sarcoidosis	0·04 (0·03 to 0·05)	0·04 (0·03 to 0·05)	5·96% (−30·52 to 32·52)	3·26 (2·52 to 4·04)	3·38 (2·29 to 4·02)	3·59% (−28·84 to 26·10)
	Other chronic respiratory diseases	0·07 (0·04 to 0·07)	0·04 (0·03 to 0·05)	−45·60% (−56·53 to −8·81)	10·66 (8·85 to 12·33)	14·86 (11·67 to 18·08)	39·47% (21·16 to 68·42)
Digestive diseases		0·80 (0·78 to 0·83)	0·39 (0·37 to 0·42)	−51·40% (−54·62 to −48·09)	133·81 (108·81 to 169·8)	103·19 (78·74 to 137·38)	−22·88% (−27·90 to −18·26)
	Cirrhosis and other chronic liver diseases	0·39 (0·37 to 0·41)	0·15 (0·14 to 0·17)	−61·08% (−64·62 to −56·85)	29·86 (28·14 to 31·92)	13·09 (11·57 to 14·94)	−56·15% (−60·29 to −52·20)
	Upper digestive system diseases	0·08 (0·07 to 0·08)	0·02 (0·02 to 0·02)	−73·23% (−76·10 to −69·75)	42·47 (26·44 to 70·23)	36·19 (20·95 to 63·71)	−14·80% (−21·15 to −10·23)
	Appendicitis	0·05 (0·03 to 0·06)	0·01 (0·01 to 0·02)	−69·98% (−74·37 to −52·73)	8·98 (6·47 to 12·55)	7·39 (4·80 to 11·1)	−17·76% (−32·31 to −1·46)
	Paralytic ileus and intestinal obstruction	0·07 (0·06 to 0·08)	0·05 (0·05 to 0·07)	−24·14% (−32·57 to −12·44)	5·61 (4·65 to 6·23)	4·4 (3·72 to 5·18)	−21·50% (−29·31 to −10·85)
	Inguinal femoral and abdominal hernia	0·01 (0·01 to 0·01)	0·00 (0·00 to 0·00)	−69·74% (−73·02 to −63·05)	8·17 (5·03 to 12·55)	6·2 (3·72 to 9·66)	−24·06% (−32·63 to −15·71)
	Inflammatory bowel disease	0·05 (0·04 to 0·06)	0·04 (0·04 to 0·05)	−7·94% (−35·06–6·70)	12·18 (8·87 to 16·06)	13·38 (9·60 to 17·81)	9·84% (−0·88 to 18·84)
	Vascular intestinal disorders	0·02 (0·02 to 0·03)	0·02 (0·01 to 0·02)	−33·51% (−42·43 to −22·53)	1·8 (1·6 to 2·04)	1·26 (1·10 to 1·45)	−29·99% (−38·77 to −19·78)
	Gallbladder and biliary diseases	0·02 (0·01 to 0·02)	0·01 (0·01 to 0·01)	−47·21% (−55·57 to −33·61)	14·57 (8·82 to 22·32)	13·9 (8·40 to 21·85)	−4·63% (−11·67 to 2·30)
	Pancreatitis	0·09 (0·08 to 0·09)	0·05 (0·04 to 0·06)	−42·43% (−50·53 to −31·94)	6·62 (5·94 to 7·35)	4·09 (3·56 to 4·72)	−38·20% (−46·01 to −28·41)
	Other digestive diseases	0·03 (0·02 to 0·04)	0·03 (0·02 to 0·04)	−5·22% (−51·06 to 15·59)	3·54 (2·85 to 4·48)	3·29 (2·36 to 4·06)	−7·31% (−36·65 to 5·40)
Neurological disorders		1·45 (1·41 to 1·50)	1·03 (0·97 to 1·09)	−29·25% (−34·04 to −24·15)	996·17 (321·61 to 2018·69)	985·14 (292·58 to 2019·32)	−1·11% (−10·77 to 5·54)
	Parkinson's disease	0·00 (0·00 to 0·00)	0·00 (0·00 to 0·00)	−31·45% (−43·52 to −16·55)	0·11 (0·09 to 0·13)	0·08 (0·06 to 0·10)	−25·92% (−37·78 to −12·75)
	Idiopathic epilepsy	0·69 (0·66 to 0·73)	0·53 (0·48 to 0·57)	−23·74% (−31·41 to −17·55)	152·76 (104·4 to 220·04)	140·18 (87·22 to 225·90)	−8·23% (−30·47 to 19·70)
	Multiple sclerosis	0·02 (0·02 to 0·03)	0·02 (0·01 to 0·02)	−26·81% (−41·08 to 13·84)	5·61 (3·99 to 7·67)	5·93 (4·22 to 8·18)	5·72% (−4·62 to 20·57)
	Motor neuron disease	0·06 (0·06 to 0·07)	0·06 (0·05 to 0·06)	−12·42% (−19·69 to −5·21)	5·19 (4·89 to 5·55)	4·68 (4·27 to 5·10)	−9·72% (−15·94 to −3·27)
	Headache disorders	..	..	..	761·17 (79·43 to 1792·62)	769·30 (78·52 to 1814·32)	1·07% (−3·41 to 3·70)
	Other neurological disorders	0·67 (0·65 to 0·70)	0·43 (0·40 to 0·46)	−36·56% (−41·28 to −31·01)	71·33 (60·51 to 86·13)	64·96 (47·53 to 93·37)	−8·94% (−25·33 to 14·49)
	Mental disorders	0·01 (0·01 to 0·02)	0·02 (0·01 to 0·02)	32·36%* (2·25 to 66·96)	2008·04 (1420·6 to 2729·61)	2040·59 (1433·96 to 2774·62)	1·62% (−0·61 to 3·87)
	Schizophrenia	..	..	..	63·18 (40·35 to 96·96)	60·24 (38·50 to 92·15)	−4·65% (−11·64 to 1·59)
	Depressive disorders	..	..	..	587·35 (384·87 to 850·24)	569·42 (365·86 to 847·34)	−3·05% (−9·63 to 3·40)
	Bipolar disorder	..	..	..	184·63 (100·79 to 295·7)	187·81 (102·26 to 301·80)	1·72% (−1·93 to 5·58)
	Anxiety disorders	..	..	..	612·59 (398·42 to 900·04)	641·37 (416·23 to 938·58)	4·70% (0·65 to 8·99)
	Eating disorders	0·01 (0·01 to 0·02)	0·02 (0·01 to 0·02)	32·36% (2·25 to 66·96)	130·56 (79·11 to 199·61)	150·46 (90·24 to 230·68)	15·24% (9·65 to 20·43)
	Autism spectrum disorders	..	..	..	89·21 (57·78 to 127·52)	92·27 (60·45 to 132·16)	3·43% (0·55 to 6·17)
	Attention deficit hyperactivity disorder	..	..	..	30·39 (17·04 to 52·32)	32·22 (17·80 to 55·80)	6·03% (0·48 to 11·90)
	Conduct disorder	..	..	..	227·6 (128·22 to 360·70)	234·52 (132·20 to 374·35)	3·04% (0·84 to 5·27)
	Idiopathic developmental intellectual disability	..	..	..	33·67 (15·13 to 57·43)	24·14 (9·31 to 42·55)	−28·29% (−38·34 to −23·60)
	Other mental disorders	..	..	..	48·87 (26·04 to 79·52)	48·14 (25·31 to 77·72)	−1·49% (−6·71 to 3·85)
Substance use disorders		1·30 (1·24 to 1·37)	1·10 (1·01 to 1·21)	−15·28% (−23·50 to −4·57)	492·33 (358·21 to 650·25)	503·94 (361·14 to 665·94)	2·36% (−2·42 to 7·78)
	Alcohol use disorders	0·19 (0·18 to 0·20)	0·14 (0·12 to 0·15)	−27·27% (−37·58 to −17·87)	204·46 (125·09 to 318·64)	187·23 (112·67 to 299·64)	−8·43% (−14·78 to −3·61)
	Drug use disorders	1·11 (1·05 to 1·17)	0·96 (0·88 to 1·07)	−13·22% (−22·66 to −0·90)	287·87 (218·6 to 368·70)	316·72 (234·37 to 412·93)	10·02% (3·03 to 17·91)
Diabetes and kidney diseases		0·46 (0·44 to 0·47)	0·22 (0·21 to 0·24)	−51·23% (−54·66 to −47·98)	69·75 (58·24 to 84·76)	67·92 (51·26 to 88·78)	−2·62% (−13·50 to 8·34)
	Diabetes	0·18 (0·18 to 0·19)	0·10 (0·09 to 0·11)	−46·34% (−50·04 to −42·76)	32·56 (25·46 to 42·02)	42·59 (29·59 to 60·23)	30·83% (14·12 to 44·41)
	Chronic kidney disease	0·27 (0·26 to 0·28)	0·12 (0·11 to 0·13)	−54·13% (−57·79 to −50·04)	36·78 (29·79 to 45·67)	25·21 (18·50 to 33·33)	−31·45% (−38·30 to −25·46)
	Acute glomerulonephritis	0·01 (0·00 to 0·01)	0·00 (0·00 to 0·00)	−74·99% (−79·98 to −68·66)	0·41 (0·35 to 0·49)	0·12 (0·10 to 0·13)	−71·73% (−77·32 to −65·33)
	Skin and subcutaneous diseases	0·02 (0·01 to 0·03)	0·02 (0·01 to 0·03)	−7·51% (−38·94 to 9·70)	731·21 (487·33 to 1051·3)	774·92 (512·68 to 1117·83)	5·98% (4·17 to 7·50)
	Dermatitis	..	..	..	171·24 (94·57 to 280·83)	180·58 (99·20 to 296·53)	5·45% (3·14 to 7·84)
	Psoriasis	..	..	..	100·98 (69·49 to 136·48)	93·69 (64·76 to 126·6)	−7·22% (−10·78 to −3·44)
	Bacterial skin diseases	0·01 (0·01 to 0·02)	0·01 (0·01 to 0·02)	27·07% (−26·87 to 54·15)	6·81 (3·63 to 12·3)	7·21 (3·92 to 12·73)	5·82% (−2·50 to 12·14)
	Scabies	..	..	..	12 (6·39 to 19·95)	9·79 (5·21 to 16·32)	−18·44% (−20·76 to −16·15)
	Fungal skin diseases	..	..	..	26·05 (9·86 to 57·17)	26·39 (9·98 to 57·99)	1·32% (−0·10 to 2·72)
	Viral skin diseases	..	..	..	82·32 (52·54 to 123·64)	85·86 (55·49 to 128·88)	4·31% (2·61 to 6·07)
	Acne vulgaris	..	..	..	253·02 (149·86 to 400·58)	293·19 (174·43 to 464·77)	15·88% (13·70 to 18·20)
	Alopecia areata	..	..	..	7·05 (4·44 to 10·68)	7·01 (4·39 to 10·61)	−0·49% (−5·86 to 5·53)
	Pruritus	..	..	..	5·16 (2·34 to 9·82)	5·27 (2·4 to 10·08)	2·21% (−0·84 to 5·66)
	Urticaria	..	..	..	36·57 (22·68 to 55·92)	34·64 (21·53 to 53·01)	−5·28% (−8·54 to −1·74)
	Decubitus ulcer	0·00 (0·00 to 0·00)	0·00 (0·00 to 0·00)	−48·30% (−67·63 to −29·59)	0·52 (0·36 to 0·73)	0·51 (0·34 to 0·71)	−3·19% (−11·98 to 5·52)
	Other skin and subcutaneous diseases	0·01 (0·00 to 0·01)	0·00 (0·00 to 0·00)	−57·16% (−64·92 to −44·05)	29·49 (14·21 to 54·82)	30·78 (14·58 to 57·44)	4·36% (2·36 to 6·00)
	Sense organ diseases	..	..	..	161·30 (104·92 to 232·98)	150·24 (98·94 to 216·39)	−6·86% (−10·66 to −3·53)
	Blindness and vision loss	..	..	..	57·93 (35·68 to 88·02)	55·65 (33·94 to 85·36)	−3·95% (−7·47 to −1·02)
	Age-related and other hearing loss	..	..	..	89·07 (53·43 to 131·61)	79·48 (48·09 to 118·58)	−10·77% (−16·00 to −5·55)
	Other sense organ diseases	..	..	..	14·30 (7·93 to 23·33)	15·12 (8·43 to 24·67)	5·73% (0·53 to 11·81)
Musculoskeletal disorders		0·14 (0·09 to 0·19)	0·09 (0·07 to 0·14)	−33·92% (−42·01 to −11·15)	975·56 (670·99 to 1372·96)	974·22 (674 to 1377·19)	−0·14% (−2·31 to 2·10)
	Rheumatoid arthritis	0·01 (0·01 to 0·02)	0·01 (0·01 to 0·01)	−55·60% (−63·33 to −38·51)	9·19 (5·98 to 13·52)	9·80 (6·18 to 14·89)	6·59% (−3·50 to 15·33)
	Low back pain	..	..	..	678·57 (439·08 to 992·73)	634·68 (410·14 to 938·51)	−6·47% (−8·88 to −3·94)
	Neck pain	..	..	..	157·22 (89·41 to 267·77)	172·02 (97·60 to 290·28)	9·41% (6·44 to 12·68)
	Gout	..	..	..	0·35 (0·15 to 0·68)	0·38 (0·16 to 0·71)	6·34% (2·60 to 13·07)
	Other musculoskeletal disorders	0·12 (0·08 to 0·17)	0·08 (0·07 to 0·13)	−31·37% (−39·81 to −7·66)	130·22 (76·37 to 204·72)	157·34 (91·23 to 246·73)	20·82% (14·31 to 29·20)
Other non-communicable diseases		2·41 (2·11 to 2·62)	1·57 (1·42 to 1·85)	−34·70% (−39·50 to −21·57)	800·72 (594·95 to 1076·26)	708·7 (515·60 to 964·73)	−11·49% (−14·60 to −8·10)
	Congenital birth defects	1·53 (1·24 to 1·70)	0·87 (0·71 to 1·10)	−42·91% (−48·73 to −21·92)	191·85 (161·2 to 226·95)	143·82 (117·64 to 175·65)	−25·03% (−30·85 to −12·47)
	Urinary diseases and male infertility	0·09 (0·08 to 0·10)	0·05 (0·05 to 0·06)	−45·11% (−49·24 to −35·04)	14·45 (11·1 to 19·37)	11·37 (7·99 to 16·99)	−21·31% (−29·82 to −9·92)
	Gynaecological diseases	0·00 (0·00 to 0·01)	0·00 (0·00 to 0·00)	−65·55% (−71·31 to −43·29)	277·7 (180·92 to 410·57)	272·17 (176·44 to 402·67)	−1·99% (−4·86 to 1·22)
	Haemoglobinopathies and haemolytic anaemias	0·22 (0·22 to 0·23)	0·08 (0·08 to 0·09)	−63·33% (−66·84 to −59·22)	34·79 (27·39 to 45·83)	13·88 (10·67 to 18·53)	−60·11% (−64·82 to −54·56)
	Endocrine, metabolic, blood, and immune disorders	0·56 (0·44 to 0·66)	0·57 (0·49 to 0·77)	1·17% (−7·60 to 24·29)	183·24 (124·57 to 260·31)	176·43 (122·63 to 245·60)	−3·72% (−8·14 to 2·88)
	Oral disorders	..	..	..	98·69 (53·76 to 163·39)	91·03 (50·27 to 150·71)	−7·76% (−10·76 to −4·85)

Data in parentheses are 95% uncertainty intervals.

* Note that deaths were only attributed to eating disorders.

### Years of life lost

In 2019, all-cause YLL rates per 100 000 population in the EU were 1758·00 (95% UI 1694·58–1822·04) among adolescents aged 10–24 years (appendix p 8). Among NCDs, the leading level 2 cause of YLLs was neoplasms (281·78 [254·25–298·92] per 100 000 population), whereas at level 3, the three leading causes were other malignant neoplasms (74·01 [62·15–80·22] per 100 000 population), drug use disorders (65·02 [59·34–72·26] per 100 000 population), and leukaemia (64·64 [60·62–69·01] per 100 000 population; appendix pp 38–39).

**Figure 2:**
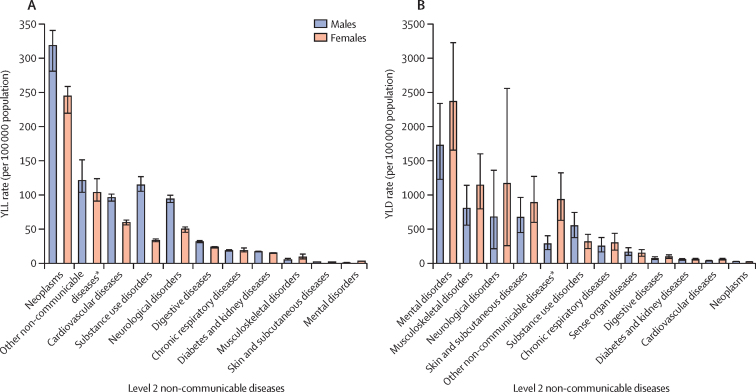
YLL (A) and YLD (B) rates per 100 000 population due to level 2 non-communicable diseases in adolescents aged 10–24 years in EU Member States, by sex, 2019 YLDs=years lived with disability. YLLs=years of life lost. *This aggregate cause contains the following level 3 causes: congenital birth defects; urinary diseases; gynaecological diseases; haemoglobinopathies and haemolytic anaemias; endocrine, metabolic, blood, and immune disorders; and oral disorders.

In 2019, all-cause YLL rates per 100 000 population were significantly higher in males (2415·92 [95% UI 2321·65–2509·04]) than in females (1060·34 [1024·01–1099·59]), with the largest differences in those aged 20–24 years, with a male-to-female ratio of 2·8:1 (appendix p 8). NCDs were the leading cause of YLLs among females in all three age groups (63·9% [61·1–65·6] for 10–14 years, 48·0% [45·8–49·5] for 15–19 years, and 50·6% [49·0–51·9] for 20–24 years) and in the youngest males (54·1% [52·0–56·0] for 10–14 years; appendix pp 36–37). Sex and age-group differences for NCD level 2 causes of YLL are reported in figure 2A and the appendix (pp 9–10). Significant sex differences in YLL rates per 100 000 population in adolescents aged 10–24 years were apparent for several NCD level 3 causes, most notably for drug use disorders (98·38 [88·76–111·23] for males *vs* 29·64 [27·09–32·33] for females), other neurological disorders (45·93 [42·44–49·72] *vs* 13·1 [12·0–14·4]), alcohol use disorders (15·25 [13·04–17·02] *vs* 3·16 [2·82–3·50]; appendix p 11).

In 2019, the five countries with the largest burden of YLL rate per 100 000 population for NCDs in adolescents aged 10–24 years were Bulgaria (1381·32 [95% UI 1102·73–1714·70]), Estonia (1230·34 [1004·84–1511·39]), Latvia (1028·11 [870·39–1237·01]), Lithuania (1004·40 [842·92–1192·36]), and Romania (918·53 [779·76–1079·00]; appendix pp 12–13). The highest YLL rate (in Bulgaria) was double the lowest (in France). Neoplasms were the leading cause of YLL rate in all Member States in adolescents aged 10–24 years, except for Estonia, where it was substance use disorders (appendix pp 12–13). In comparison to the EU overall, eight Member States had significantly higher YLL rates due to NCDs (Bulgaria, Estonia, Latvia, Lithuania, Malta, Romania, the UK, and Finland), whereas five had lower rates (Italy, the Netherlands, Spain, Belgium, and France).

Among adolescents aged 10–24 years, YLL rates due to NCDs decreased by 40·56% (95% UI –43·16 to –37·74) from 1990 to 2019 (appendix pp 14, 38–39). The only NCD level 2 cause of YLLs that increased was mental disorders, albeit not significantly, from 0·93 (0·80–1·10) in 1990 to 1·23 (1·00–1·50) in 2019 (change 32·18% [1·67–66·49]), and for which YLLs were only attributed to eating disorders (appendix pp 38–39).

### Years lived with disability

The all-cause YLD rate across the EU in 2019 was 7322·85 (95% UI 5268·10–9748·95) per 100 000 population among adolescents aged 10–24 years and increased by age group (appendix p 15). The leading level 1 causes of YLDs in adolescents aged 10–24 years were NCDs (6328·51 [4489·66–8533·25] per 100 000 population), which overall constituted 86·4% (83·5–88·8) of all YLDs (appendix p 34). The leading NCD level 2 causes of YLDs were mental disorders (2039·36 [1432·56–2773·47] per 100 000 population), and the top three level 3 causes of YLDs were headache disorders (769·30 [78·52–1814·32] per 100 000 population), anxiety disorders (641·37 [416·23–938·51] per 100 000 population), and low back pain (634·68 [410·14–938·51] per 100 000 population; appendix pp 40–42).

Among adolescents aged 10–24 years, all-cause YLD rates per 100 000 population in 2019 were higher in females (8383·40 [95% UI 6003·72–11 292·01]) than in males (6322·73 [4586·09–8354·55]; appendix p 15). NCDs were the leading level 1 cause of YLDs, with the greatest burden in individuals aged 20–24 years (7813·15 [5546·85–10 334·38] per 100 000 population) (appendix pp 34–35). For all three age subgroups, mental disorders were the leading level 2 cause of YLDs in the EU overall (appendix pp 16–17). Differences by age group and sex in level 2 causes are reported in figure 2B and the appendix (pp 16–17). For level 3 causes, significant differences in YLD rates per 100 000 population between females and males aged 10–24 years were observed for eating disorders (236·68 [141·97–365·30] for females *vs* 66·76 [38·98–104·81] for males) and autism spectrum disorders (32·77 [21·26–47·87] *vs* 148·39 [97·20–211·37]; appendix p 18).

In 2019, among adolescents aged 10–24 years, country NCD YLD rates per 100 000 population ranged from 4593·38 (95% UI 3234·52–6166·79) in Romania to 7018·56 (4996·04–9428·21) in Portugal (appendix pp 19–20). However, no significant differences were observed between individual Member States and the EU overall. Mental disorders were the leading cause of YLDs in all countries.

Among adolescents aged 10–24 years, there was no change in all-cause YLD rates from 1990 (7372·49 [95% UI 5298·54–9758·09] per 100 000 population) to 2019 (7322·85 [5268·10–9748·95] per 100 000 population; appendix p 15), although a slight increase in YLDs due to NCDs was observed (1·44% [0·09–2·79]; appendix pp 21, 40–42). Within NCD level 2 causes, from 1990 to 2019, the greatest increase in YLD rates was observed for diabetes and kidney diseases (37·8% (25·1–51·5), mainly due to an increase in level 3 cause diabetes (80·5% [69·5–90·4]; appendix pp 40–42).

### Disability-adjusted life-years

In 2019, the all-cause rate of DALYs per 100 000 population was 9080·85 (95% UI 7024·87–11 479·33) among adolescents aged 10–24 years (appendix p 22). NCDs accounted for 77·1% (73·5–80·5) of DALYs among adolescents aged 10–24 years in the EU (appendix pp 34–35). Mental disorders (2040·59 [1433·96–2774·62] per 100 000 population) were the leading level 2 cause (table), accounting for 29·1% (20·4 –39·6) of the overall NCD DALY rate. Headache disorders (769·30 [78·52–1814·32] per 100 000 population), anxiety disorders (641·37 [416·23–938·58] per 100 000 population), and low back pain (634·68 [410·14–938·51] per 100 000 population) were the top three level 3 causes.

The total all-cause DALY rate per 100 000 population in 2019 increased with age and was higher in females than males in all age groups (appendix p 22). NCDs were the leading causes of DALYs in all age groups and in both sexes (appendix pp 34–37). Differences between sexes were larger in the age groups of 15–19 years and 20–24 years. For adolescents aged 10–24 years, sex differences were significant for level 2 causes of substance use disorders, neurological disorders, and other NCDs (appendix pp 23–24), and at level 3 they were significant for drug use disorders (221·64 [156·16–299·71] per 100 000 population for females *vs* 406·38 [304·06–522·44] per 100 000 population for males) and eating disorders (239·16 [143·64–367·68] per 100 000 population *vs* 66·82 [39·04–104·88] per 100 000 population; appendix p 25).

**Figure 3:**
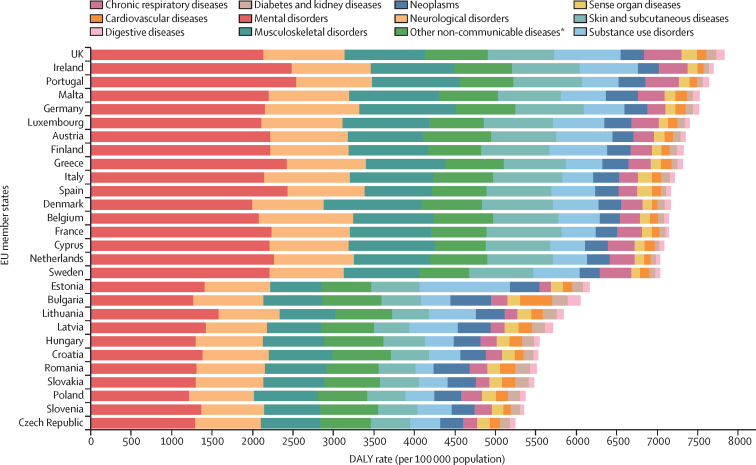
DALY rate per 100 000 population due to level 2 non-communicable diseases in adolescents aged 10–24 years in both sexes, by country, 2019 DALY=disability-adjusted life-year. *This aggregate cause contains the following level 3 causes: congenital birth defects; urinary diseases; gynaecological diseases; haemoglobinopathies and haemolytic anaemias; endocrine, metabolic, blood, and immune disorders; oral disorders.

In 2019, DALY rates per 100 000 population due to NCDs in adolescents aged 10–24 years ranged from 5248·13 [95% UI 3890·06–6787·39]) in the Czech Republic to 7828·83 [5815·44–10 207·98]) in the UK (figure 3). In all EU Member States, mental disorders were the leading level 2 cause of DALYs from NCDs (figure 3), accounting for more than 22·5% (15·8–30·6) of the DALY burden. Significantly higher rate differences between Member States and the EU overall were observed at level 2 causes for the following diseases and countries: cardiovascular diseases (Bulgaria, Romania, and Latvia), digestive diseases (Bulgaria, Romania, and Lithuania), diabetes and kidney diseases (Bulgaria), neoplasms (Bulgaria, Romania, Latvia, and Malta), and substance use disorders (Estonia; appendix pp 26–28).

In the 30-year period, the DALY rate due to NCDs across the EU decreased by 5·1% (95% UI –7·6 to –3·2; table; appendix p 29). Among level 2 NCD causes, there was a significant change in the DALY rate from 1990 to 2019 for cardiovascular diseases, which decreased by 52·10% (–55·50 to –48·70), and neoplasms, which decreased by 34·82% (–40·62 to –30·47; table).

### Correlation between SDI and NCD DALY rates

In 2019, the SDI ranged from 0·74 (Portugal) to 0·90 (Germany and Luxembourg; appendix p 43). Moderate rank correlations of higher developmental index and higher DALY rates of substance use disorders (*r*_s_=0·58, p=0·0012) and skin and subcutaneous diseases (*r*_s_=0·45, p=0·017) and of lower developmental index and higher DALY rates of cardiovascular diseases (*r*_s_=–0·46, p=0·015), neoplasms (*r*_s_=–0·57, p=0·0015), and sense organ diseases (*r*_s_=–0·61, p=0·0005) were observed (appendix pp 30, 44).

## Discussion

This study presents the first systematic analysis of the NCD burden among adolescents in the EU Member States using GBD 2019 estimates. It found that the burden of NCD mortality and disability increases between the age groups 10–14 and 20–24 years. Despite substantial decreases in mortality over the past three decades, disability has remained mostly unchanged over this time, and the rising trend of YLLs attributed to mental disorders and their YLD burden are concerning. In line with previous evidence reporting that sex differences tend to increase with age,^4^, ^17^ our findings show that sex differences are wider in young adults. Furthermore, although males have a higher mortality and a major burden attributed to substance use disorders, females present a higher disability burden, particularly attributable to mental disorders, with an emerging mortality burden of eating disorders. Substantial variations of the NCD burden by country were also found. Mortality and YLLs from NCDs predominated in eastern European countries (Bulgaria, Estonia, Latvia, Lithuania, and Romania), in comparison to greater prominence from disability in western European countries (the UK, Portugal, Ireland, Germany, and Luxembourg).

This study highlights the need to scale up wide-ranging interventions to address the challenge of NCDs in adolescents in EU Member States, particularly aiming in reducing the disability burden of these diseases. These interventions comprise holistic multilevel public health approaches,^9^ including evidence-based preventive interventions, investments in dedicated primary and specialist health-care services including specialist training in adolescent medicine,^18^ and health-promoting school programmes.^19^ Effective interventions should also consider structural and proximal social and environmental determinants of health, such as improving access to education and employment, as well as commercial determinants that shape ill health.^20^, ^21^

Within the EU, despite the fifth European Youth Goal^10^ to promote social inclusion of all young people, to achieve better mental health and wellbeing and end stigmatisation of mental health issues, mental disorders were the major contributors of the NCD burden in all EU Member States and in adolescents. Previous studies have reported mental disorders as leading causes of disability among adolescents,^8^ and that the onset of the first mental disorder emerges in a third of individuals before the age of 14 years, in almost half by 18 years, and nearly two-thirds before 25 years.^22^ Yet only 20–40% of adolescents with mental health problems are diagnosed by health services and only 25% receive appropriate treatment.^23^ This problem is compounded by low help-seeking behaviour^24^ and is probably exacerbated by barriers to accessing mental health services,^25^ such as stigma, service cost, the absence of health services, or the requirement for parental consent. Gender inequalities in health primarily emerge during adolescence,^26^ and this difference reinforces the importance of prioritising adolescents of all genders as an age group for targeted gender-sensitive health policies, indicators, and programmes. Examples include mainstreaming gender in health service delivery and access, in medical research, in health planning processes, and in the training of health-care professionals, which would be expected to enhance the effectiveness of actions to address the burden of NCDs. Moreover, the correlation between DALY rates and SDI of each EU Member State also confirms the need to address underlying determinants of health and suggests that country-specific approaches are needed.^27^ For example, Bulgaria and Romania, which have the lowest expenditure on health in the EU,^28^ would benefit from greater investments to improve access to and quality of health services, including for adolescents,^29^ national health system monitoring or quality assurance systems, and prevention,^28^ whereas Estonia, with the highest burden of substance use disorder in adolescents, would benefit from increased drug-related expenditure, including for tackling gaps in data collection, which accounted for only 0·02% of gross domestic product in 2011, well below the EU average.^30^

Understanding and responding to these barriers is particularly urgent given the impact of the COVID-19 pandemic on adolescent health and health-related quality of life.^12^ In this context, besides mental disorders,^31^ there are also concerns about the impact of the COVID-19 pandemic on reducing access to health services for other NCDs, such as cancer. Despite significant improvements in mortality reduction due to neoplasms in EU Member States, these gains might be jeopardised by the disruptions to cancer care services faced during COVID-19 pandemic.^32^ Additionally, considering the rising trend of disability due to diabetes, the alarming increase of type 2 diabetes in adolescents,^33^, ^34^ and long-term effects of COVID-19 on obesity and type 1 diabetes, reasonable prevention strategies and health system responses should be prioritised.

GBD 2019 has some key limitations, such as availability of primary data, the uncertainty for some estimates represented by a wide 95% UIs, as well as in the determination and classification of some non-fatal disorders. Further details in GBD 2019 limits are described elsewhere.^14^ Our analysis has several limitations related to variation in the availability and quality of primary data for adolescent health, including paucity of data for some age groups (especially 10–14 years), for many health outcomes during adolescence,^35^ and some EU Member States, particularly in central and eastern Europe. These estimates of disease burden are surrounded by considerable uncertainties and different data availability between countries can generate difficulties in the interpretation of comparisons. Additionally, not all sources of uncertainty could be routinely captured in either the epidemiological or cause-of-death modelling processes. It is important to note that both disability and mortality rates of mental disorders will have been underestimated as self-harm and interpersonal and sexual violence were excluded from the analysis (due to GBD 2019 grouping these within injures group). In addition, mental disorders and self-harm are often underdiagnosed or misdiagnosed, among other reasons, due to implicit stigma affecting both patients and clinicians. Reporting bias might be relevant as well in stigmatised disorders such as mental disorders and substance use disorders. Finally, although we used SDI to describe socioeconomic differences among countries, other indicators might be more relevant for adolescents, and further disaggregation, such as ethnicity, could provide additional information.

Despite two decades of attention to adolescent-friendly health services that consider the context of adolescent's biological and social development,^36^ these data on NCDs are consistent with concerns that the quality of health care currently provided to adolescents in the EU is less than optimal.^18^ Addressing NCDs in adolescents is complex, as adolescence is a period in which both NCDs begin and many NCD risk behaviours start, with the related burden of diseases becoming visible only in adulthood, as it is estimated that about 70% of premature deaths occurring during adulthood result from health-related behaviours initiated in childhood and adolescence.^6^ Although various plans and strategies are in place at the regional level (ie, the EU level), with some evidence of national plans, the high disability burden due to adolescent NCDs indicates inadequate implementation of key policies and severe underfunding in many countries. NCDs in adolescents have been largely ignored in global targets for the UN Sustainable Development Goals (SDGs).^37^ Yet responses are urgently needed as these data on adolescents in 2019 will be reflected in national adult targets for NCDs within the 2030 UN SDGs.

## Data sharing

To download the data used in these analyses, please visit the Global Health Data Exchange at http://ghdx.healthdata.org/gbd-results-tool.

## Declaration of interests

We declare no competing interests.
