# Supplementary material for: Burden of non-communicable diseases among adolescents aged 10–24 years in the EU, 1990–2019: a systematic analysis of the Global Burden of Diseases Study 2019
Source: Lancet Child Adolesc Health. 2022 Jun;6(6):367–83. doi: 10.1016/S2352-4642(22)00073-6 (PMC9090900; doi:10.1016/S2352-4642(22)00073-6)
Supplement: Supplementary appendix [file mmc1.pdf]

THE LANCET
Child & Adolescent Health

Supplementary appendix

This appendix formed part of the original submission and has been peer reviewed. We post it as supplied by the authors.

Supplement to: Armocida B, Monasta L, Sawyer S, et al. Burden of non-communicable diseases among adolescents aged 10–24 years in the EU, 1990–2019: a systematic analysis of the Global Burden of Diseases Study 2019. *Lancet Child Adolesc Health* 2022; published online March 24. https://doi.org/10.1016/S2352-4642(22)00073-6.

Supplement to: Burden of non-communicable diseases among adolescents aged 10-24 years in the European Union, 1990-2019: a systematic analysis of the Global Burden of Diseases Study 2019.

Table of content

Appendix Figure 1. All-cause mortality rates per 100,000 population, in people aged 10–24 years from 1990 to 2019 by sex......3

Appendix Figure 2. All-cause mortality rates per 100,000 population, in people aged 10–24 years from 1990 to 2019 by sex and age group......4

Appendix Figure 3. Mortality rates per 100,000 population, level 2 NCDs causes, by age group and sex, in the European Union, in 2019......5

Appendix Figure 4. Mortality rates per 100,000 population, NCDs level 2 causes, 10-24 years old, both sexes, in the European Union, trend from 1990-2019......7

Appendix Figure 5. All-cause YLL rates per 100,000 population, in people aged 10–24 years from 1990 to 2019 by sex and age group......8

Appendix Figure 6. YLL rates per 100,000 population, level 2 NCDs causes, in the European Union, in 2019, by age group and sex......9

Appendix Figure 7. YLL rates per 100,000 population, first 20 level 3 NCDs causes, in people 10-24 years old, in the European Union, 2019, by sex......11

Appendix Figure 8. YLL rates per 100,000 population (Panel A), and in percentage (Panel B), NCDs level 2, both sexes, 10-24 years old, 2019, by location......12

Appendix Figure 9. Level 2 NCD causes, in the European Union, in people aged 10-24 years, both sexes, from 1990 to 2019: YLLs rate per 100,000 population......14

Appendix Figure 10. All-cause YLD rates per 100,000 population, in people aged 10–24 years from 1990 to 2019 by sex and age group......15

Appendix Figure 11. YLD rates per 100,000 population, level 2 NCDs causes, European Union, 2019, by age group and sex......16

Appendix Figure 12. YLD rates per 100,000 population, first 20 level 3 NCDs causes, in people 10-24 years old, in the European Union, 2019, by sex......18

Appendix Figure 13. YLD rates per 100,000 population (Panel A), and in percentage (Panel B), NCDs level 2, both sexes, 10-24 years old, 2019, by location......19

Appendix Figure 14. Level 2 NCD causes, in the European Union, in people aged 10-24 years, both sexes, from 1990 to 2019: YLDs rate per 100,000 population......21

Appendix figure 15. All-cause DALY rates per 100,000 population, in people aged 10–24 years from 1990 to 2019 by sex and age group......22

Appendix Figure 16. DALY rates per 100,000 population, level 2 NCDs causes, European Union, 2019, by age group and sex......23

Appendix Figure 17. DALY rates per 100,000 population, first 20 level 3 NCDs causes, in people 10-24 years old, in the European Union, 2019, by sex......25

Appendix Figure 18. DALY rates per 100,000 population, among 10-24 years old adolescents, both sexes, 2019, level 2 NCDs 26

Appendix Figure 19. DALY rates per 100,000 population, NCDs level 2 causes, 10-24 years old, both sexes, in the European Union, trend from 1990-2019 29

Appendix Figure 20. Relation between DALY rates per 100,000 population of five NCDs and Socio Demographic Index (SDI), in 28 EU Member States, year 2019, both sexes, age 10-24 30

Appendix table 1. Global Burden of Disease 2019 Cause Hierarchy 31

Appendix table 2. Level 1 mortality, YLL, YLD and DALY rates per 100,000 population with 95% UI and respective percentage among age groups, both sexes, in the European Union, year 2019 34

Appendix table 3. Level 1 mortality, YLL, YLD and DALY rates per 100,000 population with 95% UI and respective percentage among age groups, by sex, in the European Union, year 2019 36

Appendix table 4. Adolescents (10-24 years old) YLL rates in 1990 and 2019 and percentage change from 1990 to 2019 38

Appendix table 5. Adolescents (10-24 years old) YLD rates in 1990 and 2019 and percentage change from 1990 to 2019 40

Appendix table 6. Socio-demographic index in 2019 for the 28 European Union Member States 43

Appendix table 7. Spearman rank-correlation coefficients and relative P-values for the association between DALY rates of level 2 NCDs and SDI in the 28 European Union Member States, both sexes, age 10-24, year 2019 44

Contributions 45

Appendix Figure 1. All-cause mortality rates per 100,000 population, in people aged 10–24 years from 1990 to 2019 by sex

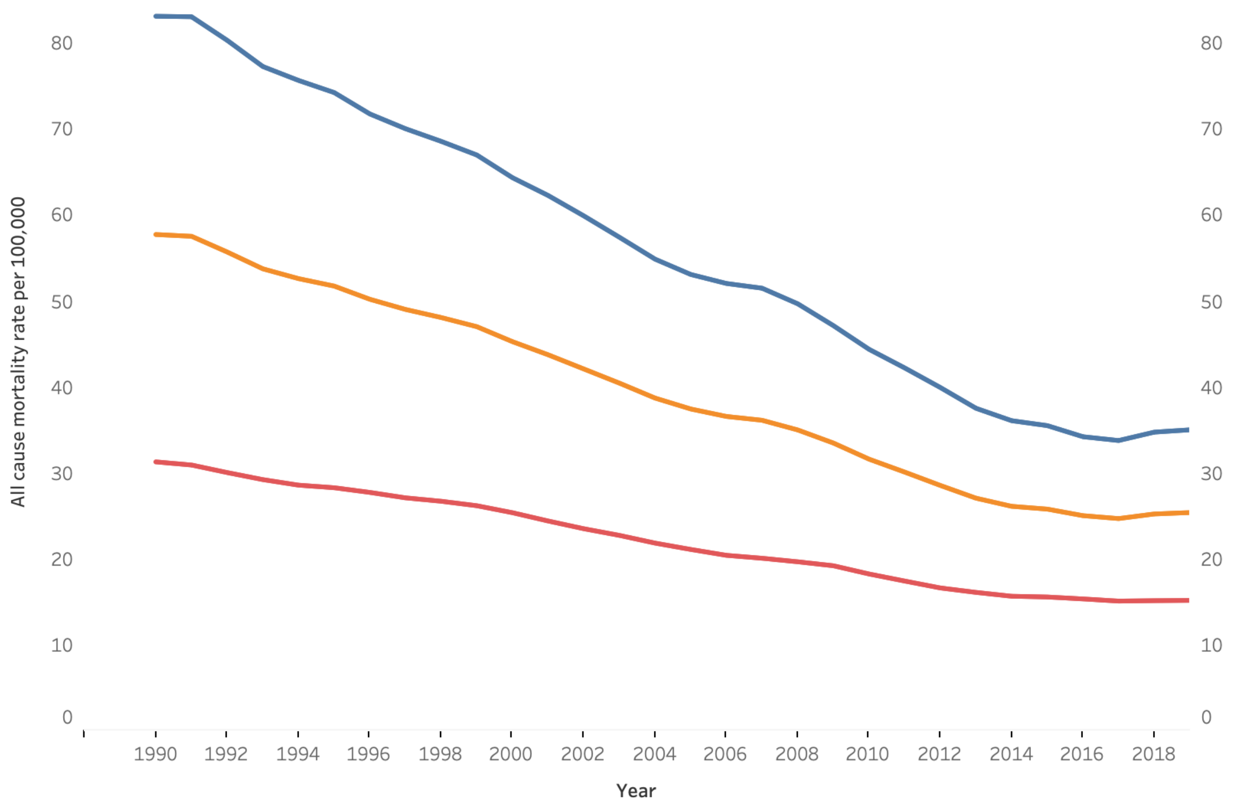

Sex
- Both sexes
- Females
- Males

Appendix Figure 2. All-cause mortality rates per 100,000 population, in people aged 10–24 years from 1990 to 2019 by sex and age group

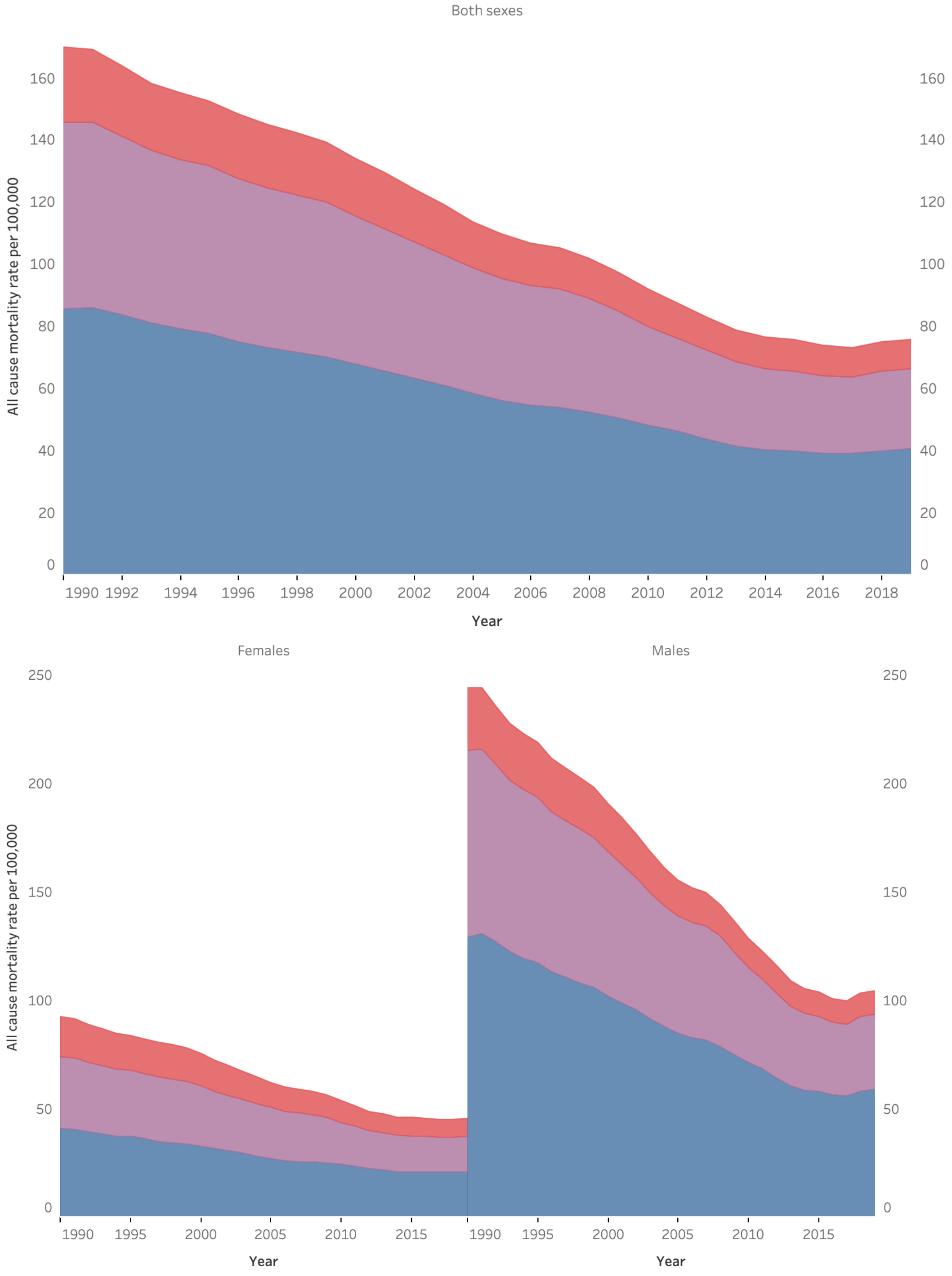

Age groups (years)
10-14
15-19
20-24

Appendix Figure 3. Mortality rates per 100,000 population, level 2 NCDs causes, by age group and sex, in the European Union, in 2019

Panel A

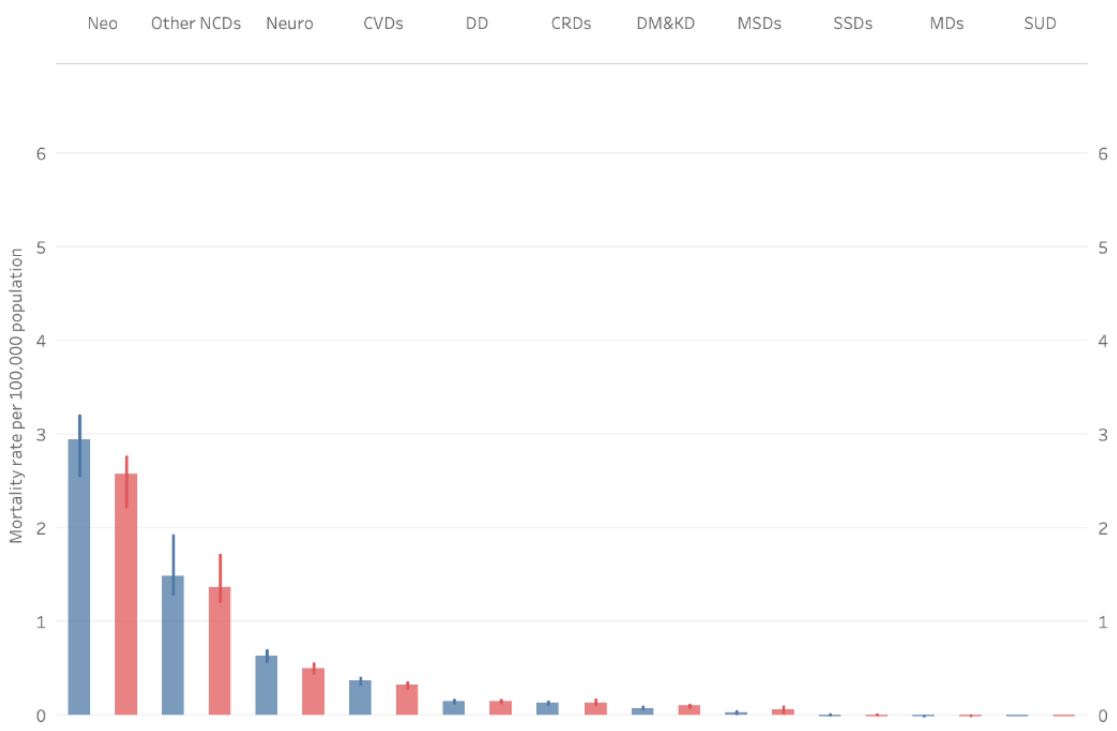

Panel B

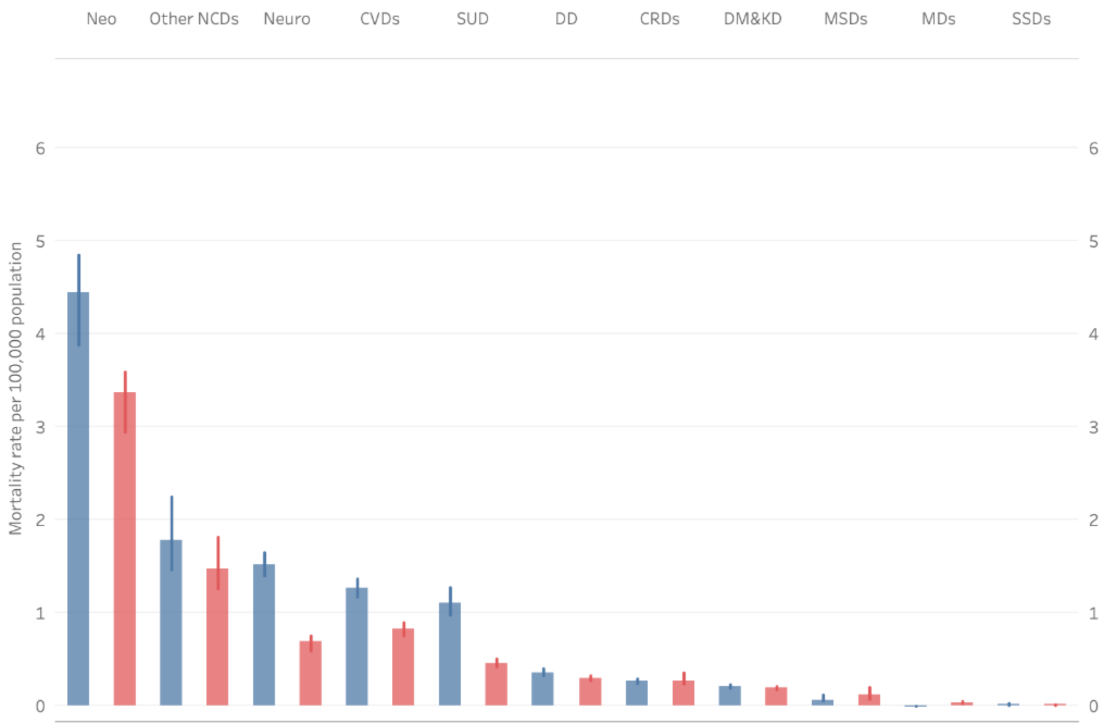

Panel C

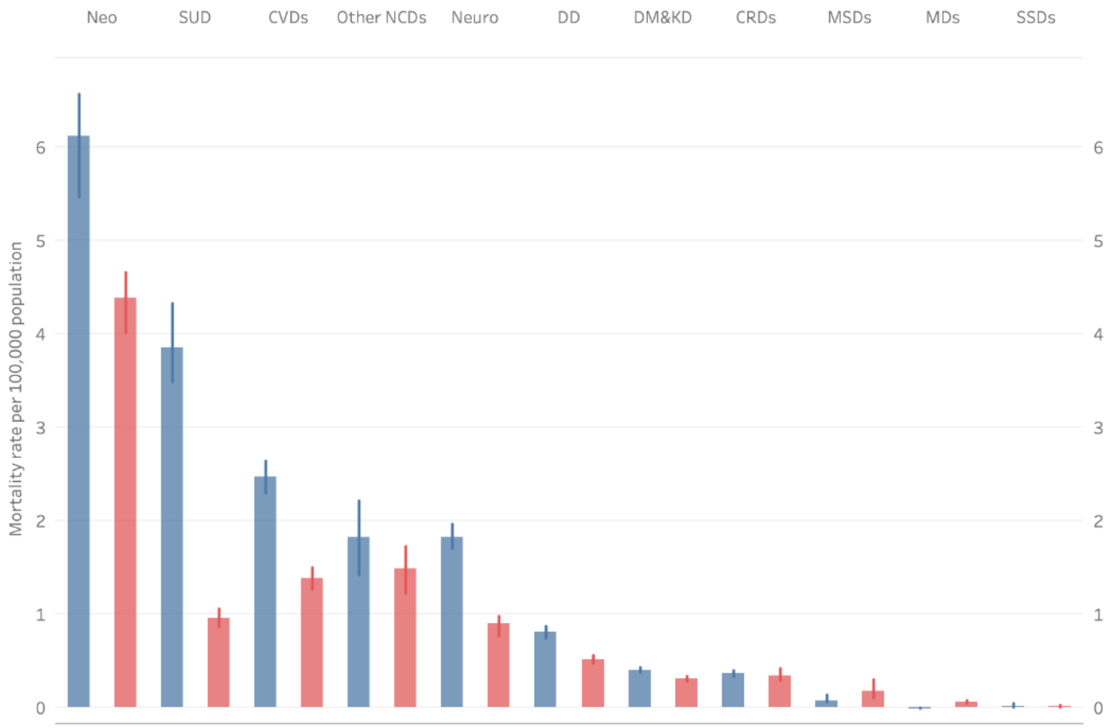

Sex
Males
Females

Panel A: 10-14 years old
Panel B: 15-19 years old
Panel C: 20-24 years old

Abbreviations: CRDs=Chronic respiratory diseases; CVDs=cardiovascular diseases; DD=Digestive diseases; DM&KD=Diabetes and kidney diseases; MDs=Mental health disorders; MSDs=Musculoskeletal disorders; Neo=Neoplasms; Neuro= Neurological disorders; Other NCDs=Other non-communicable diseases; SSDs=Skin and subcutaneous diseases; SUD=Substance use disorders.

Appendix Figure 4. Mortality rates per 100,000 population, NCDs level 2 causes, 10-24 years old, both sexes, in the European Union, trend from 1990-2019

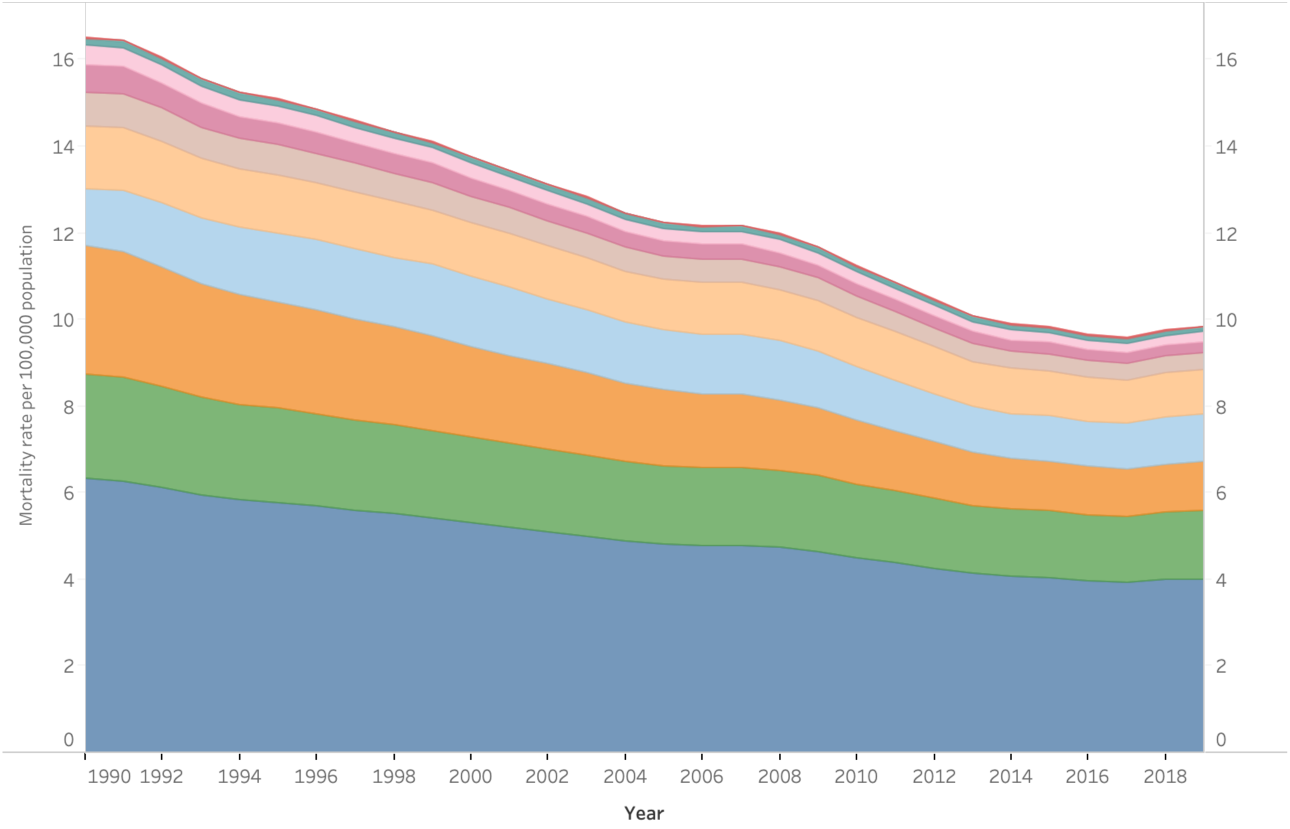

Legend:
- Chronic respiratory diseases
- CVDs
- Diabetes and kidney diseases
- Digestive diseases
- Mental health disorders
- Musculoskeletal disorders
- Neoplasms
- Neurological disorders
- Other NCDs*
- Sense organ diseases
- Skin and subcutaneous diseases
- Substance use disorders

Abbreviations: NCDs=non-communicable diseases; CVDs=cardiovascular diseases

*Note: This aggregate cause contains the following Level 3 causes: congenital birth defects; urinary diseases; gynaecological diseases; haemoglobinopathies and haemolytic anaemias; endocrine, metabolic, blood, and immune disorders; oral disorders.

Appendix Figure 5. All-cause YLL rates per 100,000 population, in people aged 10–24 years from 1990 to 2019 by sex and age group

10-24 years

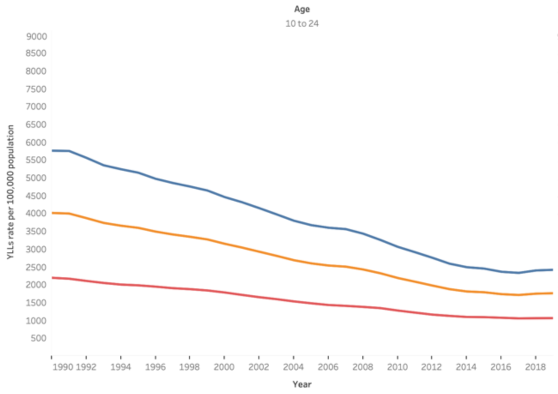

10-14 years

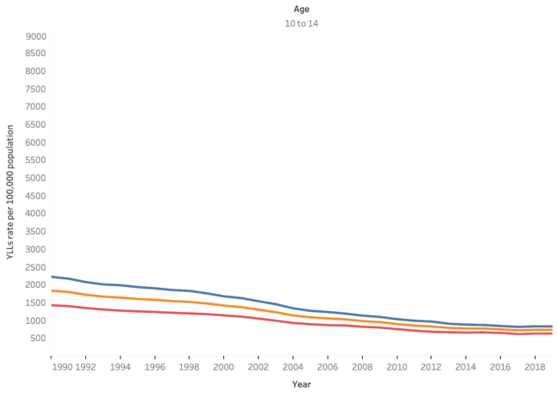

15-19 years

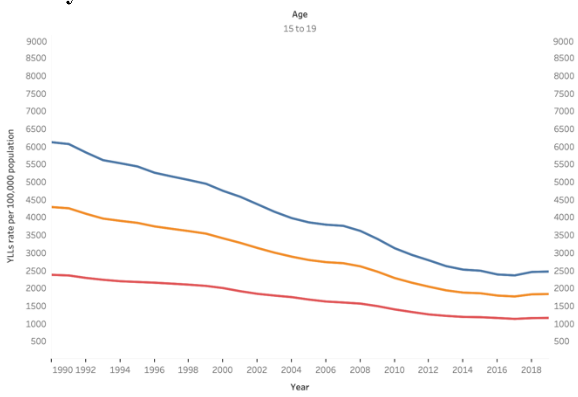

20-24 years

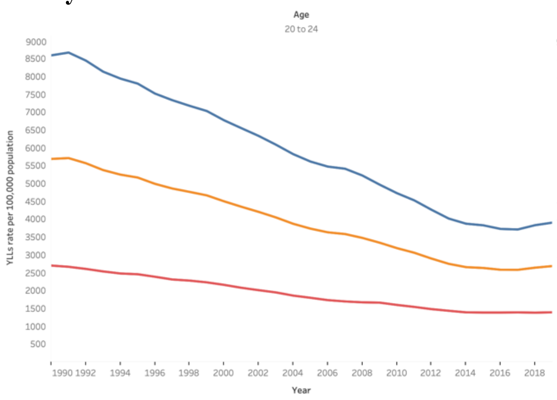

Sex
- Both sexes
- Females
- Males

Appendix Figure 6. YLL rates per 100,000 population, level 2 NCDs causes, in the European Union, in 2019, by age group and sex

Panel A

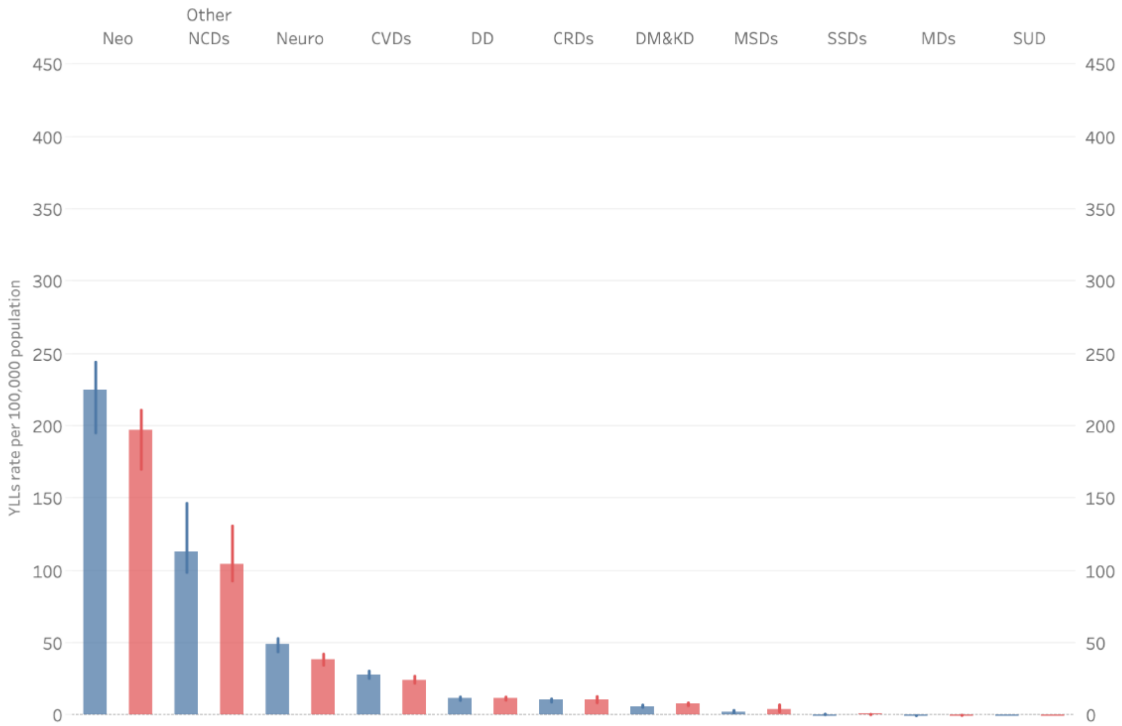

Panel B

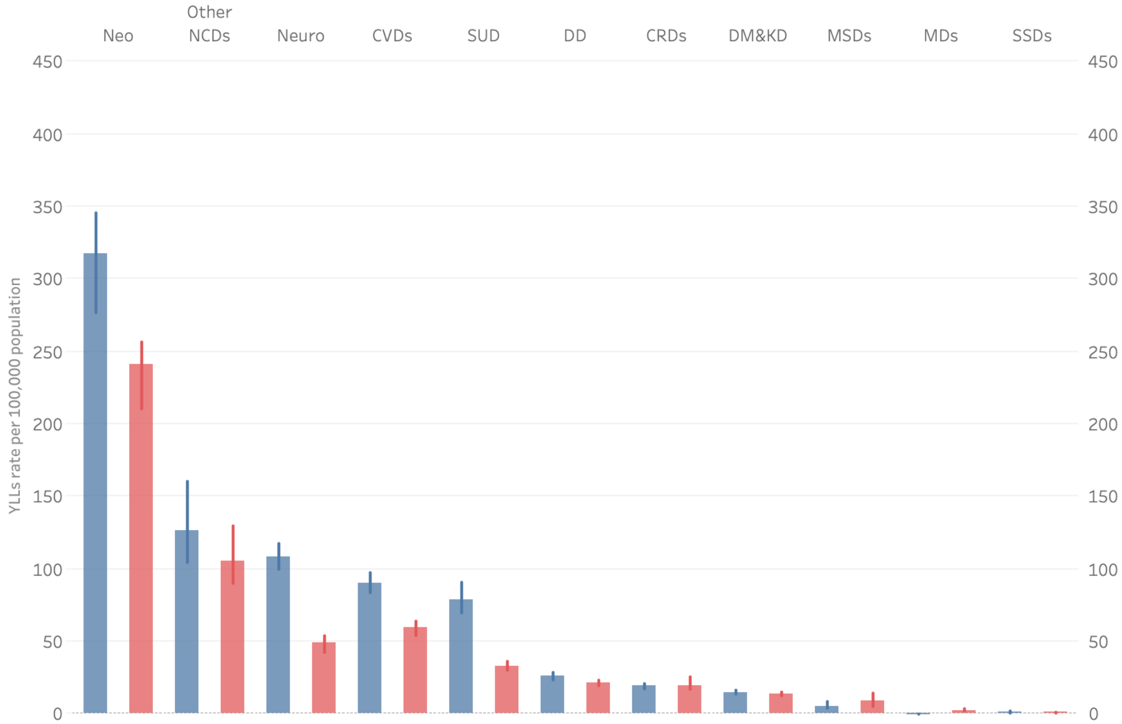

Panel C

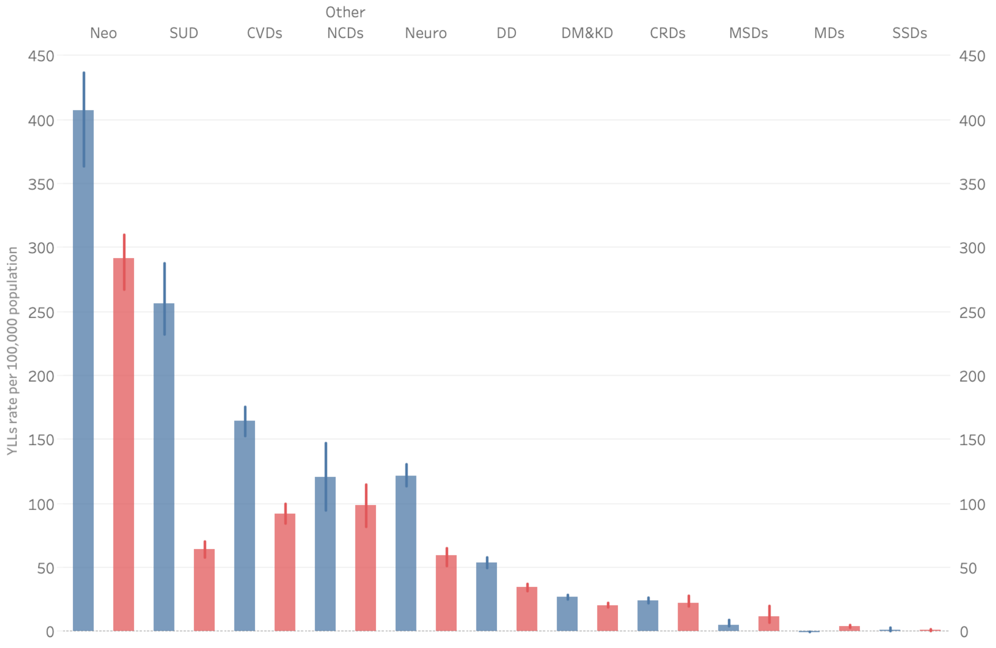

Sex
Males
Females

Panel A: 10-14 years old
Panel B: 15-19 years old
Panel C: 20-24 years old

Abbreviations: CRDs=Chronic respiratory diseases; CVDs=cardiovascular diseases; DD=Digestive diseases; DM&KD=Diabetes and kidney diseases; MDs=Mental health disorders; MSDs=Musculoskeletal disorders; Neo=Neoplasms; Neuro= Neurological disorders; Other NCDs=Other non-communicable diseases; SSDs=Skin and subcutaneous diseases; SUD=Substance use disorders.

Appendix Figure 7. YLL rates per 100,000 population, first 20 level 3 NCDs causes, in people 10-24 years old, in the European Union, 2019, by sex

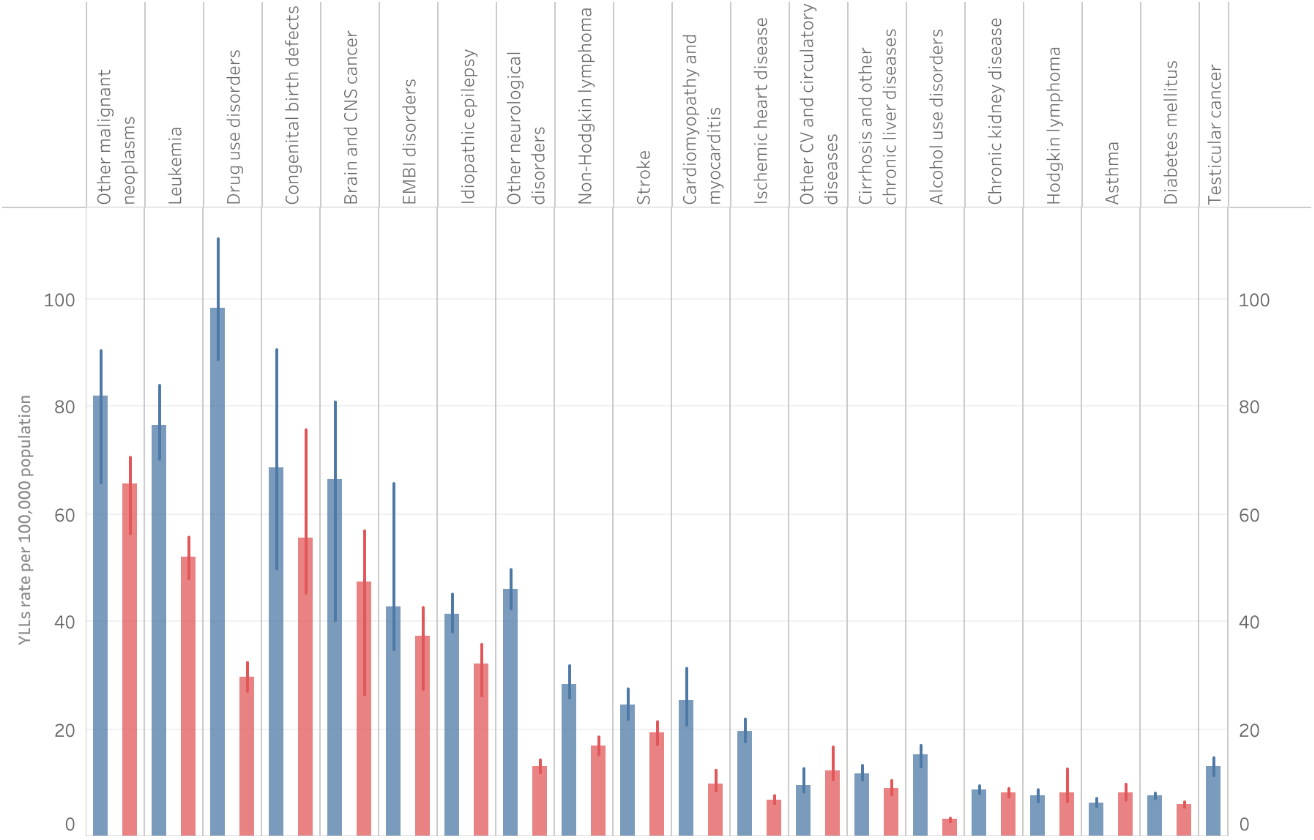

Sex
Males
Females

Abbreviations: EMBI=Endocrine, metabolic, blood, and immune disorders; CV=cardiovascular

Appendix Figure 8. YLL rates per 100,000 population (Panel A), and in percentage (Panel B), NCDs level 2, both sexes, 10-24 years old, 2019, by location

Panel A

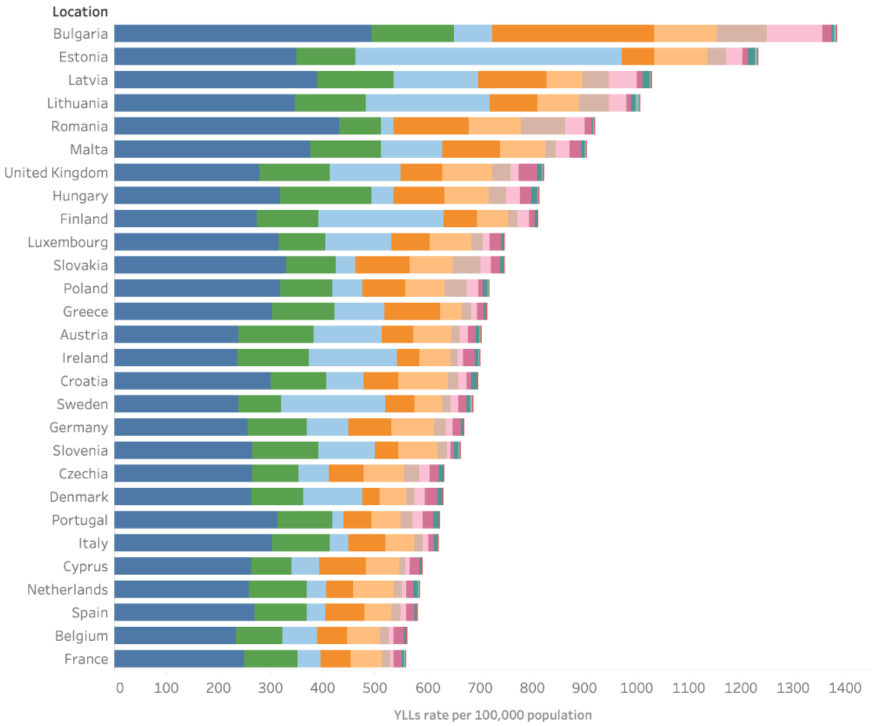

Panel B

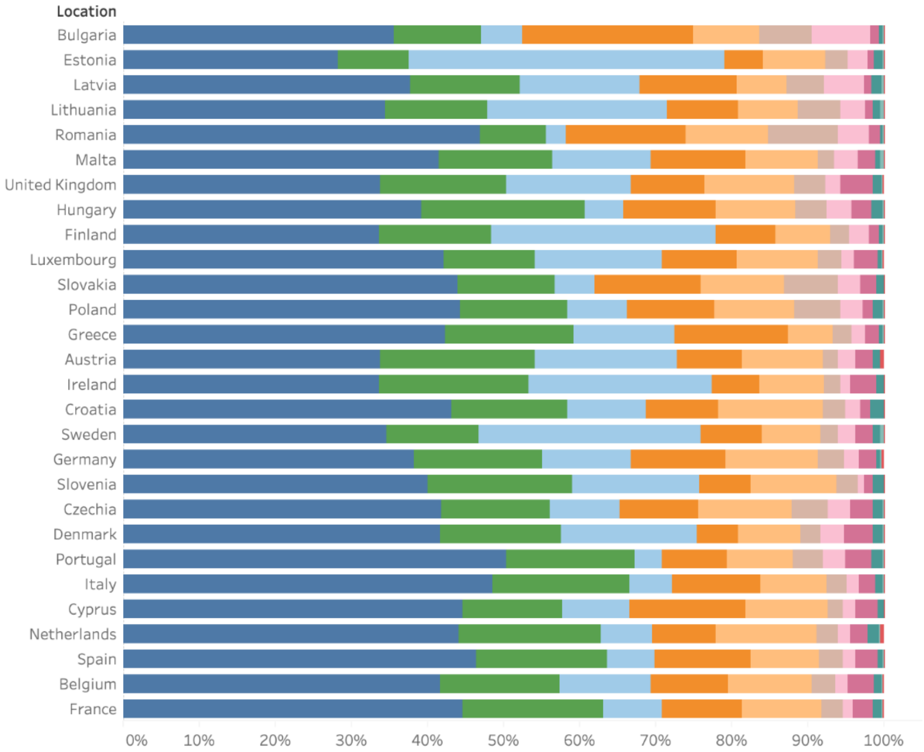

Legend:

- Chronic respiratory diseases
- CVDs
- Diabetes and kidney diseases
- Digestive diseases
- Mental health disorders
- Musculoskeletal disorders
- Neoplasms
- Neurological disorders
- Other NCDs*
- Sense organ diseases
- Skin and subcutaneous diseases
- Substance use disorders

Abbreviations: NCDs=non-communicable diseases; CVDs=cardiovascular diseases

*Note: This aggregate cause contains the following Level 3 causes: congenital birth defects; urinary diseases; gynaecological diseases; haemoglobinopathies and haemolytic anaemias; endocrine, metabolic, blood, and immune disorders; oral disorders.

Appendix Figure 9. Level 2 NCD causes, in the European Union, in people aged 10-24 years, both sexes, from 1990 to 2019: YLLs rate per 100,000 population

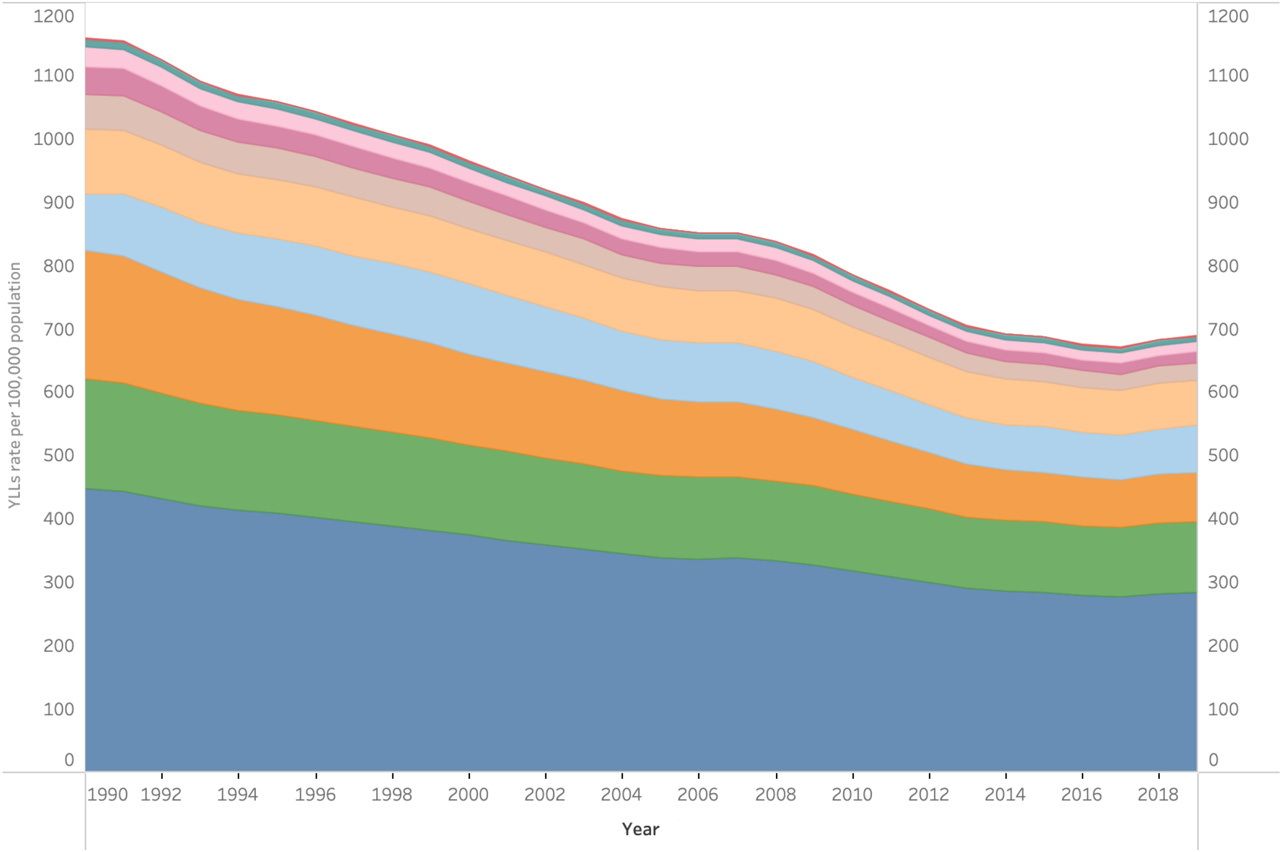

Legend:
- Chronic respiratory diseases
- CVDs
- Diabetes and kidney diseases
- Digestive diseases
- Mental health disorders
- Musculoskeletal disorders
- Neoplasms
- Neurological disorders
- Other NCDs*
- Sense organ diseases
- Skin and subcutaneous diseases
- Substance use disorders

Abbreviations: NCDs=non-communicable diseases; CVDs=cardiovascular diseases

*Note: This aggregate cause contains the following Level 3 causes: congenital birth defects; urinary diseases; gynecological diseases; hemoglobinopathies and hemolytic anemias; endocrine, metabolic, blood, and immune disorders; oral disorders.

Appendix Figure 10. All-cause YLD rates per 100,000 population, in people aged 10–24 years from 1990 to 2019 by sex and age group

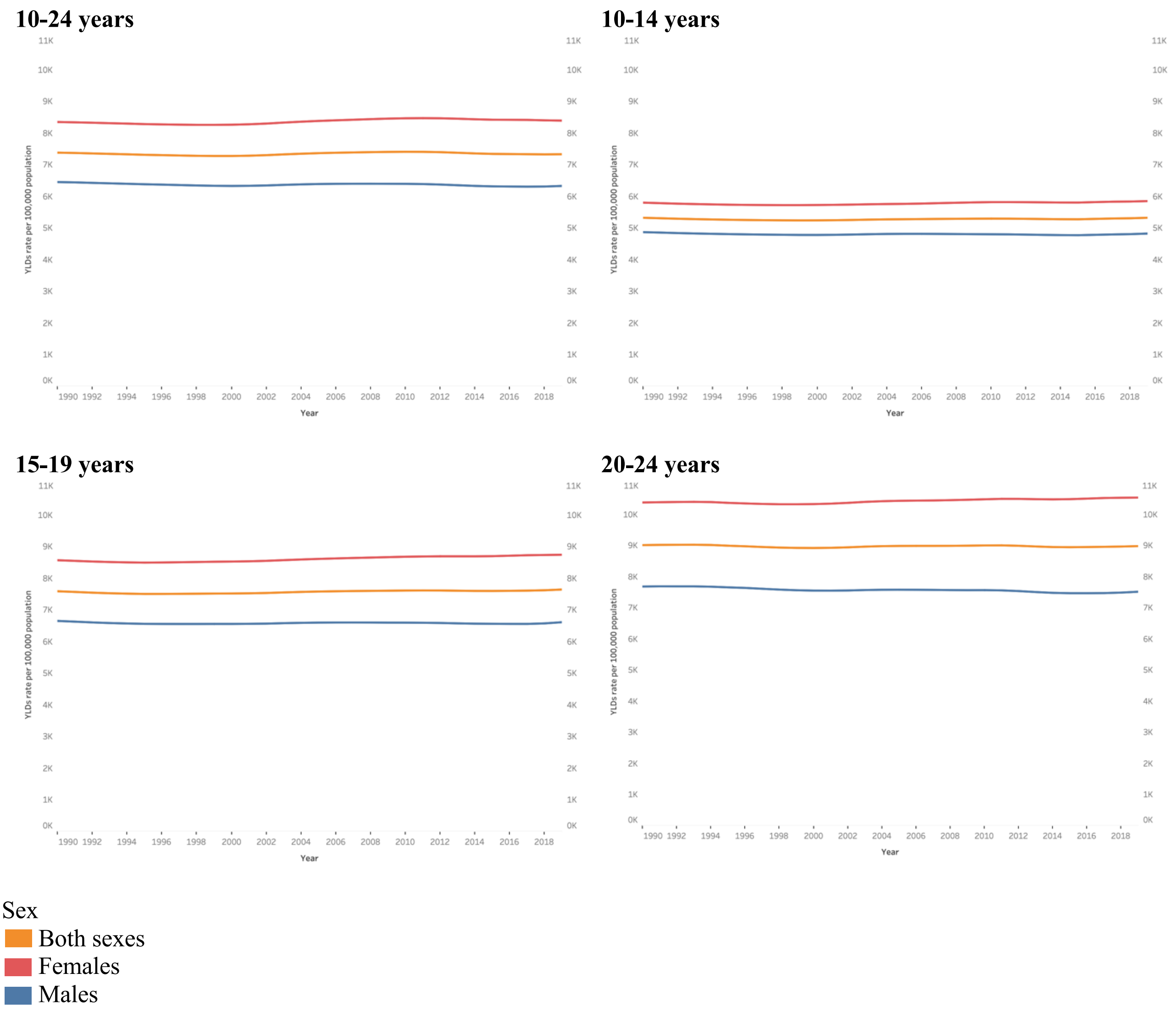

Appendix Figure 11. YLD rates per 100,000 population, level 2 NCDs causes, European Union, 2019, by age group and sex

Panel A

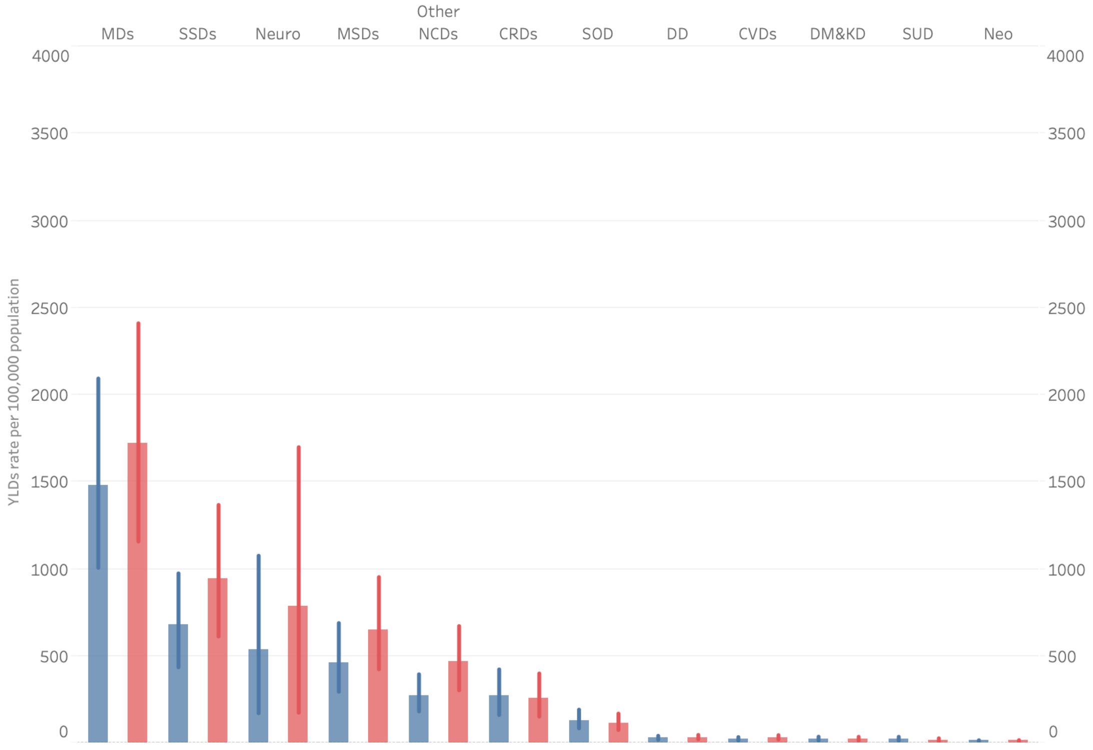

Panel B

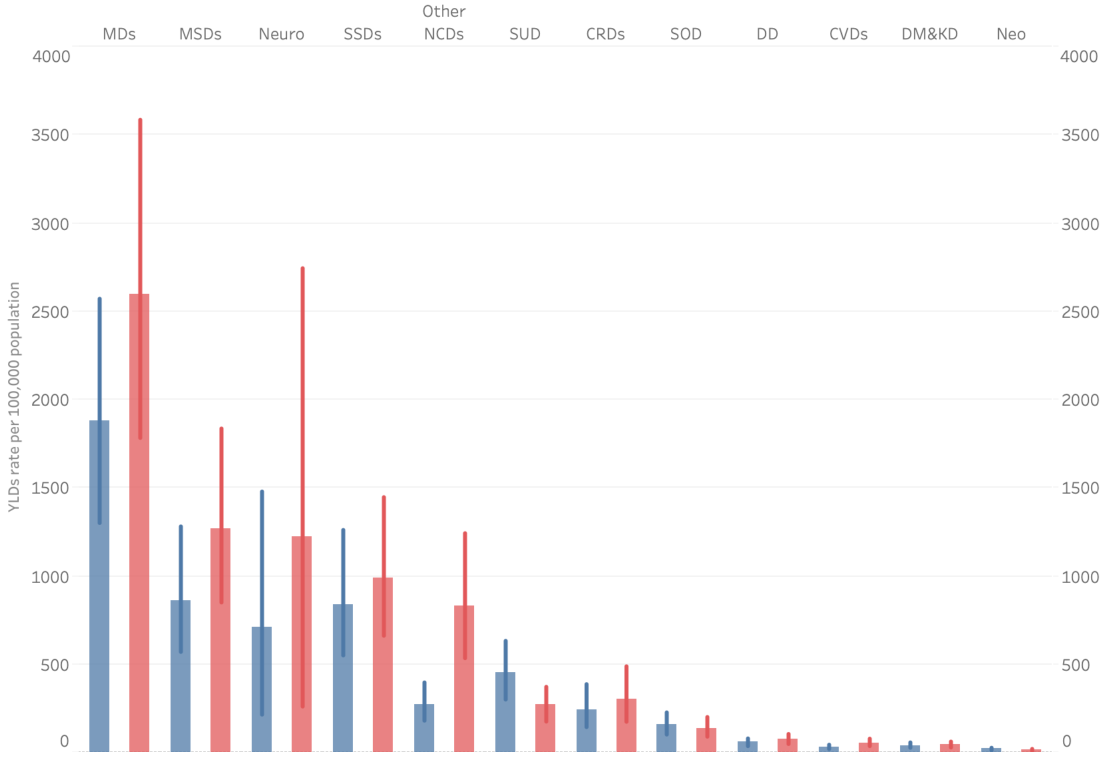

Panel C

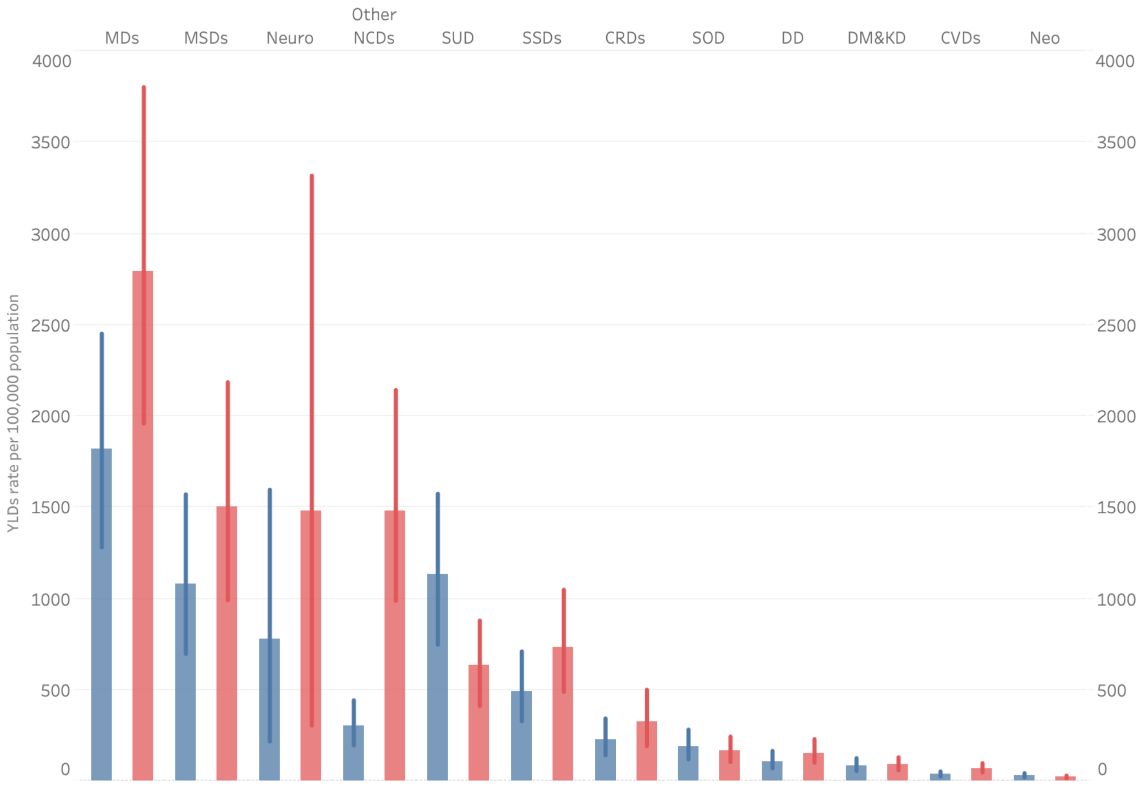

Sex
Males
Females

Panel A: 10-14 years old
Panel B: 15-19 years old
Panel C: 20-24 years old

Abbreviations: CRDs=Chronic respiratory diseases; CVDs=cardiovascular diseases; DD=Digestive diseases; DM&KD=Diabetes and kidney diseases; MDs=Mental health disorders; MSDs=Musculoskeletal disorders; Neo=Neoplasms; Neuro= Neurological disorders; Other NCDs=Other non-communicable diseases; SOD=Sense organ diseases; SSDs=Skin and subcutaneous diseases; SUD=Substance use disorders.

Appendix Figure 12. YLD rates per 100,000 population, first 20 level 3 NCDs causes, in people 10-24 years old, in the European Union, 2019, by sex

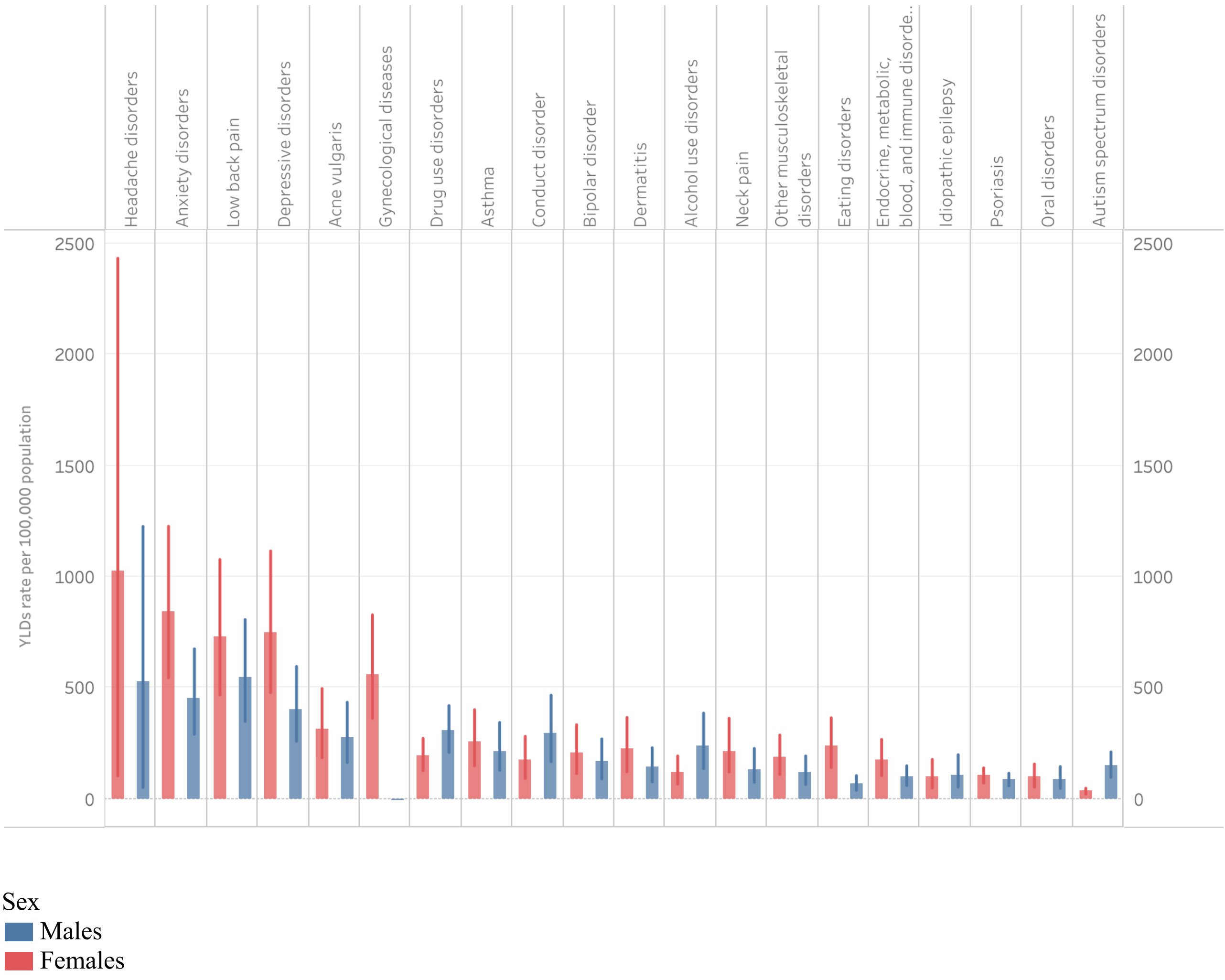

Appendix Figure 13. YLD rates per 100,000 population (Panel A), and in percentage (Panel B), NCDs level 2, both sexes, 10-24 years old, 2019, by location

Panel A

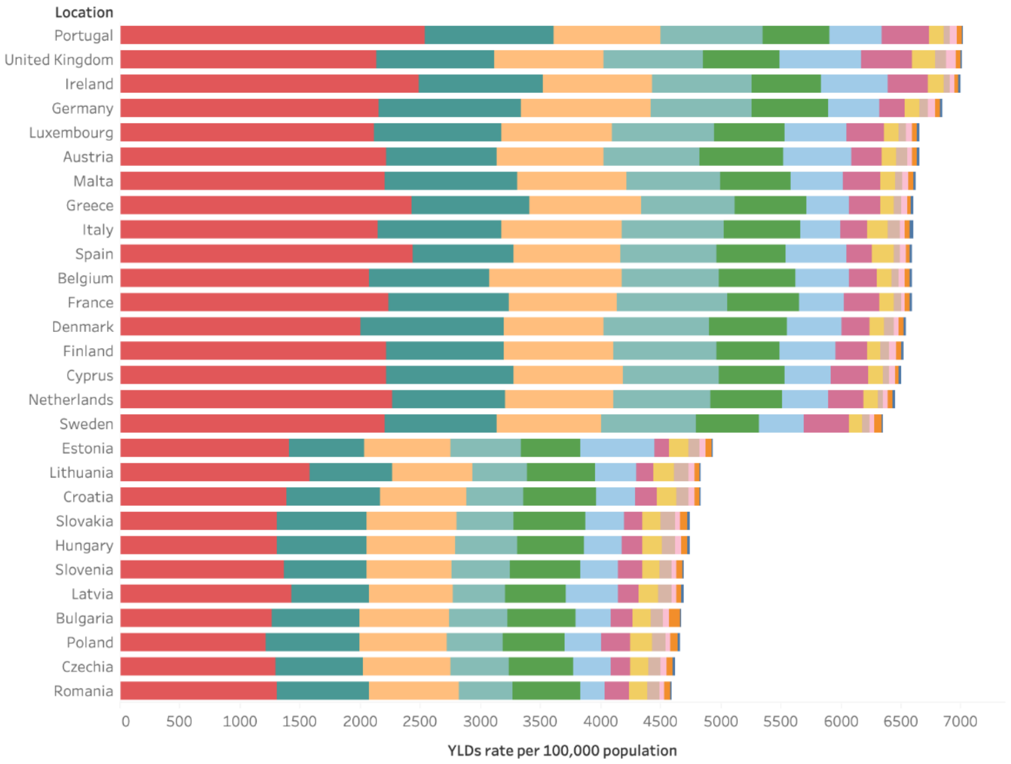

Panel B

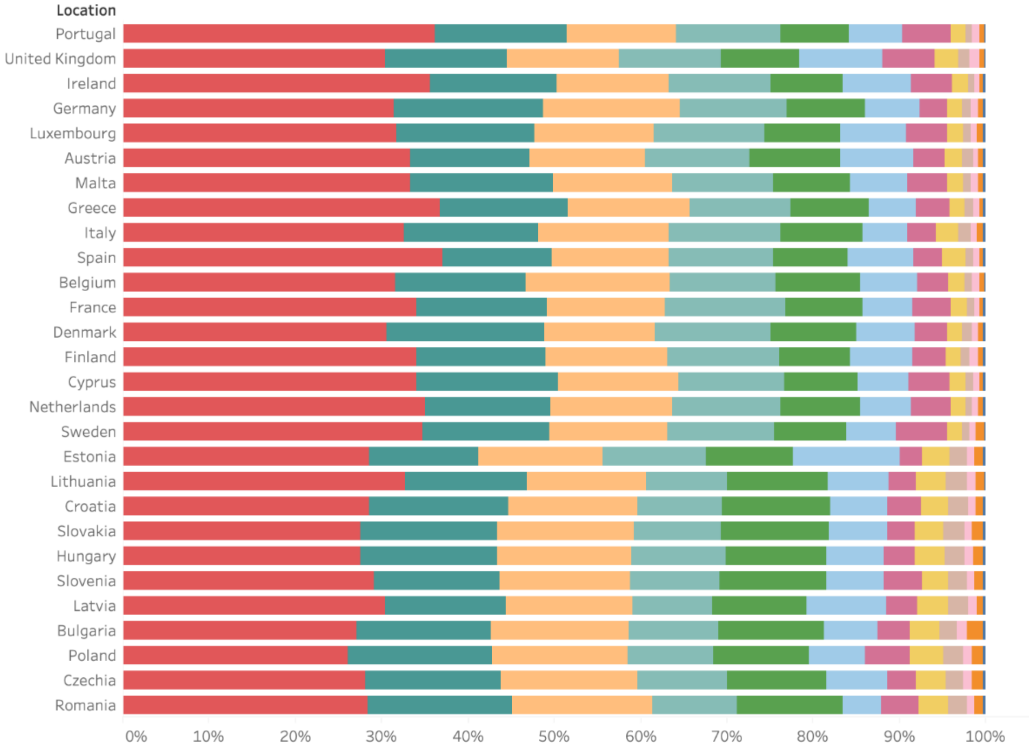

Legend:
- Chronic respiratory diseases
- CVDs
- Diabetes and kidney diseases
- Digestive diseases
- Mental health disorders
- Musculoskeletal disorders
- Neoplasms
- Neurological disorders
- Other NCDs*
- Sense organ diseases
- Skin and subcutaneous diseases
- Substance use disorders

Abbreviations: NCDs=non-communicable diseases; CVDs=cardiovascular diseases

*Note: This aggregate cause contains the following Level 3 causes: congenital birth defects; urinary diseases; gynaecological diseases; haemoglobinopathies and haemolytic anaemias; endocrine, metabolic, blood, and immune disorders; oral disorders.

Appendix Figure 14. Level 2 NCD causes, in the European Union, in people aged 10-24 years, both sexes, from 1990 to 2019: YLDs rate per 100,000 population

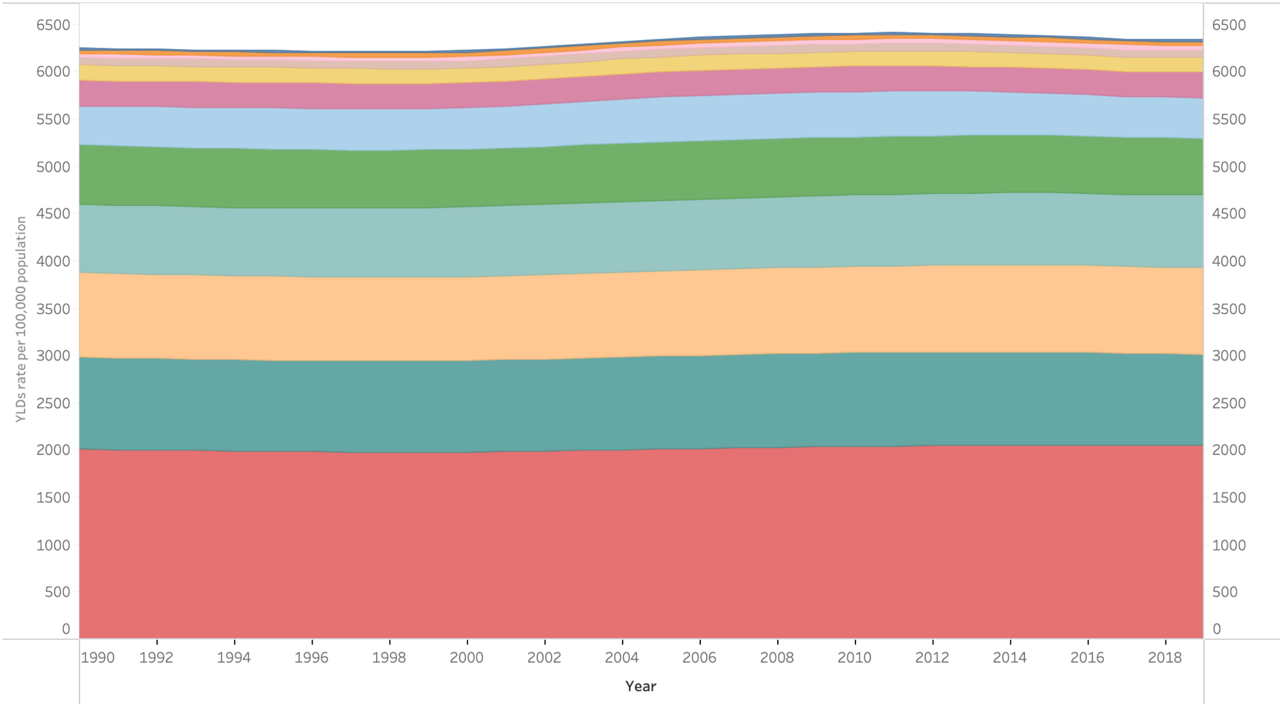

Legend:
- Chronic respiratory diseases
- CVDs
- Diabetes and kidney diseases
- Digestive diseases
- Mental health disorders
- Musculoskeletal disorders
- Neoplasms
- Neurological disorders
- Other NCDs*
- Sense organ diseases
- Skin and subcutaneous diseases
- Substance use disorders

Abbreviations: NCDs=non-communicable diseases; CVDs=cardiovascular diseases

*Note: This aggregate cause contains the following Level 3 causes: congenital birth defects; urinary diseases; gynecological diseases; hemoglobinopathies and hemolytic anemias; endocrine, metabolic, blood, and immune disorders; oral disorders.

Appendix figure 15. All-cause DALY rates per 100,000 population, in people aged 10–24 years from 1990 to 2019 by sex and age group

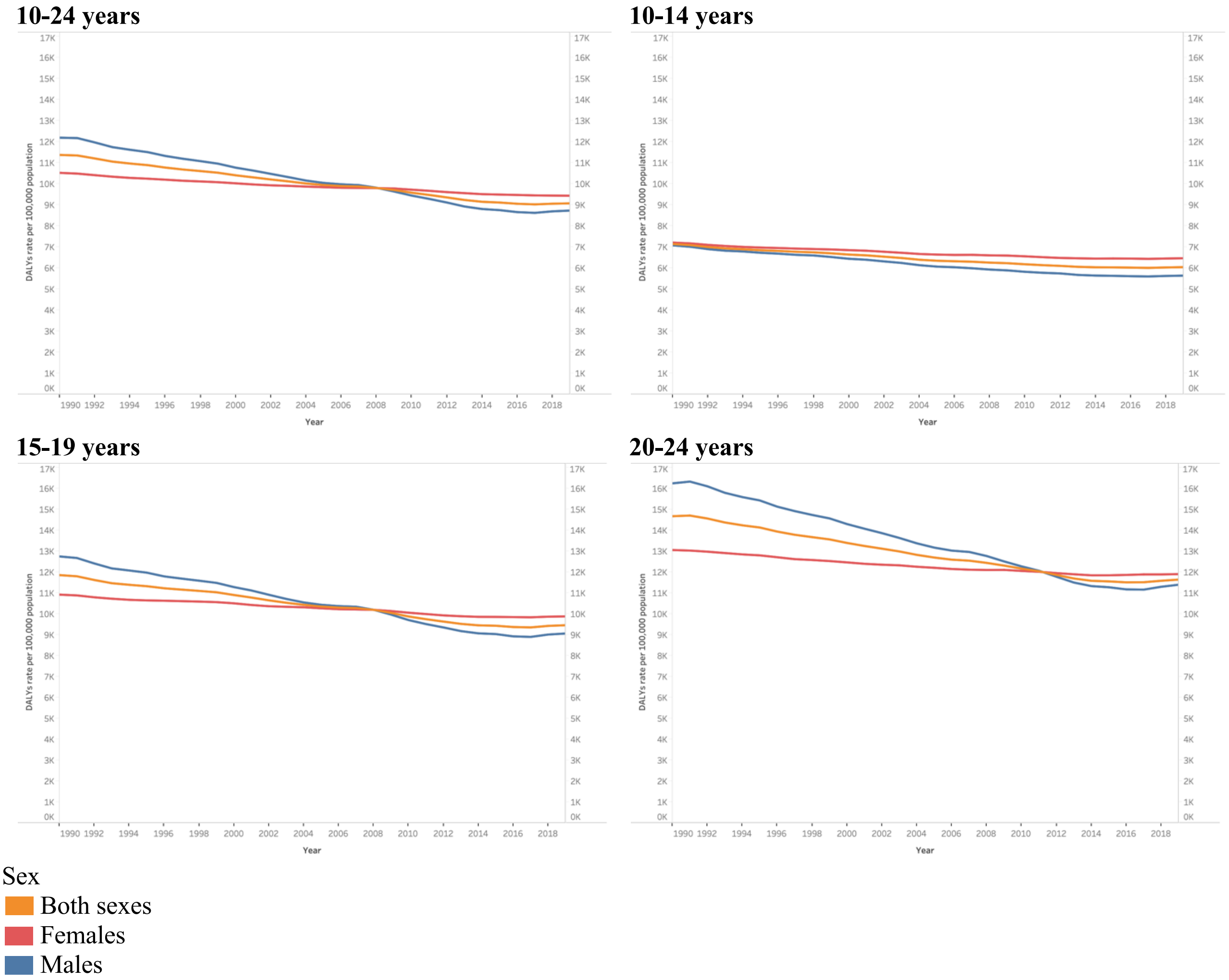

Appendix Figure 16. DALY rates per 100,000 population, level 2 NCDs causes, European Union, 2019, by age group and sex

Panel A

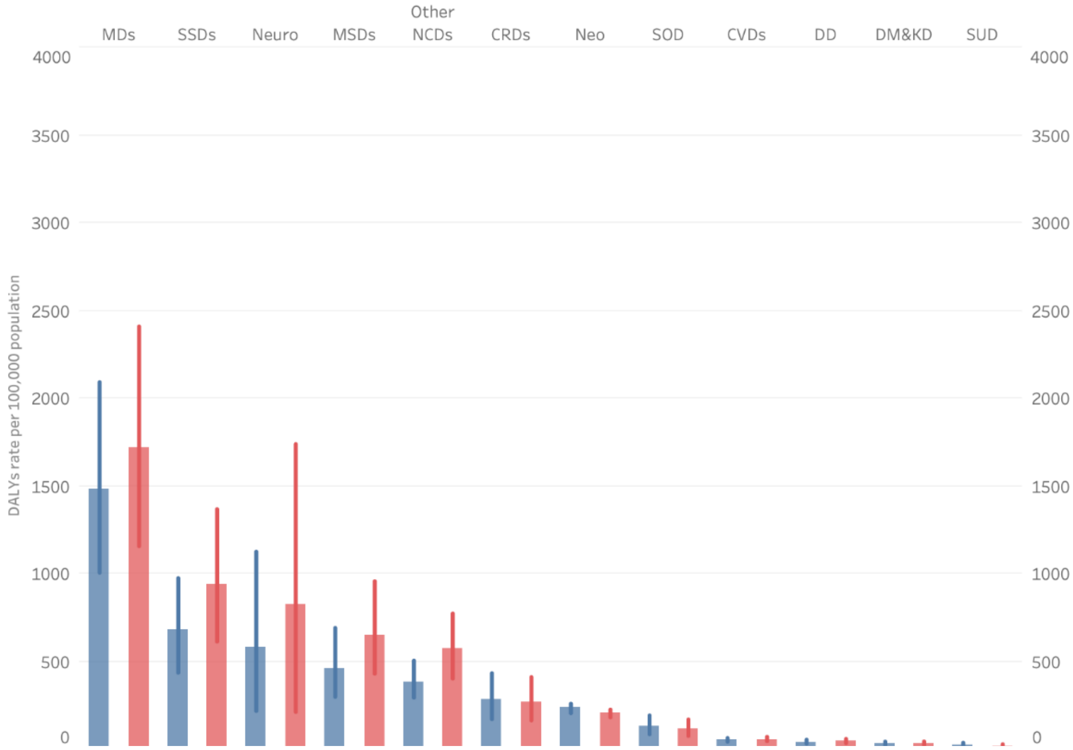

Panel B

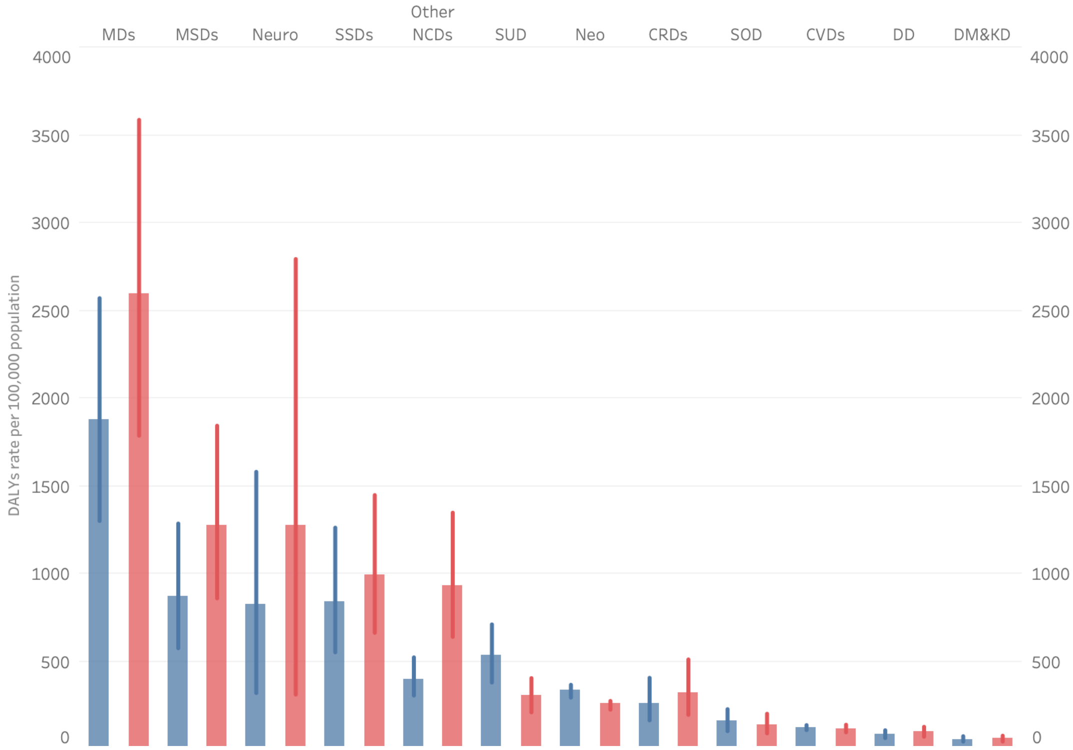

Panel C

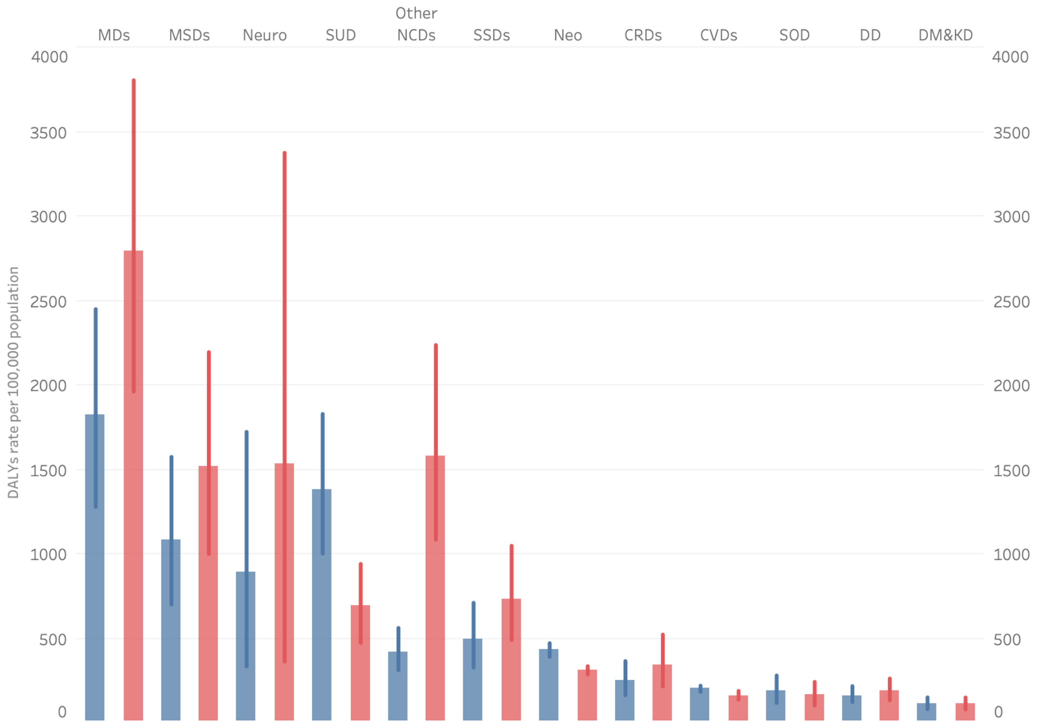

Sex
Males
Females

Panel A: 10-14 years old
Panel B: 15-19 years old
Panel C: 20-24 years old

Abbreviations: CRDs=Chronic respiratory diseases; CVDs=cardiovascular diseases; DD=Digestive diseases; DM&KD=Diabetes and kidney diseases; MDs=Mental health disorders; MSDs=Musculoskeletal disorders; Neo=Neoplasms; Neuro= Neurological disorders; Other NCDs=Other non-communicable diseases; SOD=Sense organ diseases; SSDs=Skin and subcutaneous diseases; SUD=Substance use disorders.

Appendix Figure 17. DALY rates per 100,000 population, first 20 level 3 NCDs causes, in people 10-24 years old, in the European Union, 2019, by sex

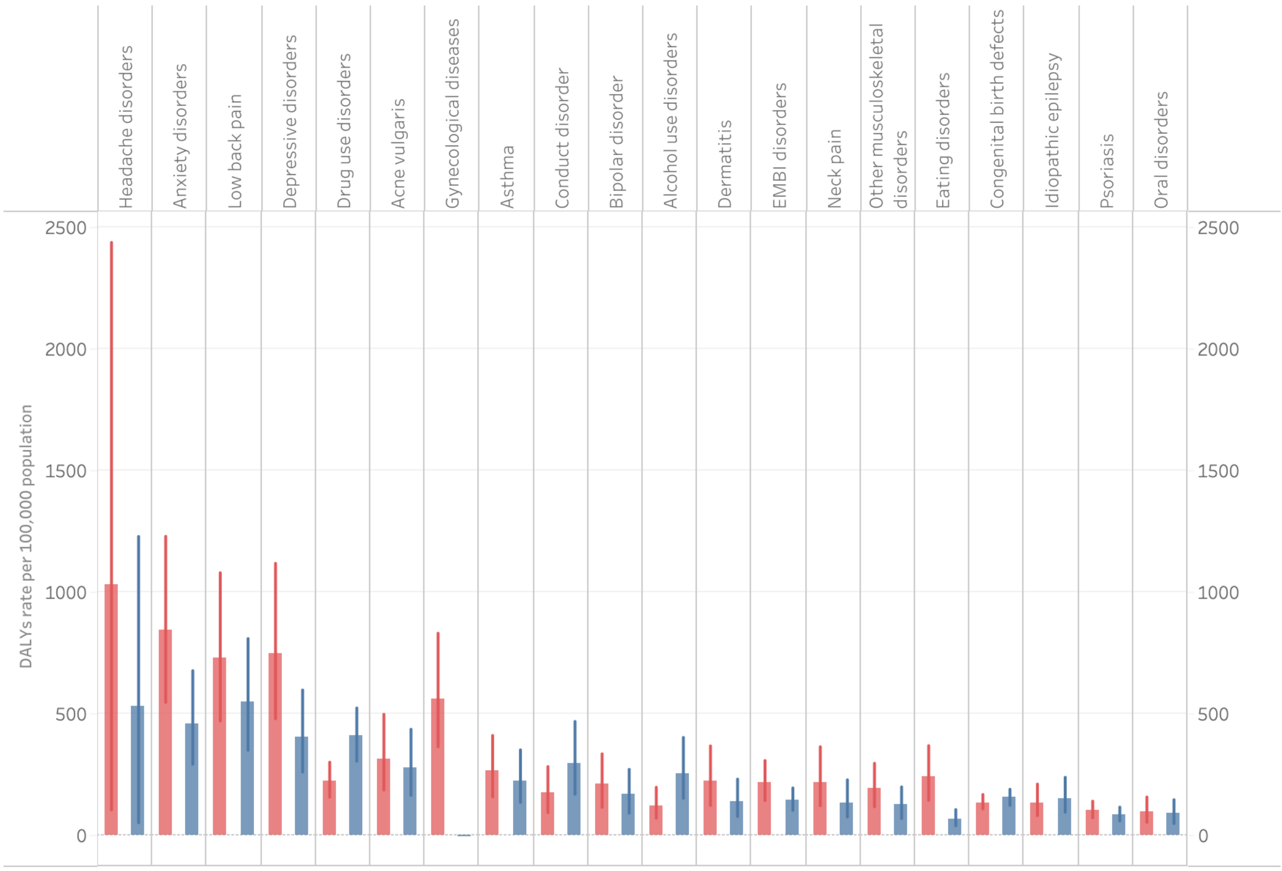

Sex
Males
Females

Abbreviations: EMBI=Endocrine, metabolic, blood, and immune disorders

Appendix Figure 18. DALY rates per 100,000 population, among 10-24 years old adolescents, both sexes, 2019, level 2 NCDs

Cardiovascular diseases

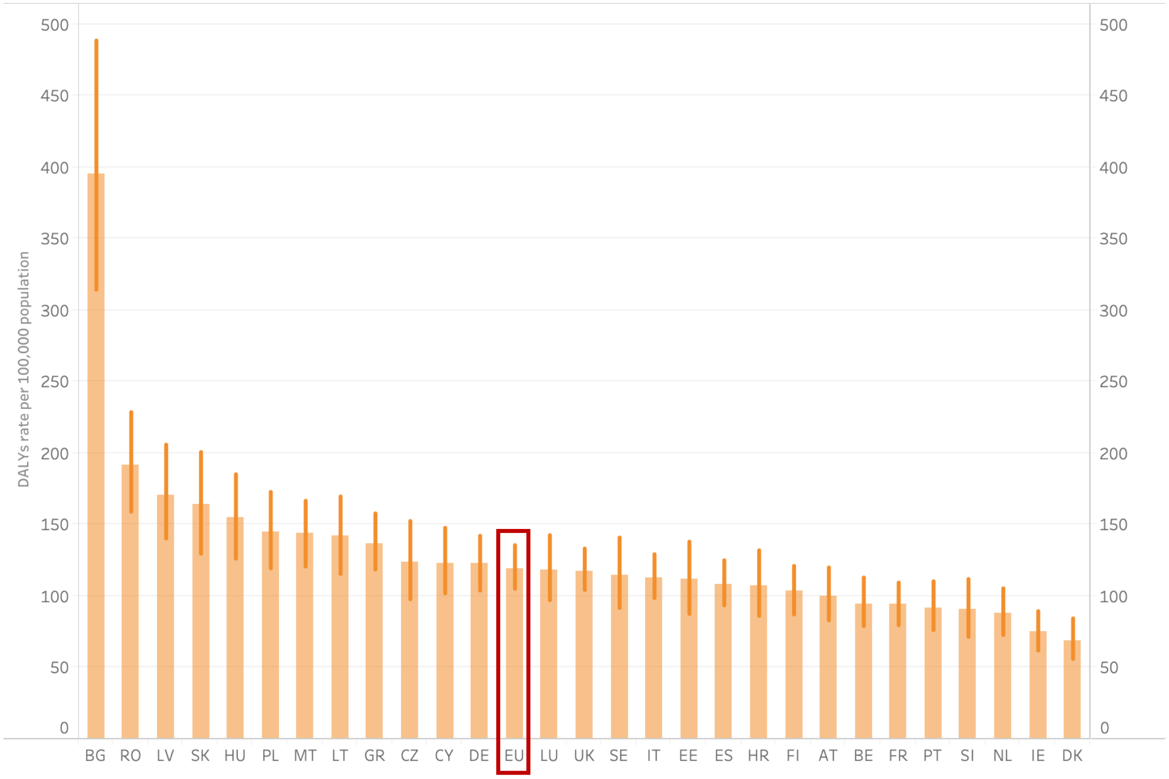

Digestive diseases

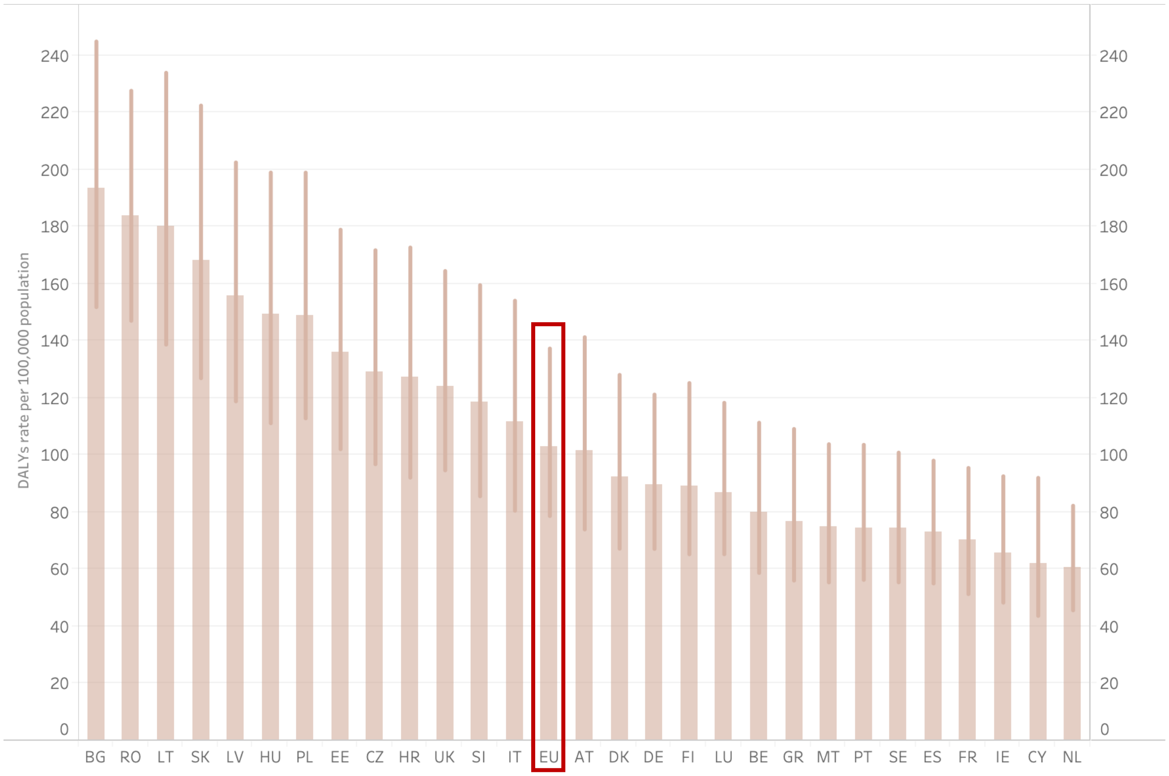

Neoplasms

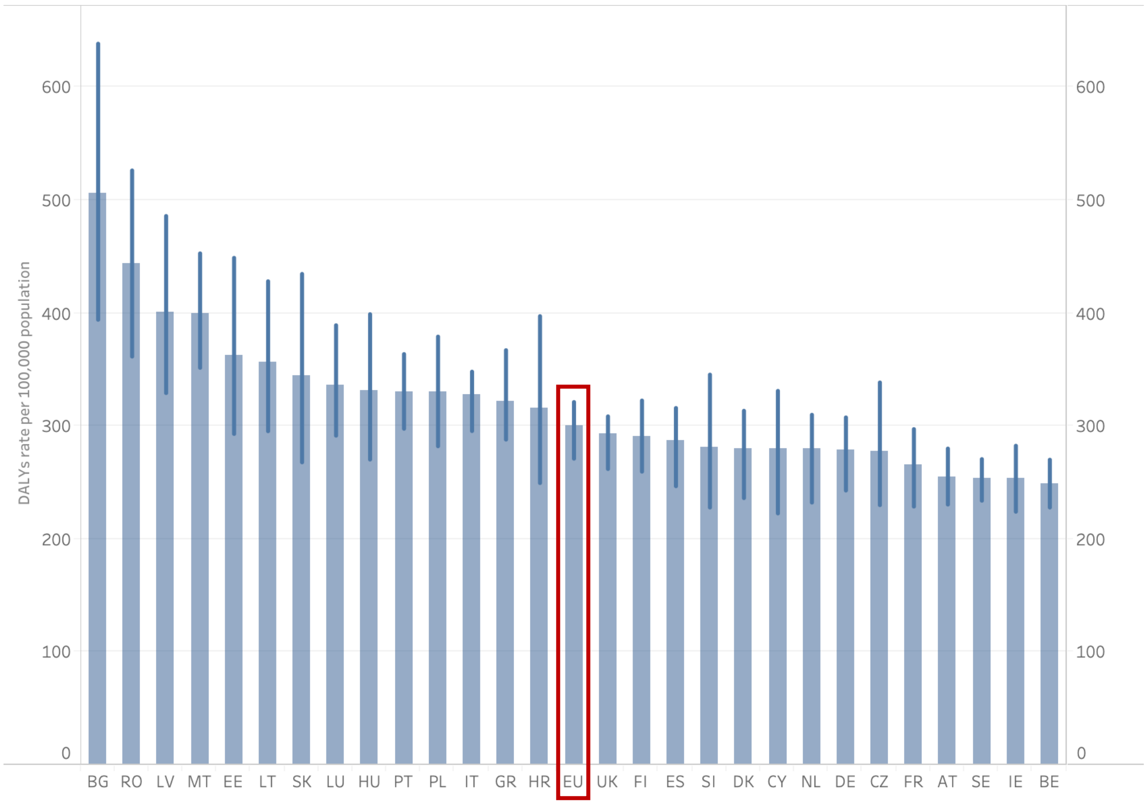

Substance use disorders

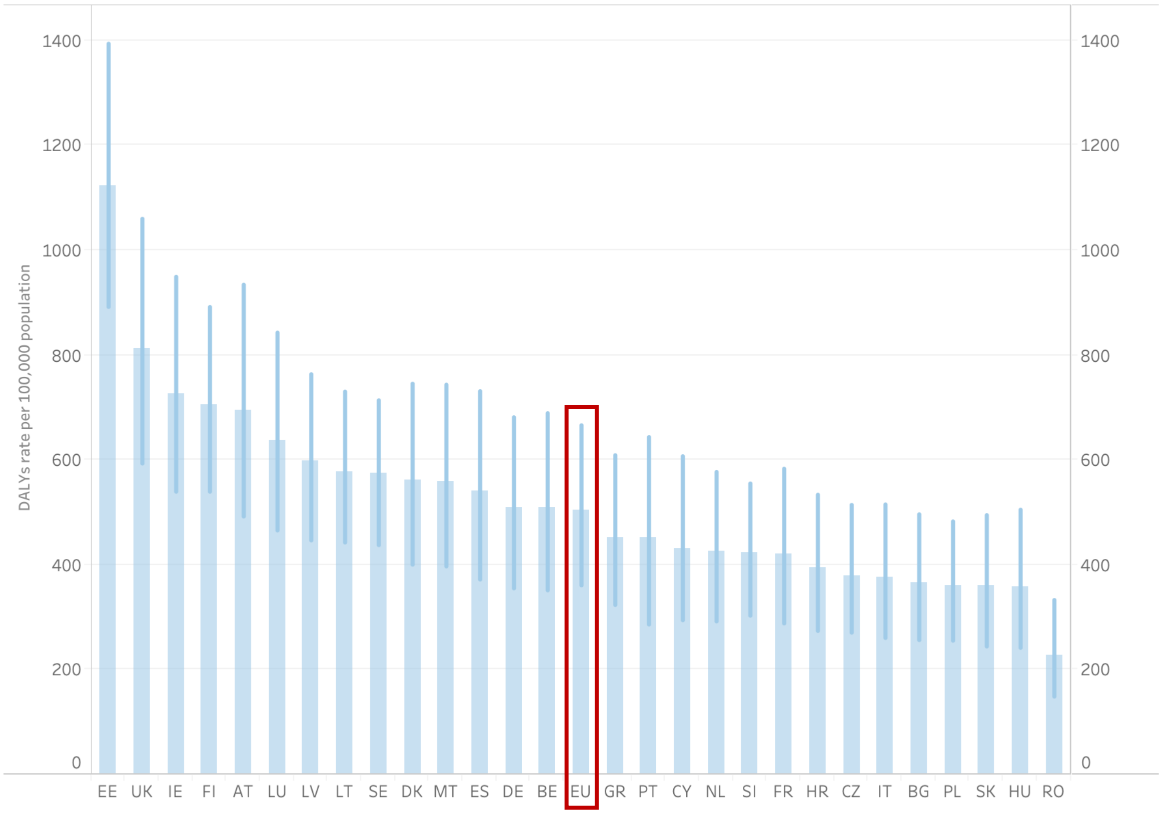

Diabetes and kidney diseases

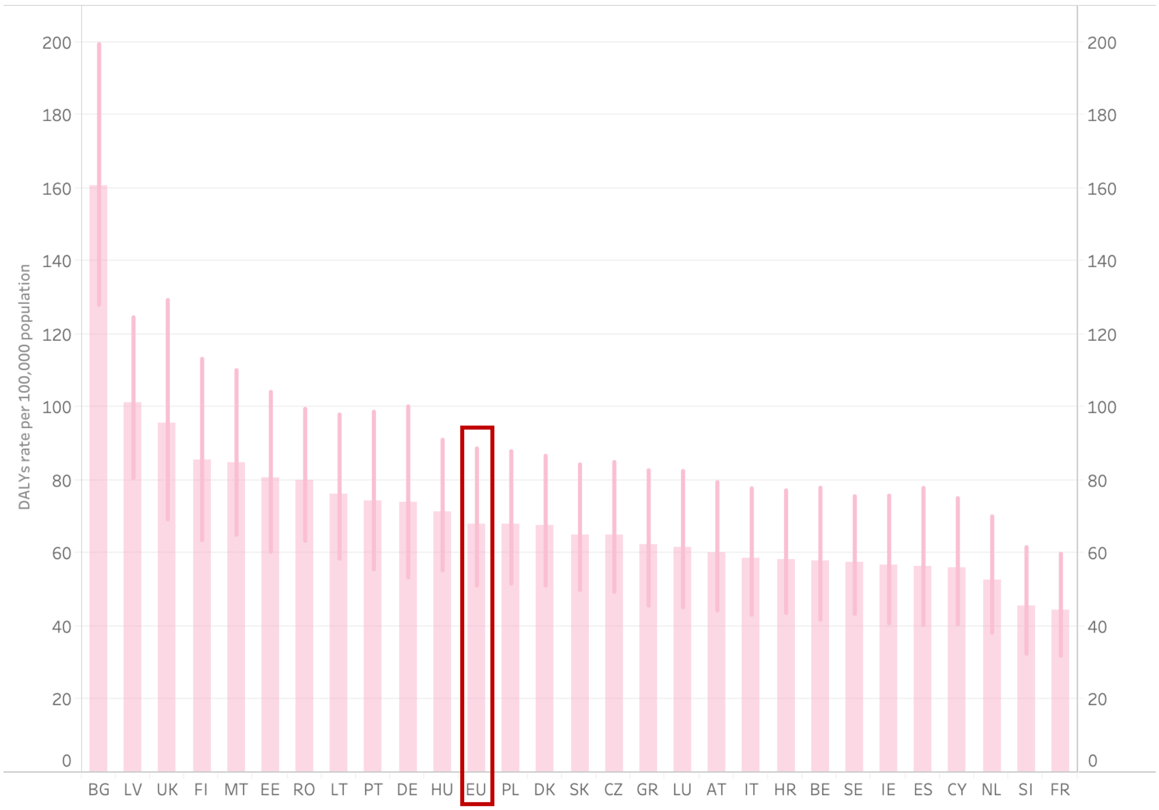

Abbreviations: BG=Bulgaria; LV=Latvia; RO=Romania; SI=Slovenia; HU=Hungary; PL=Poland; MT=Malta; LT=Lithuania; GR=Greece; CZ=Czechia; CY=Cyprus; DE=Germany; EU=European Union; LU= Luxembourg; UK=United Kingdom; SE=Sweden; IT=Italy; EE=Estonia; ES=Spain; HR=Croatia; FI=Finland; AT=Austria; BE=Belgium; FR=France; PT=Portugal; SK=Slovakia; NL=Netherlands; IE=Ireland; DK=Denmark

Appendix Figure 19. DALY rates per 100,000 population, NCDs level 2 causes, 10-24 years old, both sexes, in the European Union, trend from 1990-2019

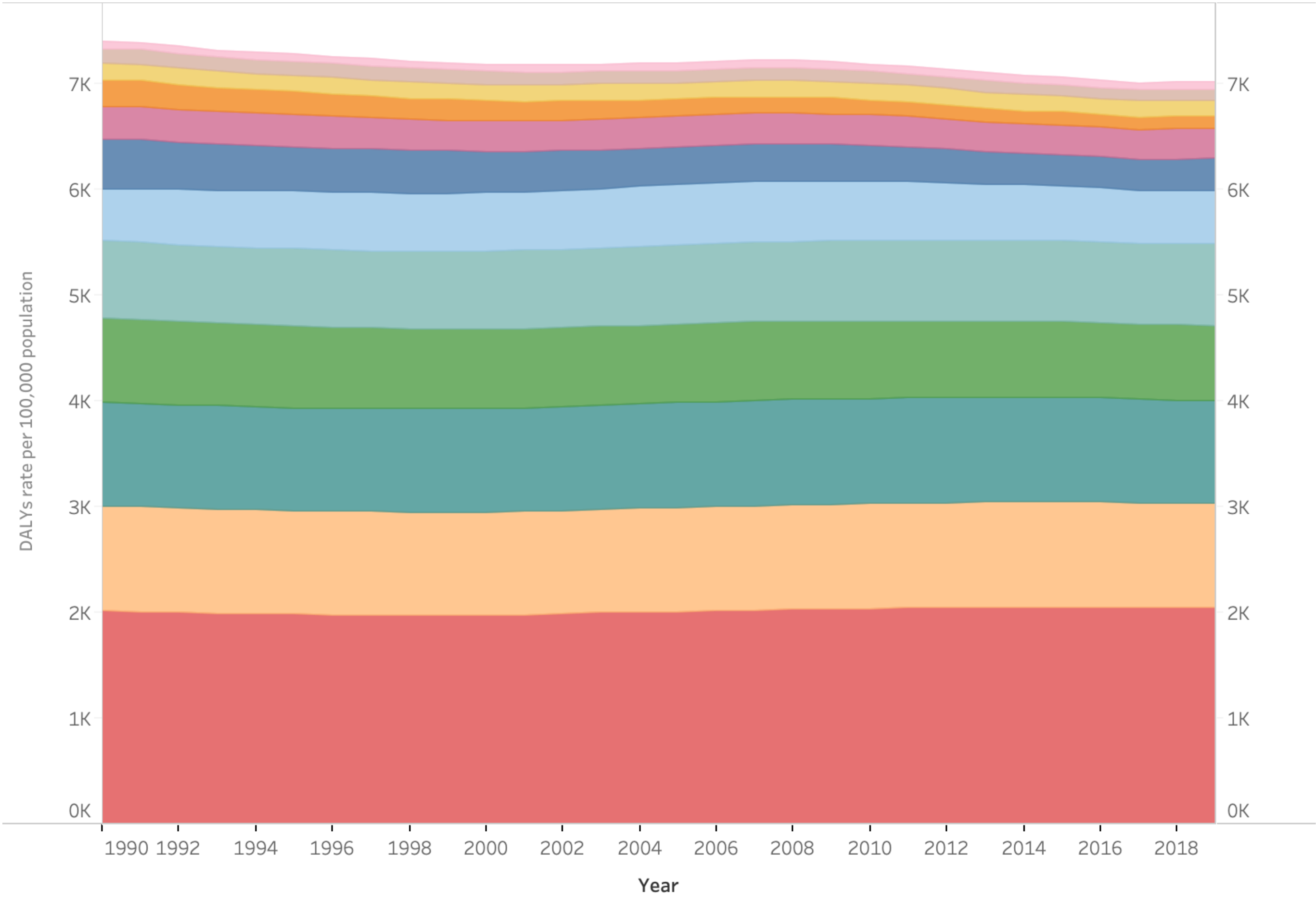

Legend:
- Chronic respiratory diseases
- CVDs
- Diabetes and kidney diseases
- Digestive diseases
- Mental health disorders
- Musculoskeletal disorders
- Neoplasms
- Neurological disorders
- Other NCDs*
- Sense organ diseases
- Skin and subcutaneous diseases
- Substance use disorders

Abbreviations: NCDs=non-communicable diseases; CVDs=cardiovascular diseases

*Note: This aggregate cause contains the following Level 3 causes: congenital birth defects; urinary diseases; gynaecological diseases; haemoglobinopathies and haemolytic anaemias; endocrine, metabolic, blood, and immune disorders; oral disorders.

Appendix Figure 20. Relation between DALY rates per 100,000 population of five NCDs and Socio Demographic Index (SDI), in 28 EU Member States, year 2019, both sexes, age 10-24

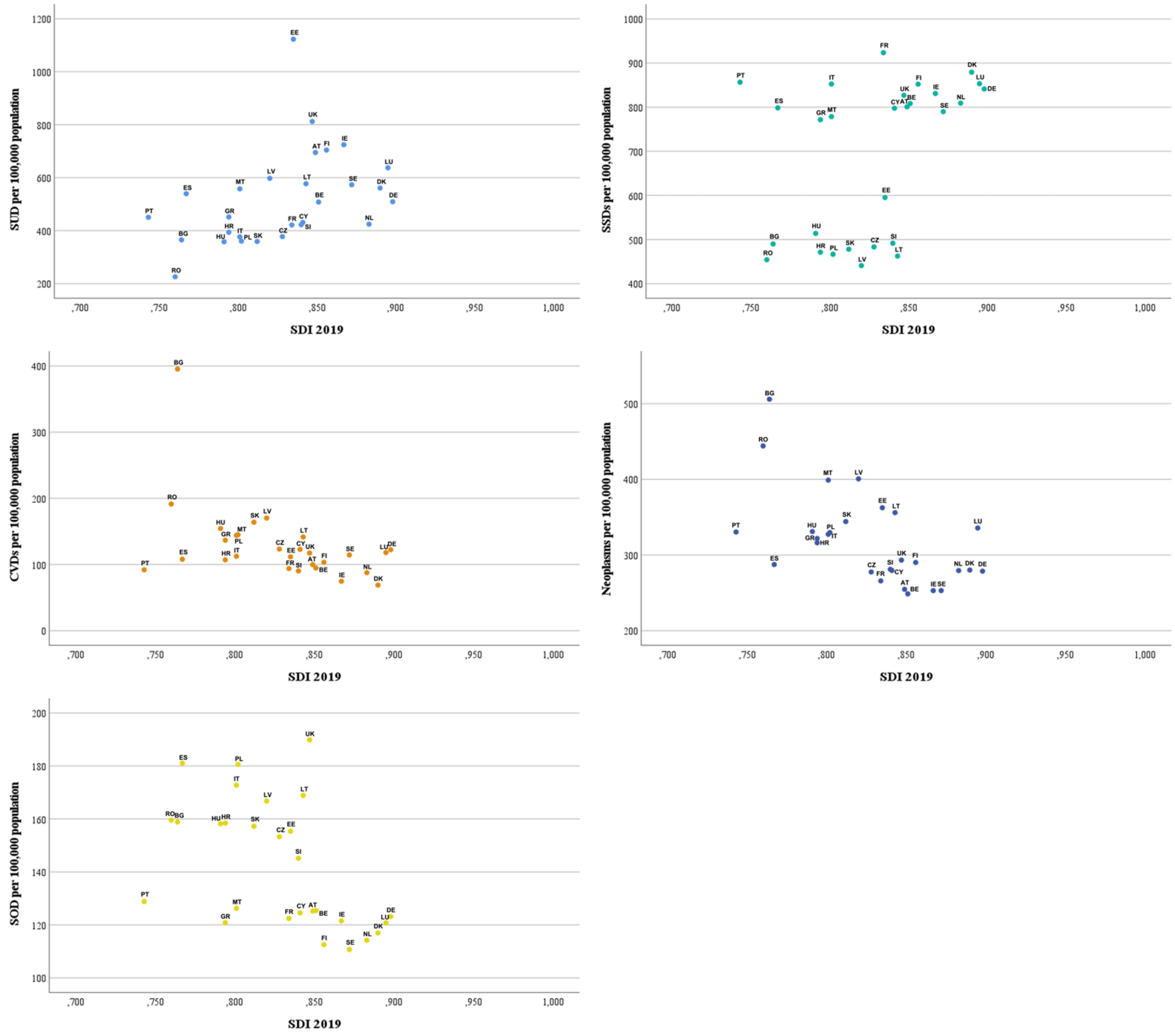

CVDs
Neoplasms
Sense organ diseases
Skin and subcutaneous diseases
Substance use disorders

Abbreviations: CVDs=cardiovascular diseases; SOD=Sense organ diseases; SSDs=Skin and subcutaneous diseases; SUD=Substance use disorders; AT=Austria; BE=Belgium; BG=Bulgaria; HR=Croatia; CY=Cyprus; CZ=Czechia; DK=Denmark; EE=Estonia; FI=Finland; FR=France; DE=Germany; GR=Greece; HU=Hungary; IE=Ireland; IT=Italy; LV=Latvia; LT=Lithuania; LU= Luxembourg; MT=Malta; NL=Netherlands; PL=Poland; PT=Portugal; RO=Romania; SK=Slovakia; SI=Slovenia; ES=Spain; SE=Sweden; UK=United Kingdom; SDI=socio-demographic index

Appendix table 1. Global Burden of Disease 2019 Cause Hierarchy

Cause ID	Cause Name	Hierarchy Level	Cause Outline
294	**All causes**	0	Total
295	**Communicable, maternal, neonatal, and nutritional diseases**	1	A
409	**Non-communicable diseases**	1	B
410	**Neoplasms**	2	B.1
444	Lip and oral cavity cancer	3	B.1.1
447	Nasopharynx cancer	3	B.1.2
450	Other pharynx cancer	3	B.1.3
411	Esophageal cancer	3	B.1.4
414	Stomach cancer	3	B.1.5
441	Colon and rectum cancer	3	B.1.6
417	Liver cancer	3	B.1.7
453	Gallbladder and biliary tract cancer	3	B.1.8
456	Pancreatic cancer	3	B.1.9
423	Larynx cancer	3	B.1.10
426	Tracheal, bronchus, and lung cancer	3	B.1.11
459	Malignant skin melanoma	3	B.1.12
462	Non-melanoma skin cancer	3	B.1.13
429	Breast cancer	3	B.1.14
432	Cervical cancer	3	B.1.15
435	Uterine cancer	3	B.1.16
465	Ovarian cancer	3	B.1.17
438	Prostate cancer	3	B.1.18
468	Testicular cancer	3	B.1.19
471	Kidney cancer	3	B.1.20
474	Bladder cancer	3	B.1.21
477	Brain and nervous system cancer	3	B.1.22
480	Thyroid cancer	3	B.1.23
483	Mesothelioma	3	B.1.24
484	Hodgkin lymphoma	3	B.1.25
485	Non-Hodgkin lymphoma	3	B.1.26
486	Multiple myeloma	3	B.1.27
487	Leukemia	3	B.1.28
489	Other malignant neoplasms	3	B.1.29
490	Other neoplasms	3	B.1.30
491	**Cardiovascular diseases**	2	B.2
492	Rheumatic heart disease	3	B.2.1
493	Ischemic heart disease	3	B.2.2
494	Stroke	3	B.2.3
498	Hypertensive heart disease	3	B.2.4
504	Non-rheumatic valvular heart disease	3	B.2.5
499	Cardiomyopathy and myocarditis	3	B.2.6
500	Atrial fibrillation and flutter	3	B.2.7
501	Aortic aneurysm	3	B.2.8
502	Peripheral artery disease	3	B.2.9
503	Endocarditis	3	B.2.10

507	Other cardiovascular and circulatory diseases	3	B.2.11
508	**Chronic respiratory diseases**	2	B.3
509	Chronic obstructive pulmonary disease	3	B.3.1
510	Pneumoconiosis	3	B.3.2
515	Asthma	3	B.3.3
516	Interstitial lung disease and pulmonary sarcoidosis	3	B.3.4
520	Other chronic respiratory diseases	3	B.3.5
526	**Digestive diseases**	2	B.4
521	Cirrhosis and other chronic liver diseases	3	B.4.1
992	Upper digestive system diseases	3	B.4.2
529	Appendicitis	3	B.4.3
530	Paralytic ileus and intestinal obstruction	3	B.4.4
531	Inguinal, femoral, and abdominal hernia	3	B.4.5
532	Inflammatory bowel disease	3	B.4.6
533	Vascular intestinal disorders	3	B.4.7
534	Gallbladder and biliary diseases	3	B.4.8
535	Pancreatitis	3	B.4.9
541	Other digestive diseases	3	B.4.10
542	**Neurological disorders**	2	B.5
543	Alzheimer's disease and other dementias	3	B.5.1
544	Parkinson's disease	3	B.5.2
545	Idiopathic epilepsy	3	B.5.3
546	Multiple sclerosis	3	B.5.4
554	Motor neuron disease	3	B.5.5
972	Headache disorders	3	B.5.6
557	Other neurological disorders	3	B.5.7
558	**Mental disorders**	2	B.6
559	Schizophrenia	3	B.6.1
567	Depressive disorders	3	B.6.2
570	Bipolar disorder	3	B.6.3
571	Anxiety disorders	3	B.6.4
572	Eating disorders	3	B.6.5
575	Autism spectrum disorders	3	B.6.6
578	Attention-deficit/hyperactivity disorder	3	B.6.7
579	Conduct disorder	3	B.6.8
582	Idiopathic developmental intellectual disability	3	B.6.9
585	Other mental disorders	3	B.6.10
973	**Substance use disorders**	2	B.7
560	Alcohol use disorders	3	B.7.1
561	Drug use disorders	3	B.7.2
974	**Diabetes and kidney diseases**	2	B.8
587	Diabetes mellitus	3	B.8.1
589	Chronic kidney disease	3	B.8.2
588	Acute glomerulonephritis	3	B.8.3
653	**Skin and subcutaneous diseases**	2	B.9
654	Dermatitis	3	B.9.1
655	Psoriasis	3	B.9.2
980	Bacterial skin diseases	3	B.9.3
658	Scabies	3	B.9.4
659	Fungal skin diseases	3	B.9.5

660	Viral skin diseases	3	B.9.6
661	Acne vulgaris	3	B.9.7
662	Alopecia areata	3	B.9.8
663	Pruritus	3	B.9.9
664	Urticaria	3	B.9.10
665	Decubitus ulcer	3	B.9.11
668	Other skin and subcutaneous diseases	3	B.9.12
669	**Sense organ diseases**	2	B.10
981	Blindness and vision impairment	3	B.10.1
674	Age-related and other hearing loss	3	B.10.2
679	Other sense organ diseases	3	B.10.3
626	**Musculoskeletal disorders**	2	B.11
627	Rheumatoid arthritis	3	B.11.1
628	Osteoarthritis	3	B.11.2
630	Low back pain	3	B.11.3
631	Neck pain	3	B.11.4
632	Gout	3	B.11.5
639	Other musculoskeletal disorders	3	B.11.6
640	**Other non-communicable diseases**	2	B.12
641	Congenital birth defects	3	B.12.1
594	Urinary diseases and male infertility	3	B.12.2
603	Gynecological diseases	3	B.12.3
613	Hemoglobinopathies and hemolytic anemias	3	B.12.4
619	Endocrine, metabolic, blood, and immune disorders	3	B.12.5
680	Oral disorders	3	B.12.6
687	**Injuries**	1	C

Appendix table 2. Level 1 mortality, YLL, YLD and DALY rates per 100,000 population with 95% UI and respective percentage among age groups, both sexes, in the European Union, year 2019

Both sexes					
**Age**	**Cause**	**Rate per 100,000**	**95% UI**	**Percentage**	**95% UI**
**Mortality**					
**10 to 14**	Injuries	3.33	(3.12-3.56)	34.8%	(33.5-36.6)
	NCDs	5.57	(5.31-5.84)	58.2%	(56.1-59.6)
	CMNN	0.67	(0.61-0.74)	7.0%	(6.5-7.6)
**15 to 19**	Injuries	15.23	(14.48-15.92)	59.3%	(58.1-60.8)
	NCDs	9.47	(8.96-9.99)	36.9%	(35.3-38.1)
	CMNN	0.98	(0.91-1.07)	3.8%	(3.6-4.1)
**20 to 24**	Injuries	24.74	(23.75-25.69)	61.3%	(60.2-62.6)
	NCDs	14.30	(13.67-14.95)	35.5%	(34.2-36.6)
	CMNN	1.30	(1.21-1.40)	3.2%	(3.0-3.4)
**10 to 24**	Injuries	14.54	(13.92-15.11)	57.3%	(56.3-58.6)
	NCDs	9.83	(9.39-10.28)	38.8%	(37.4-39.8)
	CMNN	0.99	(0.92-1.06)	3.9%	(3.7-4.1)
**YLLs**					
**10 to 14**	Injuries	254.06	(238.26-271.48)	34.8%	(33.5-36.6)
	NCDs	424.70	(404.97-445.71)	58.2%	(56.1-59.6)
	CMNN	51.06	(46.85-56.2)	7.0%	(6.5-7.6)
**15 to 19**	Injuries	1086.52	(1032.88-1135.11)	59.3%	(58-60.8)
	NCDs	675.73	(639.10-712.49)	36.9%	(35.3-38.1)
	CMNN	70.16	(64.93-76.09)	3.8%	(3.6-4.1)
**20 to 24**	Injuries	1645.73	(1580.02-1709.46)	61.3%	(60.2-62.6)
	NCDs	951.77	(909.34-994.66)	35.5%	(34.2-36.6)
	CMNN	86.53	(80.64-93.45)	3.2%	(3.0-3.4)
**10 to 24**	Injuries	1001.65	(959.19-1041.2)	57.0%	(55.9-58.3)
	NCDs	686.93	(656.33-718.1)	39.1%	(37.7-40.1)
	CMNN	69.43	(64.99-74.32)	3.9%	(3.7-4.2)
**YLDs**					
**10 to 14**	Injuries	362.05	(243.22-523.20)	6.8%	(5.4-8.7)
	NCDs	4479.23	(3147.80-6062.40)	84.1%	(80.8-87.1)
	CMNN	476.84	(349.95-632.22)	9.0%	(7.3-11.0)
**15 to 19**	Injuries	521.45	(353.24-737.38)	6.9%	(5.4-8.7)
	NCDs	6652.70	(4689.92-9073.27)	87.1%	(84.1-89.5)
	CMNN	460.99	(341.14-602.98)	6.1%	(4.9-7.3)
**20 to 24**	Injuries	669.35	(456.14-951.65)	7.5%	(5.9-9.5)
	NCDs	7813.15	(5546.85-10334.38)	87.0%	(84.1-89.5)
	CMNN	487.00	(361.07-634.50)	5.5%	(4.4-6.7)
**10 to 24**	Injuries	519.18	(354.60-735.35)	7.1%	(5.6-8.9)
	NCDs	6328.51	(4489.66-8533.25)	86.4%	(83.5-88.8)
	CMNN	475.16	(354.68-615.77)	6.5%	(5.3-7.9)
**DALYs**					
**10 to 14**	Injuries	616.11	(496.77-777.49)	10.3%	(8.6-12.2)
	NCDs	4903.94	(3579.48-6485.61)	81.0%	(77.8-83.9)

	CMNN	527.89	(400.24-682.90)	8.8%	(7.2-10.5)
**15 to 19**	Injuries	1607.96	(1438.39-1827.32)	17.2%	(14.2-20.6)
	NCDs	7328.43	(5388.49-9756.02)	77.2%	(73.5-80.8)
	CMNN	531.15	(411.45-670.23)	5.6%	(4.7-6.6)
**20 to 24**	Injuries	2315.07	(2088.23-2596.69)	20.1%	(16.9-23.7)
	NCDs	8764.92	(6511.07-11291.52)	75.0%	(70.9-78.7)
	CMNN	573.53	(447.69-719.81)	4.9%	(4.1-5.8)
**10 to 24**	Injuries	1520.83	(1350.69-1742.36)	16.9%	(14.3-20.1)
	NCDs	7015.44	(5181.20-9224.35)	77.1%	(73.5-80.5)
	CMNN	544.59	(423.27-683.71)	6.0%	(5.1-7.0)

Abbreviations: NCDs=non-communicable diseases; CMNN=Communicable, maternal, neonatal, and nutritional diseases; UI=Uncertainty interval

Appendix table 3. Level 1 mortality, YLL, YLD and DALY rates per 100,000 population with 95% UI and respective percentage among age groups, by sex, in the European Union, year 2019

		Female				Male			
Age	Cause	Rate per 100,000	95% UI	Percentage	95% UI	Rate per 100,000	95% UI	Percentage	95% UI
**Mortality**									
10 to 14	Injuries	2.31	(2.16-2.51)	28.0%	(26.5-30.3)	4.30	(4.01-4.60)	39.7%	(37.9-41.4)
	NCDs	5.26	(5.01-5.49)	63.9%	(61.1-65.6)	5.86	(5.53-6.19)	54.1%	(52.0-56.0)
	CMNN	0.67	(0.61-0.74)	8.1%	(7.5-8.8)	0.67	(0.61-0.76)	6.2%	(5.7-6.9)
15 to 19	Injuries	7.50	(7.15-7.90)	46.3%	(44.7-48.4)	22.51	(21.30-23.62)	65.0%	(63.6-66.7)
	NCDs	7.78	(7.37-8.14)	48.0%	(45.8-49.5)	11.06	(10.37-11.79)	32.0%	(30.3-33.4)
	CMNN	0.93	(0.86-1.01)	5.7%	(5.3-6.2)	1.04	(0.95-1.14)	3.0%	(2.8-3.3)
20 to 24	Injuries	9.07	(8.65-9.57)	43.5%	(42.2-45.2)	39.46	(37.82-41.12)	67.3%	(66.0-68.6)
	NCDs	10.56	(10.09-11.06)	50.6%	(49.0-51.9)	17.82	(16.89-18.80)	30.4%	(29.1-31.7)
	CMNN	1.23	(1.13-1.33)	5.9%	(5.5-6.3)	1.37	(1.27-1.50)	2.3%	(2.2-2.5)
10 to 24	Injuries	6.31	(6.03-6.63)	41.6%	(40.5-43.3)	22.30	(21.27-23.28)	63.7%	(62.6-65.0)
	NCDs	7.89	(7.53-8.23)	52.1%	(50.3-53.3)	11.66	(11.04-12.28)	33.3%	(31.9-34.5)
	CMNN	0.94	(0.88-1.02)	6.2%	(5.9-6.6)	1.03	(0.95-1.11)	2.9%	(2.8-3.2)
**YLLs**									
10 to 14	Injuries	176.00	(165.00-192.00)	28.0%	(26.5-30.3)	328.00	(306.00-351.00)	39.7%	(37.9-41.4)
	NCDs	401.00	(382.00-419.00)	63.9%	(61.1-65.6)	447.00	(421.00-472.00)	54.1%	(52.0-56.0)
	CMNN	51.00	(46.00-56.00)	8.1%	(7.5-8.8)	51.00	(46.00-58.00)	6.2%	(5.7-6.9)
15 to 19	Injuries	536.00	(511.00-564.00)	46.3%	(44.7-48.4)	1,605.00	(1519.00-1684.00)	65.0%	(63.5-66.7)
	NCDs	556.00	(526.00-582.00)	48.0%	(45.8-49.5)	789.00	(739.00-841.00)	32.0%	(30.3-33.4)
	CMNN	66.00	(61.00-72.00)	5.7%	(5.3-6.2)	74.00	(67.00-82.00)	3.0%	(2.8-3.3)
20 to 24	Injuries	604.00	(576.00-637.00)	43.5%	(42.2-45.2)	2,625.00	(2516.00-2735.00)	67.3%	(66-68.6)
	NCDs	703.00	(671.00-736.00)	50.6%	(49-51.9)	1,185.00	(1124.00-1251.00)	30.4%	(29.1-31.7)
	CMNN	82.00	(76.00-89.00)	5.9%	(5.5-6.3)	91.00	(84.00-99.00)	2.3%	(2.2-2.5)
10 to 24	Injuries	439.12	(419.66-462.3)	41.4%	(40.3-43.1)	1532.14	(1460.68-1599.65)	63.4%	(62.3-64.7)
	NCDs	554.76	(529.28-578.84)	52.3%	(50.5-53.5)	811.56	(768.93-855.21)	33.6%	(32.2-34.7)
	CMNN	66.46	(61.93-71.63)	6.3%	(5.9-6.7)	72.22	(67.03-78.35)	3.0%	(2.8-3.2)
**YLDs**									
10 to 14	Injuries	331.38	(222.35-476.55)	5.7%	(4.4-7.3)	391.15	(261.02-565.16)	8.1%	(6.5-10.3)
	NCDs	5052.87	(3544.45-6890.10)	86.4%	(83.3-89.1)	3935.02	(2780.78-5326.53)	81.6%	(78-84.9)
	CMNN	459.18	(339.30-608.63)	7.9%	(6.3-9.9)	493.59	(361.08-662.99)	10.3%	(8.3-12.4)
15 to 19	Injuries	458.36	(313.10-638.03)	5.3%	(4.1-6.8)	580.78	(392.79-837.37)	8.8%	(7.0-11.0)
	NCDs	7802.60	(5516.82-10654.49)	89.3%	(86.7-91.4)	5571.20	(3969.89-7531.84)	84.3%	(81.0-87.0)
	CMNN	470.72	(344.85-620.14)	5.4%	(4.3-6.7)	451.84	(334.32-588.99)	6.9%	(5.6-8.2)
20 to 24	Injuries	558.52	(381.93-775.73)	5.3%	(4.2-6.8)	773.51	(521.46-1120.73)	10.3%	(8.2-12.9)
	NCDs	9445.38	(6618.66-12583.03)	89.7%	(87.1-91.8)	6279.01	(4478.24-8278.61)	83.6%	(80.1-86.5)
	CMNN	522.53	(384.30-692.87)	5.0%	(3.9-6.3)	453.60	(338.28-588.07)	6.1%	(5.0-7.3)
10 to 24	Injuries	450.32	(310.71-630.15)	5.4%	(4.2-6.8)	584.12	(397.39-833.70)	9.3%	(7.4-11.5)
	NCDs	7448.49	(5244.76-10136.18)	88.8%	(86.2-91.0)	5272.34	(3780.84-7046.90)	83.3%	(80-86.2)
	CMNN	484.59	(360.25-633.84)	5.8%	(4.7-7.1)	466.26	(347.29-605.38)	7.4%	(6.1-8.7)
**DALYs**									
10 to 14	Injuries	507.52	(400.87-649.91)	7.9%	(6.5-9.5)	719.13	(585.84-897.89)	12.8%	(10.8-15)
	NCDs	5454.17	(3951.32-7297.69)	84.2%	(81.2-86.9)	4381.92	(3229.90-5773.94)	77.5%	(74.2-80.8)
	CMNN	510.06	(389.06-657.74)	7.9%	(6.4-9.6)	544.81	(412.20-711.43)	9.7%	(8-11.4)

**15 to 19**	Injuries	993.88	(850.98-1172.28)	10.2%	(8.3-12.3)	2185.52	(1969.15-2436.38)	24.3%	(20.7-28.5)
	NCDs	8358.41	(6049.16-11219.17)	84.4%	(81.4-87.1)	6359.72	(4759.64-8325.88)	69.9%	(65.5-73.9)
	CMNN	537.00	(410.97-688.56)	5.4%	(4.4-6.5)	525.65	(405.39-659.69)	5.8%	(5.0-6.7)
**20 to 24**	Injuries	1162.16	(996.58-1379.65)	9.9%	(8.0-11.8)	3398.71	(3114.64-3738.21)	30.0%	(26.1-34.6)
	NCDs	10148.62	(7320-71-13283.54)	85.0%	(82.2-87.7)	7464.37	(5670.69-9457.84)	65.2%	(60.6-69.3)
	CMNN	604.34	(465.40-774.57)	5.1%	(4.1-6.2)	544.57	(428.67-678.72)	4.8%	(4.1-5.5)
**10 to 24**	Injuries	889.43	(751.20-1067.50)	9.5%	(7.8-11.4)	2116.25	(1906.90-2372.90)	24.4%	(21.1-28.4)
	NCDs	8003.25	(5812.78-10701.59)	84.6%	(81.8-87.3)	6083.91	(4576.63-7857.92)	69.4%	(65.2-73.3)
	CMNN	551.05	(426.32-699.92)	5.9%	(4.8-7.0)	538.49	(418.47-675.79)	6.2%	(5.3-7.0)

Abbreviations: NCDs=non-communicable diseases; CMNN=Communicable, maternal, neonatal, and nutritional diseases; UI=Uncertainty interval

Appendix table 4. Adolescents (10-24 years old) YLL rates in 1990 and 2019 and percentage change from 1990 to 2019

10-24 years old YLL rates per 100,000 population					
**Cause**	**1990**	**95% UI**	**2019**	**95% UI**	**Percentage change, % (95% UI)**
**All NCDs**	**1155.74**	**(1134.83-1171.34)**	**686.93**	**(656.33-718.10)**	**-40.56% (-43.16 to -37.74)**
**Neoplasms**	**445.71**	**(431.68-456.29)**	**281.80**	**(254.3-298.9)**	**-36.77% (-42.49 to -32.94)**
Lip and oral cavity cancer	2.56	(2.43-2.69)	1.70	(1.59-1.82)	-33.61% (-39.24 to -27.79)
Nasopharynx cancer	2.95	(2.77-3.12)	1.73	(1.56-1.93)	-41.24% (-47.89 to -33.18)
Other pharynx cancer	0.45	(0.41-0.5)	0.39	(0.34-0.46)	-13.23% (-25.91 to 3.01)
Esophageal cancer	0.68	(0.63-0.73)	0.50	(0.46-0.55)	-26.34% (-34.27 to -14.05)
Stomach cancer	6.61	(6.29-6.91)	2.28	(2.11-2.47)	-65.44% (-68.68 to-62.14)
Colon and rectum cancer	9.29	(8.96-9.64)	6.24	(5.77-6.72)	-32.9% (-38.42 to -27.10)
Liver cancer	5.67	(5.41-5.96)	4.9	(4.53-5.30)	-13.71% (-20.91 to -5.39)
Gallbladder and biliary tract cancer	0.40	(0.34-0.43)	0.27	(0.25-0.29)	-32.94% (-40.00 to -23.10)
Pancreatic cancer	2.02	(1.9-2.14)	1.62	(1.46-1.79)	-19.75% (-28.72 to -9.92)
Larynx cancer	0.51	(0.47-0.54)	0.28	(0.27-0.30)	-43.86% (-48.99 to -38.69)
Tracheal, bronchus, and lung cancer	8.70	(8.31-9.09)	5.57	(5.09-6.11)	-35.96% (-42.34 to -28.11)
Malignant skin melanoma	6.53	(4.77-8.27)	5.80	(3.70-6.72)	-11.32% (-37.26 to 7.04)
Non-melanoma skin cancer	0.66	(0.60-0.71)	0.43	(0.39-0.47)	-35.17% (-42.63 to -24.93)
Breast cancer	3.59	(3.41-3.78)	2.34	(2.15-2.54)	-34.87% (-41.02 to -28.19)
Cervical cancer	2.92	(2.27-3.20)	1.37	(1.15-1.57)	-52.97% (-59.63 to -44.39)
Uterine cancer	0.28	(0.25-0.30)	0.20	(0.18-0.22)	-28.43% (-36.44 to -18.94)
Ovarian cancer	7.36	(6.26-7.91)	4.86	(4.18-5.76)	-34.01% (-45.97 to -7.91)
Prostate cancer	0.47	(0.33-0.56)	0.35	(0.28-0.55)	-25.03% (-43.29 to 8.00)
Testicular cancer	12.52	(11.82-13.39)	6.68	(5.87-7.58)	-46.67% (-53.98 to -37.82)
Kidney cancer	3.95	(3.74-4.17)	3.78	(3.48-4.12)	-4.31% (-14.11 to 7.05)
Bladder cancer	0.80	(0.75-0.84)	0.57	(0.53-0.61)	-28.80% (-34.72 to -22.27)
Brain and central nervous system cancer	72.63	(61.34-88.4)	57.22	(37.71-64.02)	-21.22% (-55.73 to -9.95)
Thyroid cancer	1.55	(1.43-1.65)	0.90	(0.85-0.99)	-41.70% (-46.87 to -31.65)
Mesothelioma	0.39	(0.30-0.56)	0.33	(0.28-0.38)	-14.44% (-41.61 to 6.88)
Hodgkin lymphoma	20.67	(16.52-22.54)	7.86	(6.89-10.24)	-61.99% (-67.31 to -51.11)
Non-Hodgkin lymphoma	37.22	(35.78-38.62)	22.78	(21.2-24.98)	-38.80% (-43.75 to -32.22)
Multiple myeloma	0.34	(0.27-0.38)	0.32	(0.26-0.37)	-5.51% (-22.89 to 15.08)
Leukemia	124.51	(120.64-128.57)	64.64	(60.62-69.01)	-48.08% (-51.53 to -43.98)
Other neoplasms	1.93	(1.41-2.63)	1.86	(1.34-2.27)	-3.48% (-32.81 to 19.90)
Other malignant neoplasms	107.54	(97.26-112.18)	74.01	(62.15-80.22)	-31.18% (-40.95 to -25.49)
**CVDs**	**204.89**	**(199.36-210.24)**	**77.7**	**(73.20-82.30)**	**-62.08% (-64.47 to -59.74)**
Rheumatic heart disease	11.47	(10.76-12.12)	2.56	(2.28-2.84)	-77.66% (-80.37 to -74.86)
Ischemic heart disease	43.39	(41.28-45.58)	13.4	(12.25-14.9)	-69.12% (-72.09 to -65.05)
Stroke	80.10	(75.95-83.77)	21.97	(20.11-24.22)	-72.57% (-75.25 to -69.48)
Hypertensive heart disease	1.83	(1.40-2.09)	1.28	(0.94-1.53)	-30.35% (-45.87 to -7.05)
Non-rheumatic valvular heart disease	5.39	(4.96-5.79)	3.56	(3.23-3.95)	-33.99% (-42.26 to -23.90)
Cardiomyopathy and myocarditis	34.58	(30.41-39.52)	17.83	(15.54-21.24)	-48.42% (-55.57 to -36.50)
Aortic aneurysm	4.32	(3.96-4.66)	2.81	(2.54-3.10)	-34.92% (-42.30 to -25.43)
Endocarditis	2.97	(2.45-4.56)	3.36	(1.70-4.20)	13.27% (-55.23 to 62.05)
Other cardiovascular and circulatory diseases	20.84	(19.75-23.55)	10.90	(9.71-13.94)	-47.71% (-53.36 to -37.55)
**Chronic respiratory diseases**	**43.55**	**(39.04-45.8)**	**17.90**	**(16.60-20.50)**	**-58.83% (-62.32 to -50.19)**
Chronic obstructive pulmonary disease	9.02	(8.26-9.97)	5.09	(4.58-6.07)	-43.60% (-50.41 to -32.53)
Pneumoconiosis	0.18	(0.15-0.20)	0.08	(0.07-0.11)	-51.87% (-61.20 to -36.69)
Asthma	26.83	(23.43-28.48)	7.20	(6.56-8.26)	-73.17% (-75.82 to -66.85)

Interstitial lung disease and pulmonary sarcoidosis	2.86	(2.14-3.58)	3.02	(1.96-3.61)	5.66% (-30.72 to 31.99)
Other chronic respiratory diseases	4.67	(3.12-5.26)	2.54	(2.16-3.63)	-45.64% (-56.59 to -8.88)
**Digestive diseases**	**55.67**	**(54.02-57.39)**	**27.00**	**(25.30-28.70)**	**-51.57% (-54.78 to -48.26)**
Cirrhosis and other chronic liver diseases	26.93	(25.78-28.24)	10.44	(9.47-11.81)	-61.25% (-64.72 to -57.11)
Upper digestive system diseases	5.42	(5.07-5.76)	1.45	(1.30-1.63)	-73.30% (-76.16 to -69.92)
Appendicitis	3.54	(2.44-3.97)	1.06	(0.83-1.38)	-70.10% (-74.47 to -53.00)
Paralytic ileus and intestinal obstruction	5.09	(4.13-5.73)	3.85	(3.19-4.60)	-24.45% (-32.97 to -12.71)
Inguinal femoral and abdominal hernia	0.76	(0.65-0.82)	0.23	(0.21-0.26)	-69.89% (-73.15 to -63.25)
Inflammatory bowel disease	3.16	(2.86-3.90)	2.90	(2.42-3.32)	-8.22% (-35.21 to 6.24)
Vascular intestinal disorders	1.59	(1.44-1.79)	1.05	(0.95-1.19)	-33.78% (-42.58 to -23.03)
Gallbladder and biliary diseases	1.17	(0.84-1.30)	0.62	(0.42-0.70)	-47.39% (-55.74 to -33.84)
Pancreatitis	5.89	(5.35-6.44)	3.38	(2.97-3.79)	-42.65% (-50.72 to -32.19)
Other digestive diseases	2.11	(1.60-2.92)	1.99	(1.21-2.46)	-5.6% (-51.28 to 15.02)
**Neurological disorders**	**102.09**	**(99.20-105.28)**	**72.00**	**(67.80-76.40)**	**-29.49% (-34.31 to -24.43)**
Parkinson's disease	0.09	(0.08-0.10)	0.06	(0.05-0.07)	-31.47% (-43.54 to -16.58)
Idiopathic epilepsy	48.4	(46.4-50.92)	36.86	(33.68-39.59)	-23.84% (-31.53 to -17.60)
Multiple sclerosis	1.59	(1.01-1.83)	1.16	(0.75-1.53)	-27.03% (-41.11 to 13.80)
Motor neuron disease	4.47	(4.28-4.67)	3.91	(3.62-4.2)	-12.41% (-19.67 to -5.14)
Other neurological disorders	47.54	(45.65-49.31)	29.99	(27.94-32.10)	-36.92% (-41.61 to -31.41)
**Mental health disorders**	**0.93**	**(0.80-1.10)**	**1.23**	**(1.00-1.50)**	**32.18% (1.67 to 66.49)**
Eating disorders	0.93	(0.80-1.10)	1.23	(1.02-1.49)	32.18% (1.67 to 66.49)
**Substance use disorders**	**87.83**	**(83.7-92.35)**	**74.40**	**(68.6-81.80)**	**-15.28% (-23.45 to -4.58)**
Alcohol use disorders	12.92	(12.26-13.54)	9.38	(8.16-10.36)	-27.39% (-37.70 to -17.95)
Drug use disorders	74.91	(70.99-79.41)	65.02	(59.34-72.26)	-13.19% (-22.51 to -0.92)
**Diabetes and kidney diseases**	**31.64**	**(30.69-32.57)**	**15.4**	**(14.40-16.40)**	**-51.30% (-54.69 to -48.03)**
Diabetes mellitus	12.74	(12.21-13.21)	6.82	(6.42-7.28)	-46.47% (-50.14 to -42.84)
Chronic kidney disease	18.52	(17.82-19.25)	8.5	(7.81-9.18)	-54.12% (-57.83 to -50.03)
Acute glomerulonephritis	0.39	(0.33-0.46)	0.10	(0.08-0.11)	-75.20% (-80.13 to -68.90)
**Skin and subcutaneous diseases**	**1.40**	**(1.01-2.05)**	**1.30**	**(0.80-1.80)**	**-7.66% (-38.93 to 9.36)**
Bacterial skin diseases	0.81	(0.57-1.27)	1.03	(0.63-1.47)	26.95% (-26.09 to 53.66)
Decubitus ulcer	0.13	(0.05-0.19)	0.07	(0.02-0.10)	-48.25% (-67.47 to -29.80)
Other skin and subcutaneous diseases	0.46	(0.30-0.62)	0.20	(0.14-0.29)	-57.15% (-64.94 to -44.04)
Musculoskeletal disorders	9.58	(6.67-13.05)	6.30	(5.00-9.70)	-34.13% (-42.24 to -11.42)
Rheumatoid arthritis	1.02	(0.73-1.35)	0.45	(0.37-0.73)	-55.74% (-63.50 to -38.45)
Other musculoskeletal disorders	8.56	(5.93-11.71)	5.86	(4.64-8.95)	-31.55% (-39.95 to -7.92)
**Other NCDs**	**172.45**	**(150.83-186.93)**	**112**	**(101.5-131.70)**	**-35.08% (-39.90 to -21.72)**
Congenital birth defects	109.69	(88.98-121.73)	62.24	(50.97-78.48)	-43.25% (-49.06 to -22.06)
Urinary diseases and male infertility	6.53	(5.84-7.30)	3.57	(3.27-4.15)	-45.24% (-49.41 to -35.13)
Gynecological diseases	0.34	(0.28-0.38)	0.12	(0.10-0.22)	-65.59% (-71.30 to -43.36)
Hemoglobinopathies and hemolytic anemias	15.91	(15.38-16.43)	5.83	(5.33-6.41)	-63.35% (-66.87 to -59.23)
Endocrine, metabolic, blood, and immune disorders	39.98	(31.28-47.21)	40.18	(34.69-54.8)	0.5% (-8.20 to 24.28)

Abbreviations: NCDs=non-communicable diseases; CVDs=cardiovascular diseases; UI=Uncertainty interval

Appendix table 5. Adolescents (10-24 years old) YLD rates in 1990 and 2019 and percentage change from 1990 to 2019

10-24 years old YLD rates per 100,000 population					
**Cause**	**1990**	**95% UI**	**2019**	**95% UI**	**Percentage change, % (95% UI)**
**All NCDs**	**6238.86**	**(4431-50-8405.27)**	**6328.51**	**(4489.66-8533.25)**	**+1.44% (0.09 to 2.79)**
**Neoplasms**	**15.32**	**(10.73-20.61)**	**19**	**(13.00-26.00)**	**+22.09% (7.44 to 40.78)**
Lip and oral cavity cancer	0.10	(0.07-0.14)	0.08	(0.06-0.11)	-19.39% (-31.41 to -5.91)
Nasopharynx cancer	0.13	(0.09-0.17)	0.11	(0.08-0.16)	-9.31% (-27.27 to 12.04)
Other pharynx cancer	0.01	(0.01-0.01)	0.02	(0.01-0.02)	+96.44% (63.11 to 138.93)
Esophageal cancer	0.01	(0.00-0.01)	0.01	(0.00-0.01)	-0.38% (-16.28 to 20.92)
Stomach cancer	0.07	(0.05-0.09)	0.03	(0.02-0.05)	-47.23% (-54.83 to -38.58)
Colon and rectum cancer	0.31	(0.22-0.42)	0.34	(0.23-0.46)	+7.85% (-6.81 to 23.31)
Liver cancer	0.04	(0.03-0.06)	0.07	(0.04-0.10)	+57.88% (25.46 to 104.08)
Gallbladder and biliary tract cancer	0.00	(0.00-0.01)	0.00	(0.00-0.01)	-9.82% (-24.26 to 7.64)
Pancreatic cancer	0.01	(0.01-0.02)	0.01	(0.01-0.02)	-7.68% (-22.77 to 9.58)
Larynx cancer	0.08	(0.05-0.13)	0.08	(0.05-0.12)	-3.16% (-5.97 to -0.56)
Tracheal, bronchus, and lung cancer	0.06	(0.04-0.08)	0.05	(0.03-0.07)	-19.63% (-32.35 to -4.33)
Malignant skin melanoma	0.54	(0.32-0.80)	1.08	(0.59-1.63)	+102.04% (48.46 to 162.04)
Non-melanoma skin cancer	0.00	(0.00-0.00)	0.00	(0.00-0.00)	+7.23% (-1.65 to 14.82)
Breast cancer	0.31	(0.21-0.44)	0.34	(0.22-0.48)	+9.97% (-12.66 to 36.21)
Cervical cancer	0.17	(0.11-0.24)	0.12	(0.08-0.17)	-30.96% (-46.33 to -11.63)
Uterine cancer	0.04	(0.03-0.05)	0.04	(0.03-0.06)	+7.09% (-11.26 to 27.69)
Ovarian cancer	0.36	(0.24-0.50)	0.32	(0.21-0.46)	-12.07% (-31.88 to 21.83)
Prostate cancer	0.04	(0.02-0.06)	0.08	(0.05-0.12)	+80.15% (29.94 to 177.10)
Testicular cancer	1.48	(0.84-2.28)	1.94	(1.17-3.01)	+31.44% (-11.54 to 104.25)
Kidney cancer	0.17	(0.12-0.23)	0.21	(0.14-0.30)	+23.46% (5.12 to 43.64)
Bladder cancer	0.09	(0.06-0.12)	0.09	(0.06-0.12)	+2.81% (-11.48 to 20.10)
Brain and central nervous system cancer	0.93	(0.64-1.28)	1.24	(0.73-1.76)	+32.68% (-25.11 to 64.98)
Thyroid cancer	0.23	(0.15-0.33)	0.23	(0.15-0.33)	0% (-18.91 to 23.59)
Mesothelioma	0.00	(0.00-0.01)	0.00	(0.00-0.01)	-12.66% (-37.44 to 12.97)
Hodgkin lymphoma	1.43	(0.92-2.07)	1.58	(0.99-2.36)	+10.14% (-15.17 to 52.92)
Non-Hodgkin lymphoma	1.33	(0.93-1.80)	1.15	(0.78-1.63)	-13.78% (-27.97 to 2.55)
Multiple myeloma	0.01	(0.01-0.01)	0.01	(0.01-0.01)	+26.50% (-3.41 to 63.12)
Leukemia	2.95	(2.09-3.95)	4.00	(2.69-5.62)	+35.85% (11.33 to 65.87)
Other neoplasms	0.44	(0.25-0.73)	0.31	(0.18-0.51)	-29.39% (-39.08 to -16.99)
Other malignant neoplasms	3.97	(2.79-5.38)	5.16	(3.51-7.21)	+29.87% (9.24 to 58.23)
**CVDs**	**44.46**	**(29.97-61.31)**	**42.00**	**(28.00-58.00)**	**-6.03% (-9.66 to -2.32)**
Rheumatic heart disease	0.43	(0.26-0.65)	0.34	(0.20-0.52)	-20.90% (-28.53 to -13.13)
Ischemic heart disease	0.38	(0.20-0.67)	0.28	(0.15-0.50)	-26.23% (-33.63 to -19.68)
Stroke	26.87	(18.33-37.10)	25.58	(17.58-35.52)	-4.80% (-9.29 to -0.08)
Hypertensive heart disease	0.03	(0.01-0.05)	0.02	(0.01-0.04)	-17.33% (-22.40 to -12.62)
Non-rheumatic valvular heart disease	0.01	(0.01-0.02)	0.01	(0.00-0.02)	-14.72% (-23.77 to -5.40)
Cardiomyopathy and myocarditis	1.96	(1.07-3.28)	1.67	(0.90-2.80)	-14.85% (-20.00 to -9.97)
Endocarditis	0.06	(0.04-0.09)	0.05	(0.03-0.08)	-12.07% (-17.29 to -7.54)
Other cardiovascular and circulatory diseases	14.72	(7.57-25.06)	13.82	(7.26-23.43)	-6.09% (-12.28 to 0.40)
**Chronic respiratory diseases**	**271.80**	**(173.04-404.82)**	**269.00**	**(168.00-411.00)**	**-1.13% (-9.23 to 9.38)**
Chronic obstructive pulmonary disease	21.62	(16.46-26.88)	20.62	(15.51-25.79)	-4.67% (-12.41 to 3.50)
Pneumoconiosis	0.03	(0.02-0.05)	0.01	(0.01-0.03)	-46.53% (-61.03 to -33.73)
Asthma	243.75	(147.79-373.19)	235.41	(139.95-376.81)	-3.42% (-12.85 to 8.20)
Interstitial lung disease and pulmonary sarcoidosis	0.41	(0.21-0.70)	0.36	(0.19-0.61)	-11.00% (-15.33 to -5.93)
Other chronic respiratory diseases	5.99	(4.54-7.44)	12.33	(9.25-15.52)	+105.80% (88.67 to 123.89)

**Digestive diseases**	**78.14**	**(53.01-114.93)**	**76**	**(52.00-111.00)**	**-2.44% (-5.48 to 0.81)**
Cirrhosis and other chronic liver diseases	2.93	(1.69-4.66)	2.66	(1.55-4.16)	-9.26% (-19.85 to 2.63)
Upper digestive system diseases	37.05	(21.01-64.84)	34.74	(19.49-62.28)	-6.24% (-9.82 to -3.20)
Appendicitis	5.44	(3.12-8.96)	6.33	(3.77-10.06)	+16.27% (-0.76 to 40.86)
Paralytic ileus and intestinal obstruction	0.52	(0.34-0.71)	0.56	(0.37-0.76)	+7.56% (5.36 to 10.04)
Inguinal femoral and abdominal hernia	7.41	(4.30-11.79)	5.97	(3.50-9.44)	-19.35% (-28.93 to -9.03)
Inflammatory bowel disease	9.02	(5.68-12.85)	10.48	(6.76-14.95)	+16.16% (5.69 to 27.33)
Vascular intestinal disorders	0.2	(0.10-0.36)	0.2	(0.10-0.35)	-0.54% (-5.01 to 6.48)
Gallbladder and biliary diseases	13.4	(7.63-21.18)	13.28	(7.72-21.20)	-0.88% (-8.02 to 7.43)
Pancreatitis	0.73	(0.39-1.19)	0.71	(0.39-1.14)	-2.27% (-5.89 to 2.10)
Other digestive diseases	1.44	(0.96-2.01)	1.29	(0.88-1.79)	-9.81% (-14.29 to -5.54)
**Neurological disorders**	**894.08**	**(219.70-1916.98)**	**913**	**(223.00-1948.00)**	**+2.13% (-5.26 to 12.44)**
Parkinson's disease	0.02	(0.01-0.04)	0.02	(0.01-0.04)	+1.15% (-9.88 to 14.25)
Idiopathic epilepsy	104.36	(55.80-172.71)	103.32	(51.07-190.13)	-0.99% (-33.45 to 40.26)
Multiple sclerosis	4.02	(2.57-6.03)	4.77	(3.09-6.98)	+18.68% (7.25 to 33.02)
Motor neuron disease	0.72	(0.47-1.02)	0.77	(0.50-1.09)	+6.85% (2.83 to 11.56)
Headache disorders	761.17	(79.43-1792.62)	769.30	(78.52-1814.32)	+1.07% (-3.41 to 3.70)
Other neurological disorders	23.8	(13.19-38.52)	34.97	(17.87-62.57)	+46.97% (1.54 to 105.42)
**Mental health disorders**	**2007.11**	**(1419.72-2728.82)**	**2039.36**	**(1432.56-2773.47)**	**+1.61% (-0.62 to 3.86)**
Schizophrenia	63.18	(40.35-96.96)	60.24	(38.50-92.15)	-4.65% (-11.64 to 1.59)
Depressive disorders	587.35	(384.87-850.24)	569.42	(365.86-847.34)	-3.05% (-9.63 to 3.40)
Bipolar disorder	184.63	(100.79-295.70)	187.81	(102.26-301.80)	+1.72% (-1.93 to 5.58)
Anxiety disorders	612.59	(398.42-900.04)	641.37	(416.23-938.58)	+4.70% (0.65 to 8.99)
Eating disorders	129.63	(78.06-198.82)	149.23	(88.89-229.58)	+15.12% (9.42 to 20.44)
Autism spectrum disorders	89.21	(57.78-127.52)	92.27	(60.45-132.16)	+3.43% (0.55 to 6.17)
Attention-deficit/hyperactivity disorder	30.39	(17.04-52.32)	32.22	(17.80-55.80)	+6.03% (0.48 to 11.90)
Conduct disorder	227.6	(128.22-360.70)	234.52	(132.20-374.35)	+3.04% (0.84 to 5.27)
Idiopathic developmental intellectual disability	33.67	(15.13-57.43)	24.14	(9.31-42.55)	-28.29% (-38.34 to -23.60)
Other mental disorders	48.87	(26.04-79.52)	48.14	(25.31-77.72)	-1.49% (-6.71 to 3.85)
**Substance use disorders**	**404.5**	**(270.81-563.15)**	**430.0**	**(291.00-590.00)**	**+6.19% (1.21 to 12.73)**
Alcohol use disorders	191.54	(111.95-305.83)	177.84	(102.94-290.26)	-7.15% (-13.68 to -2.00)
Drug use disorders	212.96	(142.54-293.96)	251.69	(169.49-346.83)	+18.19% (10.06 to 27.97)
**Diabetes and kidney diseases**	**38.11**	**(26.59-53.04)**	**53.00**	**(35.00-73.00)**	**+37.80% (25.11 to 51.47)**
Diabetes mellitus	19.82	(12.65-29.31)	35.77	(22.66-53.43)	+80.50% (69.54 to 90.35)
Chronic kidney disease	18.26	(11.24-27.20)	16.72	(10.27-25.07)	-8.48% (-13.94 to -3.44)
Acute glomerulonephritis	0.02	(0.01-0.04)	0.02	(0.01-0.03)	-13.87% (-16.46 to -11.02)
**Skin and subcutaneous diseases**	**729.8**	**(485.89-1049.93)**	**774.00**	**(511.00-1117.00)**	**+6.00% (4.19 to 7.54)**
Dermatitis	171.24	(94.57-280.83)	180.58	(99.20-296.53)	+5.45% (3.14 to 7.84)
Psoriasis	100.98	(69.49-136.48)	93.69	(64.76-126.60)	-7.22% (-10.78 to -3.44)
Bacterial skin diseases	6.00	(2.81-11.48)	6.17	(2.92-11.71)	+2.95% (0.75 to 5.11)
Scabies	12.00	(6.39-19.95)	9.79	(5.21-16.32)	-18.44% (-20.76 to -16.15)
Fungal skin diseases	26.05	(9.86-57.17)	26.39	(9.98-57.99)	+1.32% (-0.10 to 2.72)
Viral skin diseases	82.32	(52.54-123.64)	85.86	(55.49-128.88)	+4.31% (2.61 to 6.07)
Acne vulgaris	253.02	(149.86-400.58)	293.19	(174.43-464.77)	+15.88% (13.70 to 18.20)
Alopecia areata	7.05	(4.44-10.68)	7.01	(4.39-10.61)	-0.49% (-5.86 to 5.53)
Pruritus	5.16	(2.34-9.82)	5.27	(2.40-10.08)	+2.21% (-0.84 to 5.66)
Urticaria	36.57	(22.68-55.92)	34.64	(21.53-53.01)	-5.28% (-8.54 to -1.74)
Decubitus ulcer	0.39	(0.24-0.59)	0.44	(0.28-0.65)	+11.34% (7.67 to 15.59)
Other skin and subcutaneous diseases	29.03	(13.67-54.42)	30.58	(14.36-57.27)	+5.35% (3.71 to 6.92)
**Sense organ diseases**	**161.3**	**(104.92-232.98)**	**150.00**	**(99.00-216.00)**	**-6.86% (-10.66 to -3.53)**
Blindness and vision loss	57.93	(35.68-88.02)	55.65	(33.94-85.36)	-3.95% (-7.47 to -1.02)
Age-related and other hearing loss	89.07	(53.43-131.61)	79.48	(48.09-118.58)	-10.77% (-16.00 to -5.55)

Other sense organ diseases	14.30	(7.93-23.33)	15.12	(8.43-24.67)	+5.73% (0.53 to 11.81)
**Musculoskeletal disorders**	**965.98**	**(661.26-1363.90)**	**968.00**	**(668.00-1371.00)**	**+0.20% (-1.95 to 2.49)**
Rheumatoid arthritis	8.17	(5.03-12.49)	9.35	(5.66-14.48)	+14.39% (4.59 to 24.23)
Low back pain	678.57	(439.08-992.73)	634.68	(410.14-938.51)	-6.47% (-8.88 to -3.94)
Neck pain	157.22	(89.41-267.77)	172.02	(97.60-290.28)	+9.41% (6.44 to 12.68)
Gout	0.35	(0.15-0.68)	0.38	(0.16-0.71)	+6.34% (2.60 to 13.07)
Other musculoskeletal disorders	121.67	(67.92-196.20)	151.48	(85.36-240.76)	+24.51% (17.38 to 34.87)
**Other NCDs**	**628.26**	**(424.18-902.48)**	**597.00**	**(405.00-854.00)**	**-5.02% (-7.12 to -2.89)**
Congenital birth defects	82.16	(56.43-114.10)	81.58	(56.79-110.93)	-0.71% (-3.63 to 2.38)
Urinary diseases and male infertility	7.92	(4.65-12.83)	7.80	(4.52-13.50)	-1.59% (-11.28 to 11.93)
Gynecological diseases	277.36	(180.59-410.22)	272.05	(176.31-402.57)	-1.91% (-4.79 to 1.29)
Hemoglobinopathies and hemolytic anemias	18.88	(11.47-30.03)	8.04	(4.90-12.65)	-57.39% (-65.32 to -47.69)
Endocrine, metabolic, blood, and immune disorders	143.26	(83.92-220.79)	136.25	(81.23-205.10)	-4.89% (-9.67 to 0.76)
Oral disorders	98.69	(53.76-163.39)	91.03	(50.27-150.71)	-7.76% (-10.76 to -4.85)

Abbreviations: NCDs=non-communicable diseases; CVDs=cardiovascular diseases; UI=Uncertainty interval

Appendix table 6. Socio-demographic index in 2019 for the 28 European Union Member States

Countries	SDI 2019
Austria	0.85
Belgium	0.85
Bulgaria	0.76
Croatia	0.79
Cyprus	0.84
Czechia	0.83
Denmark	0.89
Estonia	0.84
Finland	0.86
France	0.83
Germany	0.90
Greece	0.79
Hungary	0.79
Ireland	0.87
Italy	0.80
Latvia	0.82
Lithuania	0.84
Luxembourg	0.90
Malta	0.80
Netherlands	0.88
Poland	0.80
Portugal	0.74
Romania	0.76
Slovakia	0.81
Slovenia	0.84
Spain	0.77
Sweden	0.87
United Kingdom	0.85

Appendix table 7. Spearman rank-correlation coefficients and relative P-values for the association between DALY rates of level 2 NCDs and SDI in the 28 European Union Member States, both sexes, age 10-24, year 2019

**Spearman rank-correlation coefficient (P-value)**	
	**SDI**
**CRDs**	0.303 (0.117)
**CVDs**	**-0.455* (0.015)**
**DM&KD**	-0.238 (0.223)
**DD**	-0.358 (0.061)
**MDs**	0.230 (0.239)
**MSDs**	0.338 (0.078)
**Neo**	**-0.572* (0.001)**
**Neuro**	0.368 (0.054)
**Other NCDs**	0.160 (0.417)
**SOD**	**-0.614* (0.001)**
**SSDs**	**0.447* (0.017)**
**SUD**	**0.579* (0.001)**

Abbreviations: CRDs = Chronic respiratory diseases; CVDs = Cardiovascular diseases; DM&KD = Diabetes and kidney diseases; DD = Digestive diseases; MDs = Mental health disorders; MSDs = Musculoskeletal disorders; Neuro = Neurological disorders; Neo = Neoplasms; other NCDs = other Non-communicable diseases; SDI = Socio-Demographic Index SOD = Sense organ diseases; SSDs = Skin and subcutaneous diseases; SUD = Substance use disorders.
Note: *p-value < 0.05

Contributions

Providing data or critical feedback on data sources

Derrick Bary Abila, Hassan Abolhassani, Victor Adekanmbi, Hanadi Al Hamad, Benedetta Armocida, Sameh Attia, Marcel Ausloos, Jennifer L Baker, Sandra Barteit, Sanjay Basu, David Beran, Boris Bikbov, Giovanni Damiani, Alejandro de la Torre-Luque, Mostafa Dianatinasab, Diana Dias da Silva, Abdel Douiri, Elena Dragioti, Luchuo Engelbert Bain, Santosh Gaihre, James C Glasbey, Vijai Kumar Gupta, Josep Maria Haro, Claudiu Herteliu, Gaetano Isola, Jost B Jonas, Jacek Jerzy Jozwiak, Mikk Jürisson, Zubair Kabir, André Karch, Joonas H Kauppila, Gbenga A Kayode, Moien AB Khan, Khaled Khatab, Jitka Klugarová, Dian Kusuma, Rubén López-Bueno, Stefan Lorkowski, Konstantinos Christos Makris, Manu Raj Mathur, Ritesh G Menezes, Atte Meretoja, Marcello Moccia, Shafiu Mohammed, Mariam Molokhia, Lorenzo Monasta, Ulrich Otto Mueller, Francesk Mulita, Ionut Negoi, Ruxandra Irina Negoi, Bogdan Oancea, Ayodipupo Sikiru Oguntade, Alberto Ortiz, Adrian Pana, Demosthenes Panagiotakos, Songhomitra Panda-Jonas, Shahina Pardhan, Maja Pasovic, Jay Patel, José L Peñalvo, Renato B Pereira, Suzanne Polinder, Maarten J Postma, Mohammad Rabiee, Navid Rabiee, David Laith Rawaf, Salman Rawaf, Luca Ronfani, Mohammad Reza Saeb, Brijesh Sathian, Sonia Saxena, João Pedro Silva, Rafael Tabarés-Seisdedos, Marcos Roberto Tovani-Palone, Thomas Clement Truelsen, Tommi Juhani Vasankari, Santos Villafaina, Ronny Westerman, Sanni Yaya, Vesna Zadnik, and Alimuddin Zumla.

Developing methods or computational machinery

Mostafa Dianatinasab, Gaetano Isola, Marcello Moccia, Mohammad Rabiee, Navid Rabiee, Mohammad Reza Saeb, and Ronny Westerman.

Providing critical feedback on methods or results

Hassan Abolhassani, Manfred Mario Kokou Accrombessi, Victor Adekanmbi, Keivan Ahmadi, Hanadi Al Hamad, Adel Al-Jumaily, Robert Ancuceanu, Catalina Liliana Andrei, Tudorel Andrei, Benedetta Armocida, Ashokan Arumugam, Sameh Attia, Avinash Aujayeb, Marcel Ausloos, Jennifer L Baker, Fabio Barra, Sandra Barteit, Sanjay Basu, Bernhard T Baune, Derrick A Bennett, David Beran, Boris Bikbov, Andras Bikov, Oleg Blyuss, Hermann Brenner, Flavia Bustreo, Giulia Carreras, Giulio Castelpietra, Alberico L Catapano, Joht Singh Chandan, Periklis Charalampous, Joao Conde, Natália Cruz-Martins, Giovanni Damiani, Alejandro de la Torre-Luque, Mostafa Dianatinasab, Diana Dias da Silva, Abdel Douiri, Elena Dragioti, Luchuo Engelbert Bain, Adeniyi Francis Fagbamigbe, Seyed-Mohammad Fereshtehnejad, José Miguel P Ferreira de Oliveira, Simone Ferrero, Lorenzo Ferro Desideri, Florian Fischer, Piyada Gaewkhiew, Santosh Gaihre, Paramjit Singh Gill, James C Glasbey, Vijai Kumar Gupta, Mekdes Kondale Gurara, Josep Maria Haro, M Tasdik Hasan, Rasmus J Havmoeller, Behzad Heibati, Merel E Hellemons, Claudiu Herteliu, Salman Hussain, Gaetano Isola, Olatunji Johnson, Jost B Jonas, Jacek Jerzy Jozwiak, Mikk Jürisson, Zubair Kabir, André Karch, Joonas H Kauppila, Gbenga A Kayode, Moien AB Khan, Khaled Khatab, Mika Kivimäki, Miloslav Klugar, Jitka Klugarová, Kamrun Nahar Koly, Ai Koyanagi, Om P Kurmi, Dian Kusuma, Carlo La Vecchia, Tea Lallukka, Demetris Lamnisos, Anders O Larsson, Paolo Lauriola, Matilde Leonardi, An Li, Rubén López-Bueno, Stefan Lorkowski, Joana A Loureiro, Laura A Magee, Francesca Giulia Magnani, Konstantinos Christos Makris, Alexander G Mathioudakis, Manu Raj Mathur, Ritesh G Menezes, Alexios-Fotios A Mentis, Atte Meretoja, Junmei Miao Jonasson, Tomasz Miazgowski, Andreea Mirica, Marcello Moccia, Shafiu Mohammed, Mariam Molokhia, Lorenzo Monasta, Stefania Mondello, Ulrich Otto Mueller, Francesk Mulita, Ionut Negoi, Ruxandra Irina Negoi, Evangelia Nena, Vincent Ebuka Nwatah, Bogdan Oancea, Ayodipupo Sikiru Oguntade, Alberto Ortiz, Adrian Otoiu, Raffaele Palladino, Adrian Pana, Songhomitra Panda-Jonas, Shahina Pardhan, Jay Patel, Paolo Pedersini, José L Peñalvo, Renato B Pereira, Pablo Perel, Ionela-Roxana Petcu, Maarten J Postma, Mohammad Rabiee, Navid Rabiee, Alberto Raggi, Shadi Rahimzadeh, David Laith Rawaf, Salman Rawaf, Abanoub Riad, Alina Rodriguez, Luca Ronfani, Mohammad Reza Saeb, Mahdi Safdarian, Brijesh Sathian, Susan Sawyer, Sonia Saxena, Nikolaos Scarmeas, Winfried Schlee, Falk Schwendicke, Giulia Segafredo, Morteza Shamsizadeh, Nigussie Tadesse Sharew, Rahman Shiri, Siddharudha Shivalli, Velizar Shivarov, Søren T Skou, Bogdan Socea, Ireneous N Soyiri, Paschalis Steiropoulos, Kurt Straif, Xiaohui Sun, Rafael Tabarés-Seisdedos, Arulmani Thiyagarajan, Fotis Topouzis, Marcos Roberto Tovani-Palone, Thomas Clement Truelsen, Jef Van den Eynde, Massimiliano Veroux, Santos Villafaina, Francesco S Violante, Victor Volovici, Yanzhong Wang, Ronny Westerman, Mohammad Esmaeil Yadegarfar, Sanni Yaya, Vesna Zadnik, and Alimuddin Zumla.

Drafting the work or revising is critically for important intellectual content
Hassan Abolhassani, Victor Adekanmbi, Mamoon A Aldeyab, Adel Al-Jumaily, Robert Ancuceanu, Benedetta Armocida, Ashokan Arumugam, Sameh Attia, Avinash Aujayeb, Marcel Ausloos, Jennifer L Baker, Francesco Barone-Adesi, Fabio Barra, Sandra Barteit, Sanjay Basu, Bernhard T Baune, Yannick Béjot, Luis Belo, David Beran, Boris Bikbov, Andras Bikov, Susanne Breitner, Hermann Brenner, Flavia Bustreo, Márcia Carvalho, Giulio Castelpietra, Alberico L Catapano, Joht Singh Chandan, Simiao Chen, Joao Conde, Natália Cruz-Martins, Giovanni Damiani, Anna Dastiridou, Mostafa Dianatinasab, Diana Dias da Silva, Abdel Douiri, Adeniyi Francis Fagbamigbe, Seyed-Mohammad Fereshtehnejad, Pietro Ferrara, José Miguel P Ferreira de Oliveira, Simone Ferrero, Lorenzo Ferro Desideri, Florian Fischer, Diogo A Fonseca, Piyada Gaewkhiew, Santosh Gaihre, Silvano Gallus, Mariana Gaspar Fonseca, Paramjit Singh Gill, James C Glasbey, Giuseppe Gorini, Josep Maria Haro, M Tasdik Hasan, Rasmus J Havmoeller, Behzad Heibati, Claudiu Herteliu, Salman Hussain, Gaetano Isola, Jost B Jonas, Jacek Jerzy Jozwiak, Mikk Jürisson, André Karch, Joonas H Kauppila, Gbenga A Kayode, Moien AB Khan, Khaled Khatab, Mika Kivimäki, Miloslav Klugar, Ai Koyanagi, Om P Kurmi, Dian Kusuma, Carlo La Vecchia, Ben Lacey, Tea Lallukka, Berthold Langguth, Anders O Larsson, Paul H Lee, Matilde Leonardi, An Li, Christine Linehan, Rubén López-Bueno, Stefan Lorkowski, Joana A Loureiro, Raimundas Lunevicius, Laura A Magee, Francesca Giulia Magnani, Azeem Majeed, Alexander G Mathioudakis, John J McGrath, Ritesh G Menezes, Alexios-Fotios A Mentis, Atte Meretoja, Tomislav Mestrovic, Tomasz Miazgowski, Marcello Moccia, Shafiu Mohammed, Mariam Molokhia, Lorenzo Monasta, Stefania Mondello, Ulrich Otto Mueller, Francesk Mulita, Daniel Munblit, Ionut Negoi, Ruxandra Irina Negoi, Nurulamin M Noor, Christoph Nowak, George Ntaios, Vincent Ebuka Nwatah, Bogdan Oancea, Ayodipupo Sikiru Oguntade, Alberto Ortiz, Adrian Otoiu, Alicia Padron-Monedero, Raffaele Palladino, Adrian Pana, Demosthenes Panagiotakos, Songhomitra Panda-Jonas, Shahina Pardhan, Maja Pasovic, Jay Patel, Paolo Pedersini, José L Peñalvo, Umberto Pensato, Renato B Pereira, Pablo Perel, Norberto Perico, Ionela-Roxana Petcu, Suzanne Polinder, Maarten J Postma, Alberto Raggi, David Laith Rawaf, Salman Rawaf, Faizan Ur Rehman, Giuseppe Remuzzi, Alina Rodriguez, Simona Sacco, Mahdi Safdarian, Davide Sattin, Susan Sawyer, Sonia Saxena, Winfried Schlee, Giulia Segafredo, Siddharudha Shivalli, João Pedro Silva, Colin R Simpson, Søren T Skou, Bogdan Socea, Ireneous N Soyiri, Paschalis Steiropoulos, Kurt Straif, Xiaohui Sun, Arulmani Thiyagarajan, Marcos Roberto Tovani-Palone, Thomas Clement Truelsen, Brigid Unim, Jef Van den Eynde, Tommi Juhani Vasankari, Massimiliano Veroux, Santos Villafaina, Matej Vinko, Francesco S Violante, Victor Volovici, Yanzhong Wang, Ronny Westerman, Mohammad Esmaeil Yadegarfar, and Sanni Yaya.

Managing the overall research enterprise
Simon I Hay and Maja Pasovic.
